# A transcriptome-wide antitermination mechanism sustaining identity of embryonic stem cells

**DOI:** 10.1038/s41467-019-14204-z

**Published:** 2020-01-17

**Authors:** Yaroslav A. Kainov, Eugene V. Makeyev

**Affiliations:** Centre for Developmental Neurobiology, King’s College London, London, SE1 1UL UK

**Keywords:** RNA, Differentiation, Pluripotency, Self-renewal, Stem cells

## Abstract

Eukaryotic gene expression relies on extensive crosstalk between transcription and RNA processing. Changes in this composite regulation network may provide an important means for shaping cell type-specific transcriptomes. Here we show that the RNA-associated protein Srrt/Ars2 sustains embryonic stem cell (ESC) identity by preventing premature termination of numerous transcripts at cryptic cleavage/polyadenylation sites in first introns. Srrt interacts with the nuclear cap-binding complex and facilitates recruitment of the spliceosome component U1 snRNP to cognate intronic positions. At least in some cases, U1 recruited in this manner inhibits downstream cleavage/polyadenylation events through a splicing-independent mechanism called telescripting. We further provide evidence that the naturally high expression of Srrt in ESCs offsets deleterious effects of retrotransposable sequences accumulating in its targets. Our work identifies Srrt as a molecular guardian of the pluripotent cell state.

## Abstract (web summary)

Besides its role in splicing, U1 snRNP can suppress pre-mRNA cleavage and polyadenylation. The authors show that the nuclear cap-binding complex component Srrt/Ars2 maintains embryonic stem cell identity by promoting U1 recruitment to first introns and preventing premature termination of multiple transcripts.

## Introduction

Eukaryotes are characterized by a remarkable degree of coordination between different steps of their gene expression program^1,2^. Most mRNA precursors (pre-mRNAs) are modified by the addition of a 7-methylguanosine cap to the 5′ end, excision of introns by the spliceosome, and 3′-terminal cleavage and polyadenylation. Aberrant RNA species are degraded by specialized quality control mechanisms. All these events can occur co-transcriptionally, receiving regulatory inputs from elongating RNA polymerase II (Pol II) but also modulating the efficiency of RNA synthesis through various forms of functional feedback^3–7^.

Co-transcriptional capping of Pol II transcripts followed by the assembly of the nuclear cap-binding complex (CBC) provides a critical line of communication between RNA synthesis and subsequent processing events^8,9^. The two core subunits of the CBC, Ncbp1/Cbc80 and the Ncbp2/Cbc20, can recruit several additional co-factors including the conserved multipurpose adapter protein Srrt/Ars2 (refs. ^10–13^). Srrt has been shown to mediate degradation of promoter-proximal transcripts in an exosome-dependent manner, promote termination/3′-terminal maturation of replication-dependent histone mRNAs and several other Pol II transcripts, and control production of small noncoding RNAs^10–12,14–16^. Of note, CBC can stimulate pre-mRNA splicing by recruiting U1 snRNP and other components of the spliceosome complex to cap-proximal introns^17–19^, but whether this activity depends on Srrt is an open question.

Unlike the core CBC components expressed at relatively stable levels across different conditions, Srrt tends to be substantially more abundant in proliferating cells than in their differentiated or quiescent counterparts. Consistent with this behavior, Srrt has been shown to promote proliferation of mammalian cells both in vitro and in vivo^14,20,21^. These effects may be facilitated by the microRNA or/and histone mRNA regulation activities of Srrt^10,14,22,23^. On the other hand, Srrt contributes to maintenance of mouse neural stem cells (NSCs) in a microRNA-independent manner, by promoting expression of the critical transcription factor Sox2 (ref. ^24^). Notably, Srrt is critical for early development in vertebrates^25,26^. However, molecular mechanisms underlying this effect remain poorly understood.

Pre-mRNA cleavage and polyadenylation is another crucial point of gene regulation. These two coupled reactions involve co-transcriptional assembly of multisubunit protein complexes at a 6-nt polyadenylation signal (PAS) and its adjacent sequences, cleavage of the nascent transcript at the cleavage/polyadenylation site (CS) located typically 10–30 nt downstream of the PAS, and subsequent addition of a poly(A) tail to the newly formed 3′ end^27–29^. Co-transcriptional cleavage/polyadenylation triggers a rapid release of the elongating Pol II complex from the DNA template^30^.

Interestingly, recruitment of U1 snRNP to 5′ splice sites (5′ss) or other cognate motifs can repress downstream CSs through a splicing-independent mechanism known as telescripting^31,32^. Telescripting is required for normal expression of relatively long mammalian genes^33^, and its efficiency can be modulated by global changes in transcriptional activity of the cell altering the ratio between free and pre-mRNA-associated U1 (ref. ^32^). However, it is unclear if telescripting can be controlled in a more nuanced cell type-specific manner. Similarly, the emerging link between telescripting and early steps of Pol II elongation awaits further experimental characterization^34–36^.

Embryonic stem cells (ESCs) are developmentally early progenitors capable of self-renewal and differentiation into the three germ layers of the embryo proper. Several transcription factors including Pou5f1/Oct4, Nanog, and Sox2 are known to play a key part in specifying molecular identity of this and other types of pluripotent stem cells^37–39^. Here we identify Srrt as a top candidate in a screen for additional regulators involved in ESC maintenance. We show that Srrt functions in this context by suppressing premature termination of transcription at cryptic cleavage/polyadenylation sites in first introns. This mechanism affects hundreds of genes active in ESCs and is mediated by CBC-dependent recruitment of U1 snRNP to 5′-proximal pre-mRNA sequences. In addition to its possible contribution to evolutionarily conserved gene regulation events, this activity limits deleterious effect of retrotransposable elements (RTEs) accumulating in first introns of its target genes. Overall, our work uncovers a transcriptome-wide antitermination circuitry with important roles in ESC biology.

## Results

### ESC maintenance depends on naturally high expression of Srrt

**Fig. 1:**
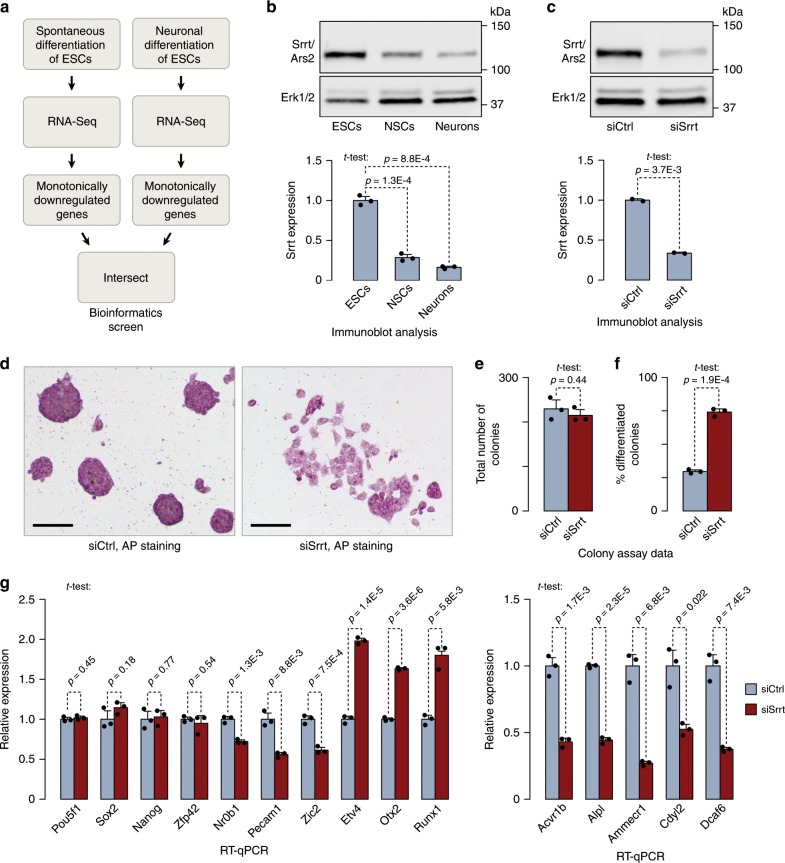
Srrt is required for mouse ESC maintenance. **a** Bioinformatics workflow used to identify putative regulators of mouse ESC identity. **b** Top: immunoblot analysis of Srrt expression in mouse ESCs, cortical NSCs, and cortical neurons prepared and cultured in vitro as described^86^. Bottom: Srrt protein expression was quantified from three independent experiments (mean ± SD) and compared using a two-tailed *t*-test. **c** Top: ESCs were transfected with an Srrt-specific siRNA mixture (siSrrt) or a non-targeting control siRNA (siCtrl) and Srrt knockdown efficiency was analyzed by immunoblotting 48 h later. Bottom: the experiment was repeated twice (mean ± SD) and the samples were compared using a two-tailed *t*-test. **b**, **c** Erk1/2 is a lane loading control. **d** ESCs were transfected with siSrrt as in **c** and assayed for alkaline phosphatase (AP) activity. Note pronounced changes in morphology of colonies and individual cells and a decrease in the AP staining intensity. Scale bar, 100 μm. **e**, **f** Colony assay data showing that **e** siSrrt does not change the overall number of ESC colonies but **f** significantly increases the fraction of flattened differentiated colonies compared to siCtrl. The assay was repeated three times (mean ± SD) and analyzed by a two-tailed *t*-test. **g** Left: RT-qPCR data showing that, while Srrt knockdown does not change expression of pluripotency markers Pou5f1, Sox2, Nanog, and Zfp42/Rex1, it leads to significant downregulation of Nr0b1, Pecam1, and Zic2 and upregulation of early differentiation markers Etv4, Otx2, and Runx1 (refs. ^39,43,87^). Right: targets strongly downregulated by siSrrt include additional examples of known ESC markers and factors with possible regulatory roles in proliferating cells^43–47^. All RT-qPCR experiments were done at least in triplicate and shown as mean ± SD. The expression levels in siCtrl-treated samples were set to 1, and the *p* values were calculated using a two-tailed *t*-test. Source data are provided as a Source Data file.

To understand possible role of RNA-based regulation mechanisms in maintenance of mouse ESCs, we inspected genes downregulated during neuronal and spontaneous differentiation of this cell type^40,41^ (Fig. 1a). A stringent shortlisting procedure identified 84 top candidates with expression levels decreasing monotonically in both differentiation models (Supplementary Data 1). The list contained several previously characterized ESC-enriched transcription factors including but not limited to Pou5f1/Oct4 and Sox2 (Supplementary Data 1). Among putative regulators of RNA processing Srrt was a particularly promising candidate since its knockout (KO) results in preimplantation embryonic lethality^25^ but its role in ESCs, i.e. cells matching this stage of mouse development, has not been investigated systematically.

Srrt protein was readily detectable in mouse ESCs and its levels were substantially reduced in proliferating NSCs [fold change (FC) = 2.9; *t*-test *p* = 1.3e-04] and post-mitotic neurons (FC = 5.8; *t*-test *p* = 8.8e-04; Fig. 1b). Srrt expression was also downregulated upon withdrawal of 2i inhibitors and LIF, the compounds required to maintain ESCs in an undifferentiated naïve state (Supplementary Fig. 1a, b; FC = 2.4; *t*-test *p* = 0.034; ref. ^42^). Of note, the expression of the CBC subunit Ncbp1 remained constant under these conditions (Supplementary Fig. 1a, b; *t*-test *p* = 0.78).

To address functional significance of the naturally high expression of Srrt in ESCs, we downregulated it to a level comparable to that observed in more differentiated cells using a mixture of four Srrt-specific siRNAs (siSrrt; Fig. 1c; compare with Fig. 1b and Supplementary Fig. 1a, b). This led to a loss of the characteristic rounded morphology of ESC colonies and reduced ESC-specific alkaline phosphatase activity compared to cultures treated with a control siRNA (siCtrl; Fig. 1d). Srrt knockdown also led to a readily detectable differentiation effect in a colony formation assay (Fig. 1e, f, Supplementary Fig. 1c–f). Moreover, siSrrt triggered a modest but statistically significant decrease in the expression of ESC-enriched surface markers SSEA1 and Pecam1/CD31 (Supplementary Fig. 1g, h). This suggests that maintenance of ESCs depends on relatively high expression of Srrt.

### Srrt knockdown has a global effect on the ESC transcriptome

RNA-sequencing (RNA-Seq) analysis uncovered considerable changes in the transcriptome of siSrrt-treated ESCs with 1828 downregulated and 1590 upregulated genes [FC ≥ 1.5 and false discovery rate (FDR) < 0.05; Supplementary Data 2]. The regulated genes showed a partial overlap with those changing their expression during spontaneous differentiation of ESCs (Supplementary Fig. 2a). Although expression of many pluripotency markers including Pou5f1/Oct4, Sox2, and Nanog remained unchanged in response to siSrrt, some examples of this category (e.g. Nr0b1, Pecam1, and Zic2) were detectably downregulated (Supplementary Fig. 2b). Conversely, expression of many developmental and differentiation markers increased (Supplementary Fig. 2b), in line with enrichment of corresponding gene ontology (GO) terms among the upregulated genes (Supplementary Data 3). For example, the GO terms developmental process, multicellular organismal development, and cell differentiation were enriched with FDRs 3.6E-6, 7.4E-6, and 1.5E-5, respectively (Supplementary Data 3). We confirmed RNA-Seq expression data for 20 pluripotency and differentiation markers selected for RT-qPCR validation (Fig. 1g, Supplementary Fig. 1c).

Notably, downregulated genes were over-represented among the most reliable changes triggered by siSrrt (Supplementary Fig. 2d). Although we did not detect significantly enriched GO terms for this category of genes, some of the especially robust downregulation targets (FC ≥ 2 and FDR < 1E-50; dark red dots in Supplementary Fig. 2d) encoded known ESC markers and positive regulators of cell proliferation. Relevant examples included alkaline phosphatase Alpl (the enzyme assayed in Fig. 1d and Supplementary Fig. 1c–e), epigenetic regulator Cdyl2, activin receptor Acvr1b/Alk4, nuclear receptor co-activator Dcaf6/NRIP, and a conserved RAGNYA domain protein Ammecr1 mutated in the Alport syndrome with mental retardation, midface hypoplasia, and elliptocytosis^43–47^. Downregulation of these genes was confirmed by RT-qPCR (Fig. 1g). Thus, Srrt may help ESCs to maintain their undifferentiated status by regulating extensive sets of genes.

### Srrt limits expression of prematurely terminated transcripts

**Fig. 2:**
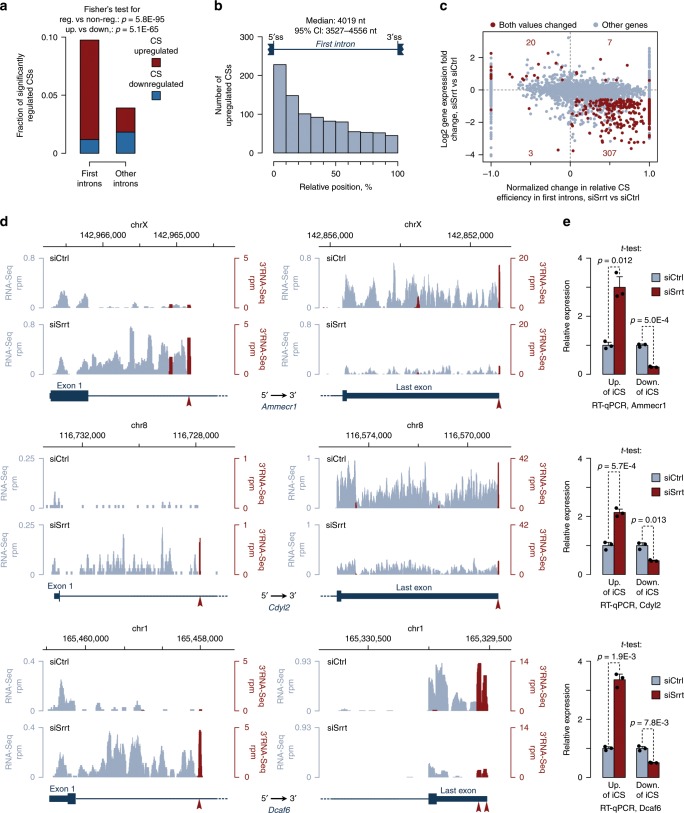
Srrt blocks cleavage/polyadenylation in first introns of many genes. **a** Srrt knockdown in mouse ESCs promotes utilization of cryptic CSs in first introns. **b** Upregulated CSs tend to localize close to the 5′ end of fist introns. **a**, **b** CSs with FC ≥ 2 and FDR < 0.05 were considered significantly regulated. **c** Scatter plot showing that siSrrt-mediated activation of intronic CSs strongly correlates with downregulation of gene expression. Genes with significant changes in relative CS efficiency in first introns (FDR < 0.05) and expression levels (FC ≥ 1.5 and FDR < 0.05) are shown in red. Other genes, gray. **d** Examples of genes regulated by Srrt via intronic cleavage/polyadenylation. Read-per-million (rpm)-normalized RNA-Seq coverage plots are shown in gray, and rpm-normalized 3′RNA-Seq data are in red. Note simultaneous activation of CSs in first introns and a decrease in RNA-Seq and 3′RNA-Seq signals in the corresponding 3′ untranslated regions (3′UTRs). Red arrowheads, CSs preceded by canonical polyadenylation signals (PASs), AATAAA, or ATTAAA. **e** RT-qPCR verification of the siSrrt effect on genes in **d** using primer pairs designed against sequences upstream or downstream of regulated iCSs. Gene-specific signals were normalized to Cnot4 housekeeping gene and the expression levels in siCtrl-treated sample were set to 1. Data were averaged from three experiments ± SD and compared by a two-tailed *t*-test. Source data are provided as a Source Data file.

We noticed that many genes responded to Srrt knockdown by accumulating RNA-Seq reads in first (5′-proximal) introns (Supplementary Fig. 3a). This often coincided with downregulation of the corresponding genes (the lower right quadrant in Supplementary Fig. 3b and the blue line in Supplementary Fig. 3c) and when it did, the increase in the RNA-Seq coverage was strongly biased towards the 5′ end of the first intron (Supplementary Fig. 3d). Relevant examples included the genes in the right plot in Fig. 1g (see below). To check if this behavior could be due to premature termination of transcription, we mapped the position of cleavage/polyadenylation sites (CSs) using 3′-proximal RNA-sequencing (3′RNA-Seq). This revealed a widespread activation of CSs within first introns in siSrrt-treated ESCs (Fig. 2a, Supplementary Fig. 4a).

Significant changes in premature cleavage/polyadenylation were less common in other introns and lacked the upregulation trend observed for first introns (Fig. 2a). Upregulated CSs in first introns tended to occur relatively close to the 5′ splice site (5′ss) (Fig. 2b). Significantly fewer of these CSs were previously annotated in the polyA_DB3 database^48^ compared to their counterparts located in 3′UTRs of the same genes (30.1% vs 81.4%; Fisher’s exact test *p* = 3.9E-179). However, the incidence of canonical cleavage/PAS AATAAA or its common variant ATTAAA upstream of these two CS categories was virtually indistinguishable (Supplementary Fig. 4b). Hence, Srrt dampens the expression of multiple transcripts terminated at a poorly characterized class of CSs in first introns.

### Srrt blocks cleavage/polyadenylation in first introns

Two possibilities could account for accumulation of prematurely terminated transcripts in response to Srrt knockdown: (1) enhanced pre-mRNA cleavage and polyadenylation at the corresponding intronic positions or (2) increased stability of these relatively short RNA species. The former mechanism should lower the production of full-length mRNA isoforms, while the latter is unlikely to produce this effect. Notably, activation of CSs in first introns strongly correlated with an overall decrease in expression levels of the corresponding genes (Fig. 2c, Supplementary Fig. 4c, Supplementary Data 4) and downregulation of CSs in their 3′UTRs (Supplementary Fig. 4d). There were 284 genes with intronic CS (iCS) upregulated ≥2-fold, FDR < 0.05 and expression level reduced ≥1.5-fold, FDR < 0.05, and an even larger number of genes showing this trend was detected using less stringent cutoffs (Supplementary Data 4). Genes upregulated despite the activation of iCSs were clearly a minority, and the increase in the overall expression levels in this case tended to be due to accumulation of prematurely terminated isoforms (e.g. the *Ttll11* gene in Supplementary Data 4).

RNA-Seq and 3′RNA-Seq coverage plots for individual targets were consistent with our transcriptome-wide analyses (Fig. 2d, Supplementary Fig. 5a). We used the 3′-terminal version of rapid amplification of cDNA ends (3′RACE) to map the regulated iCSs for three genes selected for experimental validation, *Ammecr1*, *Cdyl2*, and *Dcaf6* (Supplementary Fig. 5b). In all three cases, siSrrt increased the RT-qPCR signal upstream of the iCSs and simultaneously reduced the abundance of downstream RNA sequences (Fig. 2e). This corresponded to a ~3–7-fold decrease in the ratio between the full-length and prematurely terminated transcripts, a statistic that we refer to as iCS readthrough efficiency (Supplementary Fig. 5c). A similar decrease in readthrough efficiency was evident when we substituted the siSrrt mixture with any of its three most efficient constituents, siSrrt#1, siSrrt#2, or siSrrt#3 (Supplementary Fig. 6a, b). The three individual siRNAs also caused largely similar to siSrrt effects on the expression of pluripotency and differentiation markers (Supplementary Fig. 6c–e).

To directly test the impact of intronic cleavage/polyadenylation on gene expression, we focused on *Ammecr1*. The overall expression of this biomedically important gene^45^ decreased while the relative abundance of the iCS-terminated species increased during ESC differentiation into neurons, consistent with the *Srrt* downregulation trend (Supplementary Fig. 7a–d). Furthermore, knockdown of the full-length Ammecr1 transcripts induced detectable upregulation of a subset of the siSrrt-induced differentiation markers (Supplementary Fig. 7e, f). *Ammecr1* is encoded on the X chromosome, which also makes it an easy target for reverse genetics in male ESCs.

**Fig. 3:**
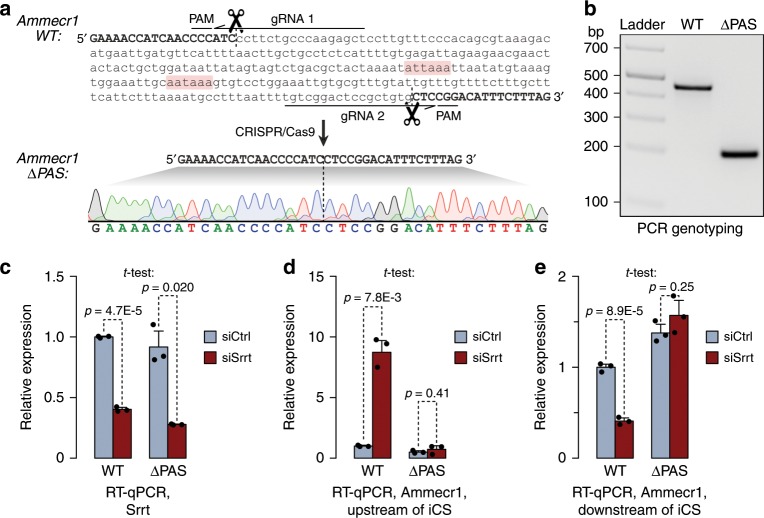
Intronic cleavage/polyadenylation is required for *Ammecr1* regulation by Srrt. **a** Top: Ammecr1 wild-type (WT) intronic sequence regulated in response to Srrt knockdown. Canonical PAS motifs are highlighted in pink. Also shown are positions of CRISPR gRNAs used to generate the *ΔPAS* allele. Sequence deleted in *ΔPAS* is in lowercase. Bottom: Sanger sequence analysis of the ΔPAS Ammecr1 allele. **b** PCR genotyping result comparing WT and ΔPAS ESCs. **c** Passage-matched WT and ΔPAS ESC clones were treated with either siSrrt or siCtrl and the efficiency of Srrt knockdown was analyzed by RT-qPCR 48 h later. Note that Srrt levels decrease to a comparable extent in both genetic backgrounds. **d**, **e** The effect of siSrrt on the expression of Ammecr1 sequences **d** upstream and **e** downstream of the iCS in the *WT* (and the deleted intronic region in the *ΔPAS* allele). Note that deletion of the CS region in ΔPAS cells abolishes **d** siSrrt-induced upregulation of the truncated 5′-proximal transcript and **e** downregulation of the full-length isoform. Data in **c**–**e** were averaged from three experiments ± SD, normalized to the WT/siCtrl samples, and compared by a two-tailed *t*-test. Source data are provided as a Source Data file.

Importantly, when we deleted *Ammecr1* sequence containing two PASs upstream of the strongest Srrt-regulated iCS using CRISPR-Cas9 (Fig. 3a, b), the mutant allele (*ΔPAS*) lost its ability to undergo premature cleavage and reduce its expression output following Srrt knockdown (Fig. 3c–e). Together, these data suggest that Srrt promotes expression of full-length mRNAs by blocking premature cleavage/polyadenylation in first introns.

### iCS repression does not depend on the exosome or small RNAs

Since Srrt has been previously shown to destabilize transcription start site (TSS)-proximal transcripts in an exosome-dependent manner^12^, we compared our 3′RNA-Seq data with results of 3′ end-proximal RNA-Seq (2P-Seq) for mouse ESCs where the exosome complex was inactivated by knockout of its core subunit Exosc3^36^. Metaplot analysis of siSrrt-regulated genes showed a robust accumulation of TSS-proximal RNAs transcribed in the sense but not the antisense direction (Supplementary Fig. 8a). On the other hand, Exosc3 KO increased the abundance of both sense and antisense transcripts in the same genomic regions (Supplementary Fig. 8b), as described previously^36^.

In stark contrast to siSrrt, Exosc3 KO had no detectable effect on the abundance of full-length mRNAs transcribed from Srrt-dependent genes (Supplementary Fig. 8c). Although downregulation of the catalytic exosome subunits Exosc10 and Dis3 by corresponding siRNAs promoted some accumulation of prematurely terminated Ammecr1 RNA (Supplementary Fig. 8d, e), neither these nor an Exosc3-specific siRNA decreased the abundance of full-length Ammecr1 transcripts (Supplementary Fig. 8d, e). Conversely, exosome-specific siRNAs caused more efficient accumulation of TSS-proximal upstream antisense transcripts compared to siSrrt (Supplementary Fig. 8e).

To check the possibility that intronic cleavage/polyadenylation might be controlled through Srrt-stimulated production of small noncoding RNAs^10,14,16^, we turned to published RNA-Seq data for Dicer1/Dicer KO in mouse ESCs with a validated effect on microRNA activity^49^. The gene expression changes induced by Srrt knockdown and Dicer1 KO showed no global correlation (Supplementary Fig. 9a) and the expression of Srrt-regulated genes did not generally change in response to Dicer1 KO (Supplementary Fig. 9b). Moreover, inspection of RNA-Seq coverage profiles for individual Srrt targets showed no evidence for iCS regulation by Dicer (Supplementary Fig. 9c). Thus, neither the exosome nor small RNAs appear to be required for Srrt-mediated repression of intronic cleavage/polyadenylation in mouse ESCs.

### Srrt-mediated repression of iCSs relies on the CBC

**Fig. 4:**
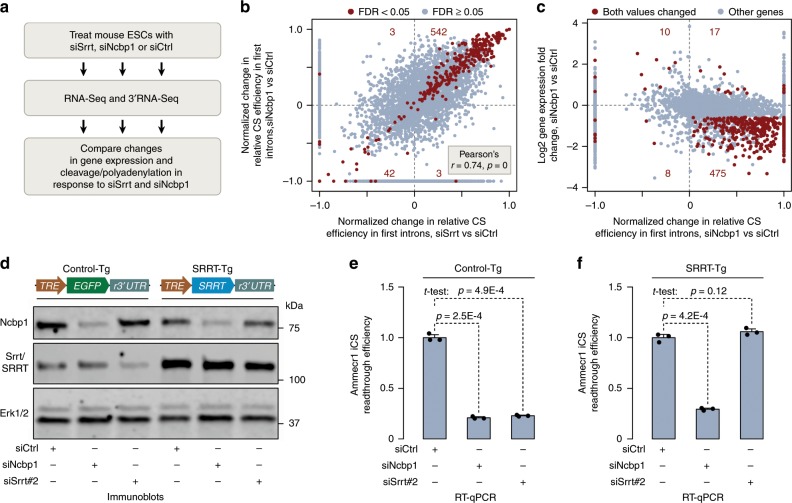
Srrt-mediated repression of iCSs depends on the CBC. **a** Workflow used to compare transcriptome-wide effects of siSrrt and an siRNA targeting Ncbp1. **b** Scatter plot showing a correlation (Pearson’s *r* = 0.74, *p* = 0) between the effects of siSrrt and siNcbp1 on CSs in first introns. Note that most iCSs significantly regulated by both siSrrt and siNcbp1 (FDR < 0.05; red) show an increase in relative efficiency (top right quadrant). **c** Scatter plot showing that, similar to siSrrt (Fig. 2c), siNcbp1-mediated activation of iCSs often coincides with downregulation of corresponding genes. Red, genes with significant changes in relative CS efficiency in first introns (FDR < 0.05) and expression levels (FC ≥ 1.5 and FDR < 0.05). Gray, the rest of the genes. **d** ESCs containing a human SRRT transgene (SRRT-Tg; *TRE-SRRT-r3*′*UTR*) or a control expression cassette (Control-Tg; *TRE-EGFP-r3*′*UTR*) were pre-treated with 2 µg/ml Dox for 24 h and transfected with siCtrl, siNcbp1, or siSrrt. Expression levels of the Ncbp1 and Srrt proteins were analyzed by immunoblotting 48 h later. Note that, compared to siCtrl, siNcbp1 and siSrrt reduce the abundance of the corresponding proteins in both transgenic backgrounds. However, the combined Srrt/SRRT expression in the SRRT-Tg/siSrrt sample still exceeds the Srrt levels in Control-Tg/siCtrl. Erk1/2, lane loading control. *TRE*, Dox-inducible promoter; *r3*′*UTR*, recombinant 3′UTR from SV40 virus. For quantification of this experiment see Supplementary Fig. 10d. **e**, **f** RT-qPCR analysis showing that **e** both siSrrt and siNcbp1 decrease transcriptional readthrough of iCS in the *Ammecr1* gene in the Control-Tg background. **f** Recombinant SRRT rescues the effect of siSrrt but not siNcbp1 in the SRRT-Tg cells suggesting that Ncbp1 is essential for Srrt-mediated repression of iCSs. Data in **e**, **f** were averaged from three experiments ± SD and compared by a two-tailed *t*-test. Source data are provided as a Source Data file.

To examine possible contribution of the CBC to the Srrt-dependent antitermination activity, we knocked down Ncbp1 in mouse ESCs and compared the effect of this treatment with that induced by siSrrt (Fig. 4a). RNA-Seq and 3′RNA-Seq analyses revealed a noticeable correlation between the siNcbp1- and the siSrrt-treated samples in terms of overall gene expression changes and activation of CSs in first introns (Fig. 4b, c, Supplementary Fig. 10a–c).

To test if Srrt and Ncbp1 functioned in the same pathway, we generated an ESC line containing a doxycycline (Dox)-inducible human SRRT transgene (SRRT-Tg) resistant to mouse-specific siSrrt (Fig. 4d, Supplementary Fig. 10d). Importantly, SRRT-Tg was sufficient to rescue termination of Ammecr1 transcripts in the first intron induced by siSrrt but not by siNcbp1 (Fig. 4e, f). In line with this functional interaction between the two proteins and published data for their human counterparts^11,12^, Srrt and Ncbp1 interacted physically in mouse ESCs in a nucleic acid-independent manner (Supplementary Fig. 10e). RNA immunoprecipitation (RIP) with Ncbp1-specific antibodies showed that siSrrt did not alter the ability of Ncbp1 to interact with (pre-)mRNAs (Supplementary Fig. 10f), suggesting that Ncbp1 might be required for recruiting Srrt to its targets but not the other way around.

We concluded that the ability of Srrt to repress cleavage/polyadenylation in first introns depends on its interaction with the CBC.

### Srrt facilitates U1-binding upstream of regulated iCSs

**Fig. 5:**
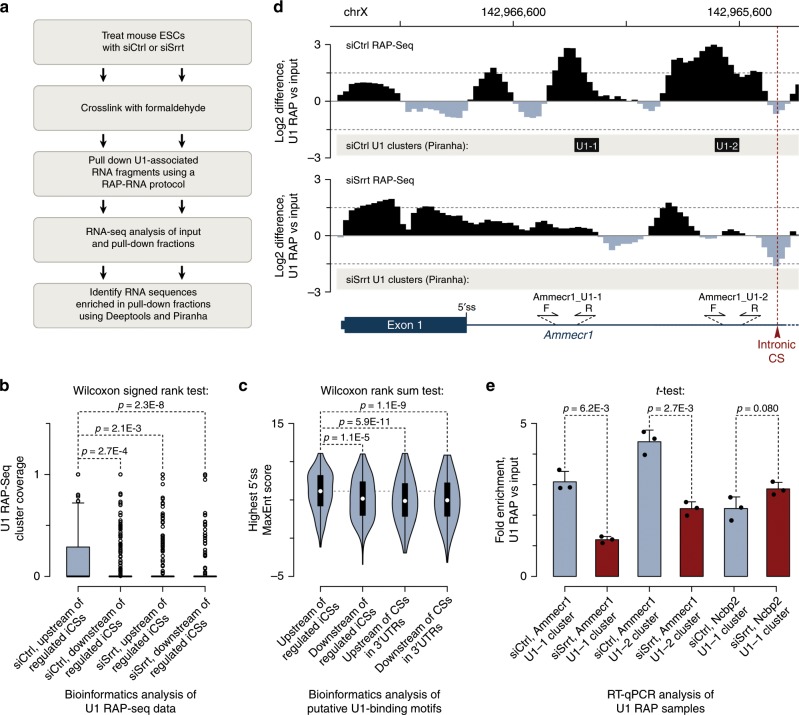
Srrt stimulates U1-binding upstream of CSs in first introns. **a** Outline of the U1 RAP-Seq experiment. **b** Boxplot of U1 RAP-Seq cluster coverage showing stronger binding of U1 snRNP in a 250-nt window upstream of Srrt-regulated iCSs than in a similarly sized window downstream of these sites in siCtrl-treated samples. Note that U1-binding efficiency is diminished following Srrt knockdown. *P* values were calculated using a two-tailed Wilcoxon signed rank test. The box bounds represent the first and the third quartiles and the thick black lines at the bottom of the boxes show the medians. Since the distributions are skewed towards 0, only the top whisker is evident, extending to 1.5× of the range between the third and the first quartiles (interquartile range). Open circles, outliers. **c** Consistent with the data in **b**, the 250-nt window upstream of Srrt-repressed CSs tends to contain stronger putative U1-binding motifs (measured as the maximum 5′ss MaxEnt value) than the 250-nt downstream window or similarly sized windows abutting CSs in the corresponding 3′UTRs. *P* values were calculated using a two-tailed Wilcoxon rank sum test. Violin plot outlines show kernel density estimates of probability densities; open circles, the medians; and bounds of the black boxes, the first and the third quartiles. Whiskers extend from the first and the third quartile to the lowest and highest data points or, if there are outliers, 1.5× of the interquartile range. **b**, **c** iCSs were considered regulated if they were upregulated ≥2-fold, FDR < 0.05 and their host gene was downregulated ≥1.5-fold, FDR < 0.05 in response to siSrrt. **d** Input-normalized RAP-Seq coverage profile and Piranha clusters (U1-1 and U1-2) showing strong interaction of U1 snRNP with at least two intronic positions preceding the Srrt-repressed CS in the *Ammecr1* gene in the siCtrl- but not siSrrt-treated ESCs. Sequences enriched in RAP-Seq vs input are shown in black and those depleted are in gray. Primers used in the RT-qPCR validation experiment in **e** are shown at the bottom. **e** RT-qPCR validation of RAP-Seq results using primer pairs matching U1 Piranha clusters in **b** and Supplementary Fig. 11e. Note that input-normalized signals are significantly higher in siCtrl U1 RAP samples than in their siSrrt-treated counterparts for the two regulated Ammecr1 clusters but not for a control cluster in the Ncbp2 pre-mRNA. Data were averaged from three experiments ± SD and compared by a two-tailed *t*-test. Source data are provided as a Source Data file.

CBC can promote recruitment of U1 to cap-proximal introns, and this snRNP can in turn antagonize cleavage/polyadenylation via telescripting^18,31^. To assess possible contribution of these mechanisms, we mapped U1-binding sites in formaldehyde-crosslinked ESCs using RNA antisense purification-sequencing (RAP-Seq; ref. ^50^; Fig. 5a). We ascertained that the U1 pull-down procedure worked successfully by monitoring enrichment of U1 snRNA precursors and depletion of the 45S ribosomal RNA (Supplementary Fig. 11a). Reflecting the known U1 interaction preferences, input-normalized RAP-Seq reads showed a detectable bias towards the 5′ end of all introns and first introns containing Srrt-repressed iCSs (Supplementary Fig. 11b, c).

Although the siCtrl- and the siSrrt-treated ESCs showed generally similar U1-binding profiles (Supplementary Fig. 11b, c), we noticed a discernable U1 peak upstream of the Srrt-regulated iCSs in the siCtrl but not the siSrrt sample (Supplementary Fig. 11d). Supporting this observation, the incidence of U1 clusters deduced using a previously described approach^51^ was significantly higher in a 250-nt window upstream of Srrt-repressed iCSs than in a similarly sized downstream window in the siCtrl-treated cells (Fig. 5b). This was consistent with enrichment of relatively strong U1-binding motifs upstream of iCSs compared to corresponding downstream positions and 250-nt windows adjoining CSs in 3′UTRs of the same genes (Fig. 5c). Importantly, Srrt knockdown led to a significant drop in U1 cluster coverage upstream of the regulated iCSs (Fig. 5b).

The above effects were also detectable for individual Srrt targets. For example, two prominent U1 RAP-Seq peaks between the 5′ss and the strongest Srrt-repressed CSs in the first intron of the *Ammecr1* gene were significantly enriched over the input in the siCtrl- but not the siSrrt-treated samples (Fig. 5d). RT-qPCR analyses of the pull-down and the input fractions confirmed that U1 binding to the corresponding intronic positions was significantly reduced by Srrt knockdown (Fig. 5e). In contrast, U1 occupancy in the first intron of *Ncbp2*, a control gene not regulated by Srrt, showed no significant difference between the siCtrl and siSrrt samples (Fig. 5e, Supplementary Fig. 11e).

The siSrrt effect on U1 recruitment was not due to major changes in U1 snRNA steady-state levels or its processing efficiency (Supplementary Fig. 12a, b). The levels of the U1 snRNP proteins Snrpa/U1-A and Snrp70/U1-70K were also unaffected (Supplementary Fig. 12c, d). Furthermore, we compared our 3′RNA-Seq data for siSrrt-treated samples with a similar analysis published for mouse ESCs where U1 was inactivated by an antisense morpholino oligonucleotide (AMO)^36^. Although both treatments promoted premature cleavage/polyadenylation in first introns, inactivation of U1 clearly differed from Srrt knockdown by additionally inducing this effect in non-first introns on a transcriptome-wide scale (Supplementary Fig. 12e, f).

These data suggest that Srrt facilitates U1 recruitment upstream of regulated CSs in first introns rather than substantially altering overall activity of this snRNP in mouse ESCs.

### Srrt-recruited U1 can promote telescripting

**Fig. 6:**
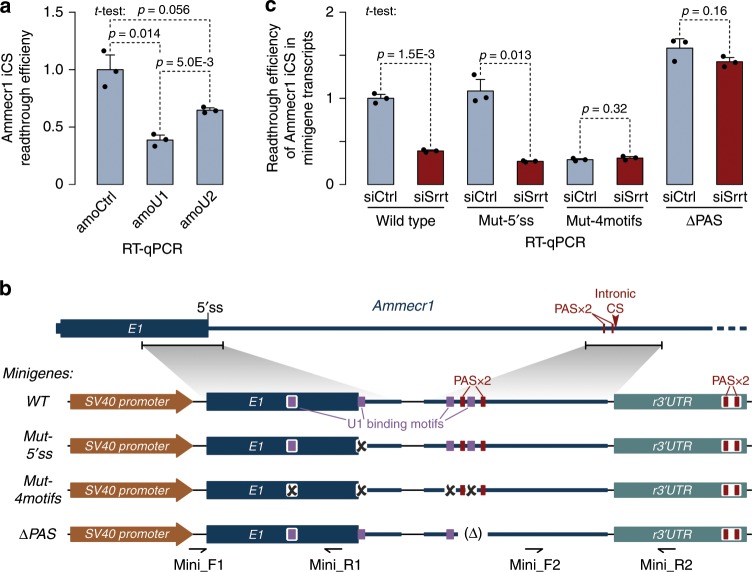
Srrt-mediated readthrough of Ammecr1 iCS depends on telescripting. **a** Mouse ESCs were nucleofected with a control morpholino oligonucleotide (amoCtrl) or antisense morpholinos targeting either U1 or U2 snRNA (amoU1 and amoU2) for 8 h and the effect of these treatments on the iCS readthrough was analyzed using RT-qPCR. Note that amoU1 leads to a robust decrease in the CS readthrough efficiency compared to amoCtrl and amoU2. **b** Ammecr1-based minigene constructs used in telescripting assays in **c**. *PSV40*, SV40 enhancer, and early promoter; *E1*, the first exon of *Ammecr1* gene; *r3*′*UTR*, recombinant 3′UTR from SV40 virus. **c** ESCs transiently transfected with wild type (*WT*) or mutant (*Mut-5*′*ss*, *Mut-4motifs*, or *ΔPAS*) minigenes from **b** were treated with either siCtrl or siSrrt and the Ammecr1 intronic CS efficiency was assayed as a ratio between downstream [mini_F2/mini_R2 primers in **b**] and upstream RT-qPCR signals [mini_F1/mini_R1 primers in **b**]. Note that Srrt stimulates CS readthrough in the WT minigene, similar to its effect on the endogenously encoded *Ammecr1*. Mutation of a single U1-binding motif corresponding to the 5′ss at the beginning of the first intron (*Mut-5*′*ss*) does not alter the minigene response to Srrt knockdown; however, mutation of 5′ss and three additional sites potentially interacting with U1 (*Mut-4motifs*) results in a constitutive cleavage/polyadenylation phenotype. Conversely, deletion of the two PAS motifs (*ΔPAS*) leads to constitutive readthrough. Data on **a** and **c** were averaged from three experiments ± SD and compared by a two-tailed *t*-test. Readthrough efficiencies in the amoCtrl and *WT*/siCtrl samples, respectively, were set to 1. Source data are provided as a Source Data file.

As a direct test of the U1 effect on iCSs, we treated ESCs with a U1-specific AMO (amoU1; Fig. 6a). This enhanced the efficiency of premature cleavage/polyadenylation in the first intron of Ammecr1 pre-mRNA compared to samples treated with a non-targeting control (amoCtrl) or an antisense morpholino against another spliceosomal snRNA, U2 (amoU2). The noticeably stronger effect of amoU1 than that of amoU2 suggested that Srrt-stimulated recruitment of U1 snRNP could inhibit iCSs through telescripting rather than the spliceosome assembly pathway.

To test this hypothesis, we prepared a minigene construct by fusing the exon 1-intron 1 junction and the Srrt-regulated iCS region of the *Ammecr1* gene with a recombinant 3′UTR containing a constitutive CS (Fig. 6b). Since it lacked a functional 3′ss, this cassette allowed us to assay telescripting in the absence of pre-mRNA splicing. The minigene was expressed in ESCs pre-treated with siSrrt or siControl, and the use of the Ammecr1 iCS was analyzed by RT-qPCR (Fig. 6c). Recapitulating the behavior of endogenous Ammecr1 pre-mRNAs, minigene-derived transcripts showed more efficient iCS readthrough in the siCtrl than in the siSrrt samples (Fig. 6c).

Mutation of the 5′ss, i.e. the site where U1 binds to initiate splicing of endogenous Ammecr1 transcripts, had no detectable effect on the minigene response to siSrrt (Fig. 6c). However, when we mutated three additional positions predicted to interact with U1, the minigene was terminated at the iCS regardless of the Srrt expression levels (Fig. 6c). On the other hand, deletion of the PAS hexamers (ΔPAS) preceding the iCS led to a constitutive readthrough phenotype (Fig. 6c).

These results confirm that Srrt can block intronic cleavage/polyadenylation through a U1-dependent telescripting mechanism.

### Many iCSs emerged through retrotransposition

**Fig. 7:**
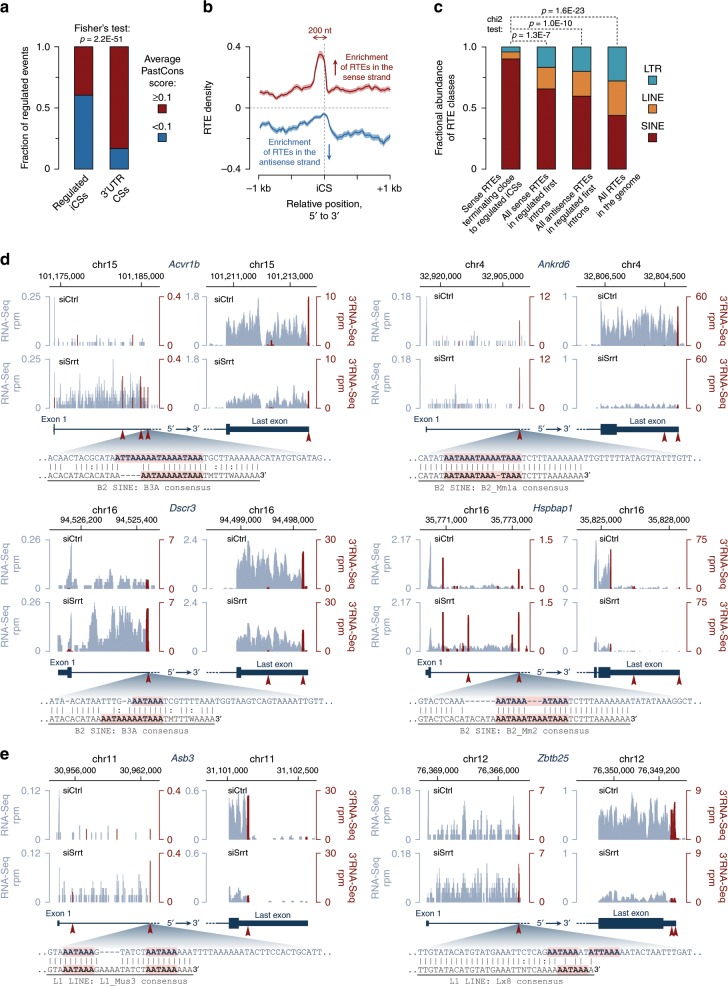
Regulated iCSs often appear as a result of retrotransposition. **a** Fisher’s exact test showing that Srrt-regulated iCSs are less frequently conserved across placental mammals as compared to their 3′UTR counterparts. **b** Metaplots showing strong enrichment of retrotransposable elements (RTEs) in sense orientation immediately upstream of regulated iCSs (red line ± SEM) and their relative depletion in the CS-proximal region on the antisense strand (blue line ± SEM). Note that the antisense RTE density values were multiplied by −1. **c** iCS-associated RTEs (sense-strand RTEs terminating in ±50 nt vicinity of regulated iCSs) are enriched for SINEs as compared to the overall incidence of these elements in regulated first introns or the entire genome. **a**–**c** iCSs were considered regulated if they were upregulated in response to siSrrt ≥2-fold, FDR < 0.05 and their host gene was downregulated ≥1.5-fold, FDR < 0.05. **d**, **e** Examples of Srrt-dependent genes with iCSs matching 3′ ends of sense-strand **d** SINEs or **e** LINEs. RNA-Seq coverage plots are shown in gray and 3′RNA-Seq data are in red. Similar to the genes with conserved iCSs in Fig. 2d, upregulation of RTE-associated iCSs in response to siSrrt leads to a pronounced decrease in the RNA-Seq and 3′RNA-Seq signals in corresponding 3′UTRs. Red arrowheads, CSs preceded by AATAAA or ATTAAA hexamers. Pairwise alignments between regulated CSs and corresponding RTE consensus sequences are shown at the bottom of each panel with invariant positions marked by vertical bars and degenerate matches and base transitions indicated by colons. Canonical PAS hexamers are highlighted in pink.

Our data so far suggested that productive transcription of a large subset of genes active in ESCs depends on Srrt abundance. To understand evolutionary mechanisms underlying this regulation, we examined interspecies conservation scores^52^ for 50 nt windows bounded by 40 nt upstream and 10 nt downstream of Srrt-regulated iCSs (Fig. 7a). A fraction of these sequences (39.6%) showed detectable conservation (average PhastCons score ≥ 0.1). This category included *Ammecr1*, *Cdyl2*, and *Dcaf6*, which had their iCS-associated PAS hexamers present in several mammalian species (Supplementary Fig. 13).

A majority of the Srrt-regulated sequences (60.4%) were conserved poorly or not at all (average PhastCons score < 0.1). Since RTEs provide an important source of interspecies diversity^53,54^, we wondered if mouse/rodent-specific iCSs could appear as a result of relatively recent retrotransposition events. Strikingly, an RTE density plot revealed a prominent peak of these elements integrated in the sense orientation immediately upstream of the Srrt-repressed iCSs (Fig. 7b). Conversely, antisense RTE sequences were depleted in this region (Fig. 7b).

The iCS-associated sense-strand peak was ~200 nt wide suggesting that it could be dominated by relatively short RTEs (Fig. 7b). Indeed, most of the sense-strand RTEs that terminated around an iCS (±50 nt) belonged to the group of short interspersed nuclear elements (SINEs), although a few long interspersed nuclear elements (LINEs) and long terminal repeats (LTRs) were also detected (Fig. 7c)^53,54^. Members of the B2 SINE family were especially common at this position (Supplementary Fig. 14a), consistent with the presence of canonical PASs in their consensus sequence^55^. Overall, 31.2% of all regulated iCSs were associated with 3′ ends of sense-strand RTEs.

iCS-associated B2 SINEs were found for example in genes encoding activin receptor Acvr1b (see also Fig. 1g), WNT pathway modulator Ankrd6/Diversin, Down Syndrome critical region protein Dscr3, and heat-shock protein-associated factor Hspbap1 (Fig. 7d, Supplementary Data 4; https://www.genecards.org). Genes with iCSs occurring at the end of a LINE repeat included those encoding ankyrin repeat and SOCS box protein Asb3 and a component of a regulatory complex interacting with unmethylated DNA in ESCs, Zbtb25 (Fig. 7e, Supplementary Data 4; https://www.genecards.org). In many cases, PAS hexamers preceding iCSs matched corresponding elements in the parental RTEs (Fig. 7d, e).

**Fig. 8:**
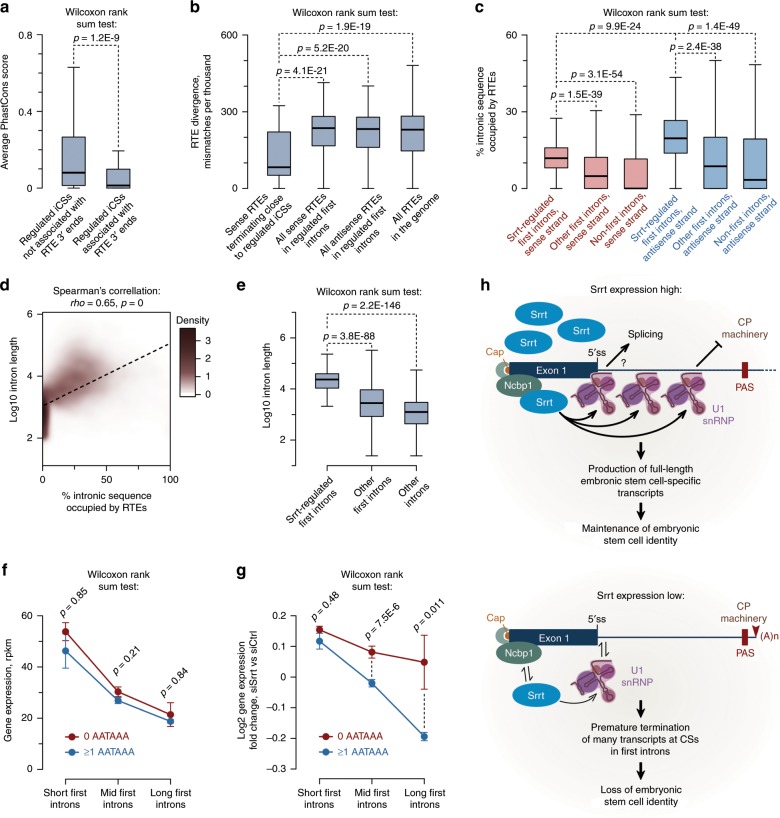
Recurrent retrotransposition may increase gene dependence on Srrt. **a** Regulated iCSs associated with 3′ ends of sense-strand RTEs show significantly lower evolutionary conservation (PhastCons) score than other regulated iCSs. **b** Sense-strand RTEs terminated in iCS vicinity are typically less divergent from the corresponding master copies than control groups. **c** The overall RTE density is significantly higher in Srrt-regulated first introns than in non-regulated first or non-first introns. Also note a strong bias towards antisense orientation of RTEs in all groups of introns. **d** Length of first introns positively correlates with the percent of sequence occupied by RTEs on both strands. Dashed line, linear regression. **e** Consistent with their higher RTE load, the length of first introns in Srrt-dependent genes tends to exceed that of non-regulated or non-first introns. **a**–**c**, **e** iCSs were considered regulated if they were upregulated in response to siSrrt ≥2-fold, FDR < 0.05, and their host gene was downregulated ≥1.5-fold, FDR < 0.05. In **a**–**c** and **e**, box bounds, the first and the third quartiles; thick black lines, the medians. Whiskers extend from the first and the third quartile to the lowest and highest data points or, if there are outliers, 1.5× of the interquartile range. Outliers are not shown. **f** Gene expression in ESCs shows a negative relationship with the length of the first intron even in the presence of normal amounts of Srrt. Shown are mean expression values ± SEM in siCtrl-treated ESCs for genes with short (shorter than the 1/3 quantile; i.e. <1524 nt), midsize (i.e. longer than or equal to the 1/3 quantile but shorter than the 2/3 quantile; i.e. ≥1524 and <7251 nt), and long first introns (longer than or equal to the 2/3 quantile; i.e. ≥7251 nt). Note that genes with AATAAA(s) in the first intron are expressed at levels statistically indistinguishable from their AATAAA-free counterparts. **g** Srrt knockdown leads to preferential downregulation of genes with long first introns containing at least one AATAAA hexamer. **h** Naturally high levels of Srrt help ESCs to maintain their gene expression program through a transcription antitermination mechanism.

iCSs occurring at the 3′ end of sense-strand RTEs were significantly less conserved than the rest of the iCSs (Fig. 8a), suggesting that the corresponding RTE sequences might be a result of relatively recent jumps. Indeed, the iCS-associated repeats were less divergent from the master copies, as compared to control groups comprising all sense or antisense repeats from first introns or the entire collection of repeats found in the mouse genome (Fig. 8b).

Regardless of the RTE association status of their iCSs, all Srrt-regulated first introns showed a significantly higher density of RTE-derived sequences compared to non-regulated first or non-first introns (Fig. 8c, Supplementary Fig. 14b). We also observed a strong bias towards antisense orientation of RTEs in all groups of introns (Fig. 8c), suggesting that sense-oriented RTEs might be more disruptive and therefore subject to stronger purifying selection than their antisense counterparts.

We concluded that, in addition to controlling evolutionarily conserved events, Srrt might repress deleterious iCSs appearing as a result of retrotransposition.

### Srrt target genes tend to have long RTE-rich first introns

Telescripting is known to be critical for production of long transcripts^33^. Interestingly, we detected a genome-wide correlation between the RTE density and the overall size of first introns (Fig. 8d). In line with their increased RTE load, first introns of Srrt-dependent genes tended to be significantly longer compared to control groups (Fig. 8e). Of note, Srrt-regulated and non-regulated first introns were indistinguishable based on their 5′ss strength (Supplementary Fig. 14c).

To find out if the length of first introns might be a good predictor of the Srrt dependence, we plotted average rpkm values in control-treated ESCs for genes separated into three equally sized groups according to the length of their first intron (short, mid, and long; Fig. 8f). Genes with longer first introns tended to be expressed at lower levels in ESCs even in the presence of normal amounts of Srrt. The presence of one or more AATAAA hexamers in the first intron was associated with somewhat reduced average expression in each category, but this effect was not statistically significant (Fig. 8f). Notably, the length of the first intron showed a strong positive association with the ability of AATAAA to dampen gene expression in response to Srrt knockdown (Fig. 8g).

Thus, recurrent RTE jumps may sharpen the dependence of gene expression on Srrt by increasing the length of first introns.

## Discussion

Our study uncovers a global antitermination mechanism responsible for productive expression of multiple genes in pluripotent stem cells (Fig. 8h). This mechanism relies on the ability of Srrt to associate with the CBC and block premature cleavage/polyadenylation of pre-mRNAs in first introns by promoting recruitment of U1 snRNP to cap-proximal sequences. We show that, at least in the case of the disease-associated gene *Ammecr1*, Srrt-augmented U1 binding can promote transcriptional readthrough of a downstream iCS as a result of telescripting.

Three lines of evidence argue that Srrt is an important regulator of ESC identity. (1) Srrt is substantially more abundant in ESCs than in other cell types including actively proliferating NSCs (Fig. 1b, Supplementary Fig. 1a, b). (2) Normal expression of hundreds of iCS-containing genes active in ESCs relies on the naturally high levels of Srrt (Fig. 2c, Supplementary Fig. 4d and Supplementary Data 4). (3) Srrt downregulation in ESCs to levels considered physiological in other cell types induces several differentiation-specific changes (Fig. 1b–g and Supplementary Figs. 1 and 2a–c). It is possible that the latter effect depends, at least in part, on reduced expression of a subset of the iCS genes. Indeed, knockdown of *Ammecr1* leads to statistically significant upregulation of some differentiation markers induced in response to Srrt-specific siRNAs (Supplementary Fig. 7f). Further research will be required to understand molecular functions of the Ammecr1 protein and identify other Srrt targets that may contribute to the ESC differentiation phenotype.

The role of Srrt in ESCs appears to be distinct from its function as a transcriptional activator of *Sox2* gene in NSCs^24^. Sox2 mRNA levels did not change in our siSrrt-treated samples implying that other mechanisms must ensure robust expression of this important transcription factor in ESCs. This may be achieved through cross-activation of *Sox2* by Pou5f1, Nanog, or other transcriptional regulators present in ESCs but not NSCs^37–39^. Alternatively, it is possible that the residual amount of Srrt protein in siSrrt-treated ESCs (Fig. 1c) is sufficient for promoting *Sox2* transcription but not for blocking iCSs. Consistent with a possible difference in quantitative requirements of the two mechanisms, Srrt is ~3 times more abundant in ESCs than in NSCs cultured in vitro (Fig. 1b).

Our data support the emerging view that, in addition to their reliance on transcription factors, pluripotent stem cells depend on adequate expression patterns of a number of RNA-associated proteins. These include for example pre-mRNA splicing regulators identified in recent studies^56–59^. It is likely that further quantitative analyses of expression changes triggered by ESC differentiation or transition of differentiated cells to induced pluripotency will uncover additional factors altering RNA processing and tuning the way it communicates with transcription.

Mounting evidence suggests that U1 snRNP-dependent readthrough of premature CSs is a widespread mechanism facilitating efficient transcription of long mammalian genes^31,33^. Furthermore, many Pol II promoters are inherently bidirectional and the preferred direction for productive elongation appears to be selected based on the ability of promoter-proximal RNA sequences to recruit U1 snRNPs and limit the effect of premature cleavage/polyadenylation^34–36^. Interestingly, the efficiency of telescripting can be modulated by dynamic interactions between the U1 snRNP and nascent pre-mRNA pools, linking rapid transcriptional activation in cells responding to external cues with corresponding changes in alternative cleavage/polyadenylation patterns^32^.

We extend this line of research by showing that the ability of U1 to inhibit cryptic CSs can be tuned depending on the cell type and the 5′ to 3′ position of regulated sequences. This regulation logic is conceptually similar to prokaryotic antitermination used for example by bacteriophage λ to switch between immediate and delayed early stages of its gene expression program^60^. Despite fundamental mechanistic differences both systems rely on elevated expression of key RNA-associated factors, Srrt in ESCs and the N protein in λ, to repress transcription termination signals.

We cannot currently rule out that, in a subset of genes, Srrt-recruited U1 may antagonize intronic cleavage/polyadenylation through kinetic competition with splicing, instead of or in addition to telescripting. Supporting possible involvement of Srrt in splicing, some of its targets not regulated at the level of mRNA abundance appear to retain first introns in siSrrt-treated ESCs (yellow line in Supplementary Fig. 3d). Moreover, Srrt is known to control splicing decisions in plants^61,62^. What might determine the choice between telescripting- and splicing-dependent mechanisms on a transcriptome-wide scale is an interesting question for future studies.

It will be also important to understand how different molecular activities of Srrt are balanced depending on the cell type and RNA target identity. Especially intriguing is the ability of Srrt to promote 3′-terminal processing/termination in some cases^11,12,14,63^ while antagonizing it in a transcriptome-wide manner in mouse ESCs (Fig. 2c, Supplementary Fig. 4d and Supplementary Data 4). We envisage at least two non-mutually exclusive explanations. (1) Srrt may block cleavage/polyadenylation only in the presence of sufficiently strong U1-binding motifs between the 5′-terminal cap and the iCS. In addition to promoting telescripting, U1 recruited to these positions might potentially compete with cleavage/polyadenylation machinery for overlapping interaction sites in the Srrt protein. (2) Alternatively, ESCs may express yet-to-be identified Srrt-associated factors overriding the ability of this multipurpose adaptor to stimulate cleavage/polyadenylation or/and strengthening its contacts with U1.

Several Srrt-regulated iCSs appear to be conserved in evolution (Fig. 7a, Supplementary Fig. 13), pointing at their potential adaptive value. For example, such intronic elements may limit the abundance of ESC-enriched transcripts in other cell types. Supporting this possibility, the progressive decline in *Ammecr1* expression during neuronal differentiation correlates positively with the Srrt downregulation trend and negatively with an increase in the relative abundance of iCS-terminated Ammecr1 transcripts (Supplementary Fig. 7a–d). However, most iCSs lack detectable interspecies conservation and many of them are associated with relatively recent retrotransposition events (Figs. 7 and 8a, b).

What could be the role of Srrt in this context? Interestingly, Srrt-regulated first introns have a higher RTE load compared to non-regulated first and non-first introns (Fig. 8c, Supplementary Fig. 14b). This might reflect possible integration bias of RTEs to open chromatin, making first introns in genes transcriptionally active at the preimplantation stage especially vulnerable to recurrent and potentially heritable retrotransposition^64–66^. Accumulation of RTEs in this region would in turn dampen gene expression by introducing PASs/iCSs directly (Fig. 7) or making the acquisition of new PAS-like mutations more likely due to an increase in intron length (Fig. 8c–g, Supplementary Fig. 14b).

We propose that the natural over-expression of Srrt helps ESCs to alleviate potentially damaging consequences of this genome-wide effect. The largely negative impact of RTEs on individual fitness is often discussed in conjunction with their role as an important source of evolutionary innovation^53,54,67–70^. Hence, an intriguing possibility that should be investigated in the future is that, besides protecting the transcriptome, Srrt may also function as a genetic capacitor allowing initially deleterious events to be repurposed for building new regulation modules.

## Methods

### Cell culture techniques

A2lox mouse ESCs^71^ were cultured in a humidified incubator at 37 °C, 5% CO_2_, in plates or dishes coated with gelatin (Millipore, cat# ES-006-B) in 2i medium^37^ containing a 1:1 mixture of Neurobasal (Thermo Fisher Scientific, cat# 21103049) and DMEM/F12 (Sigma, cat# D6421) media supplemented with 100 units/ml PenStrep (Thermo Fisher Scientific, cat# 15140122), 1 μM PD03259010 (Cambridge Bioscience, cat# SM26-2), 3 μM CHIR99021 (Cambridge Bioscience, cat# SM13-1), 0.5 mM l-glutamine (Thermo Fisher Scientific, cat# 25030024), 0.1 mM β-mercaptoethanol (Sigma, cat# M3148), 1000 units/ml ESGRO LIF (Millipore, cat# ESG1107), 0.5× B-27 supplement without vitamin A (Thermo Fisher Scientific, cat# 12587010) and 0.5× N2 supplement. N2 100× stock was prepared using DMEM/F12 medium as a base and contained 5 mg/ml BSA (Thermo Fisher Scientific, 15260037), 2 µg/ml progesterone (Sigma, P8783-1G), 1.6 mg/ml putrescine (Sigma, P5780-5G), 3 µM sodium selenite solution (Sigma, S5261-10G), 10 mg/ml apo-transferrin (Sigma, T1147-100MG), and 1 mg/ml insulin (Sigma, I0516-5ML) and stored in single-use aliquots at −80 °C.

Cells were typically passaged every 2–3 days by treating the cultures with 0.05% Trypsin-EDTA (Thermo Fisher Scientific, cat#15400054) for 8–10 min at 37 °C. After quenching trypsin with FBS (Thermo Fisher Scientific, cat# SH30070.03E), cells were washed once with neurobasal medium and plated at a 1:6 dilution.

For RNA interference (RNAi) experiments, 2 × 10^5^ cells were seeded in 1 ml of 2i medium per gelatinized well of a 12-well and immediately transfected with 50 pmol of an appropriate siRNA (Horizon Discovery; see Supplementary Data 5 for details) premixed with 3 µl of Lipofectamine 2000 (Thermo Fisher Scientific, cat# 11668019) and 100 µl of Opti-MEM I (Thermo Fisher Scientific, cat# 31985070), as recommended. The cultures were then incubated for 48 h without changing the medium. In minigene experiments, cells pre-treated with siRNAs for 24 h were transfected with 500 ng of minigene plasmid mixed with 2 µl of Lipofectamine 2000 and 100 µl of Opti-MEM I and incubated for another 24 h prior to RNA extraction.

Stable knock-in lines were generated as follows. A2lox cells were pre-treated overnight with 1 μg/ml doxycycline (Dox; Sigma, cat# D9891-1G) to activate Cre expression, trypsinized, and then transfected in suspension with 1 μg of an appropriate p2Lox-based plasmid mixed with 3 µl of Lipofectamine 2000 and 100 µl of Opti-MEM I in 4 ml of 2i medium in 6 cm bacterial dishes at 0.75–1 × 10^5^ cells/ml. Cells were collected 2 h post-transfection and serially diluted in 2i medium prior to re-plating in six-well format. On the next day, 350 μg/ml of geneticin/G418 (Sigma, cat# 10131019) was added and the incubation was continued for an additional 8–12 days with regular medium changes to allow geneticin-resistant cells to form colonies. These were picked, expanded, and analyzed for inducible expression of transgenic sequences using reverse transcriptase-quantitative PCR (RT-qPCR) and/or immunoblotting.

Genomic deletions were generated in A2Lox cells containing a Dox-inducible Cas9 transgene. Cells were pre-treated with 1 μg/ml Dox overnight, transfected with a mixture containing two synthetic EditR gRNAs flanking the deletion region (50 pmol each; Horizon Discovery; see Supplementary Data 5) or two EditR Non-targeting control gRNAs (50 pmol each; Horizon Discovery, cat# U-007501-01-05 and U-007501-01-05) and 100 pmol of synthetic EditR tracrRNA (Horizon Discovery, cat# U-002005-05) at 1–2 × 10^5^ cells per well of a 12-well plate using conditions described for RNAi experiments. Cells were trypsinized 24 h post-transfection, FBS-quenched, passed through Falcon 40 μm cell strainers (Corning, cat# 352340) to obtain a single-cell suspension, and serially diluted in 2i medium prior to re-plating in six-well format. The cultures were then maintained for 8–12 days with regular medium changes and colonies originating from individual cells were picked, expanded, and their genomic DNA was analyzed for the presence of desired deletion using PCR genotyping (see below).

For AMO delivery, 2 × 10^6^ ESCs were electroporated in the presence 7.5 μM of U1-specific, U2-specific, or a scrambled AMO (Gene Tools, LLC; see Supplementary Data 5) in Amaxa Nucleofector II (Lonza) using ESC-specific program A-23 and Mouse Embryonic Stem Cell Nucleofector Kit (Lonza, cat# VPH-1001) as recommended. Nucleofected cells were maintained in 2i medium in a single well of a six-well plate for 8 h prior to RNA purification and RT-qPCR analysis.

### Pluripotency/differentiation assays

To assess gene knockdown effects on ESC pluripotency/differentiation status, siRNA-transfected cells were incubated in 2i medium supplemented with 2% FBS for 48 h and stained using an alkaline phosphatase detection kit (Millipore, cat# SCR004) as recommended. In colony formation assays, siRNA-transfected cells were trypsinized 24 h post-transfection, quenched with FBS, passed through Falcon 40 μm cell strainers, and plated at 1000 cells per well of a six-well plate in 2i medium supplemented with 2% FBS. Seven days post plating cell colonies were stained for alkaline phosphatase, imaged, and analyzed using ImageJ (https://imagej.nih.gov/ij/; see Supplementary Data 5 for further information on the computer software used in this study).

For flow cytometry, ESCs transfected with siRNAs in a 12-well plate format were incubated in 2i medium for 48 h, dissociated using Accutase (Thermo Fisher Scientific, cat# A1110501), washed with 1× PBS, pH 7.4 (Thermo Fischer Scientific, cat# 10010023), and resuspended in 100 μl of FACS buffer containing 1× PBS, 2 mM EDTA, and 3% FBS. Cells were then stained for ESC surface markers using an APC-conjugated anti-Pecam1/CD31 antibody (Thermo Fisher Scientific, cat# 17-0311-80, 0.5 μg per test) and an Alexa Fluor 488-conjugated anti-SSEA1 antibody (Thermo Fisher Scientific, cat# 53-8813-41, 0.125 μg per test) for 1 h on ice, washed twice with 300 μl of the FACS buffer, and passed through Falcon 40 μm cell strainers to obtain single-cell suspensions. Samples were supplemented with 0.2 μg/ml DAPI ~10 min prior to flow cytometry to label membrane-compromised cells. Cells were then analyzed using a BD FACSCanto™ II cytometer equipped with 405, 488, and 633 nm lasers. The FCS files were analyzed using the flowCore and the flowViz packages (https://www.bioconductor.org/packages/release/bioc/html/flowCore.html; https://www.bioconductor.org/packages/release/bioc/html/flowViz.html). The following gating strategy was applied to select individual living (DAPI-negative) cells:

rg<-rectangleGate(filterId = “myFilter”, “FSC.A” = c(60000, 140000), “SSC.A” = c(20000, 130000), “SSC.W” = c(80000, 160000), “DAPI.A” = c(−100, 5000))

The Pecam1 (APC) and SSEA1 (Alexa Fluor 488) signals were then measured in cells passing these gates (>28,000 per sample).

### DNA constructs

Plasmids p2lox and pX330-U6-Chimeric_BB-CBh-hSpCas9 were kindly provided by Michael Kyba (Addgene plasmid #34635; ref. ^71^) and Feng Zhang (Addgene plasmid #42230; ref. ^72^). pEGFP-N3 was from Clontech and the pCR-bluntII-topo clone containing full-length open reading frame of human *SRRT* was from Horizon Discovery (MGC Human SRRT Sequence-Verified cDNA, Accession: BC109117, Clone ID: 40035609 cat# MHS6278-211690300). New constructs were generated as described in Supplementary Data 6 using routine molecular cloning techniques and enzymes from New England Biolabs. *Ammecr1* minigene plasmids were mutagenized as outlined in Supplementary Data 6 using a modified Quikchange site-directed mutagenesis protocol, in which PfuTurbo was substituted with the KAPA HiFi DNA polymerase (Kapa Biosystems, cat# KK2101). All constructs were verified by Sanger sequencing. Maps of all constructs are available on request.

### PCR genotyping

Genomic DNA was prepared and analyzed using PCRBIO Rapid Extract PCR Kit (PCR Biosystems; cat# PB10.24-08) according to the manufacturer’s protocol. Amplified DNA fragments were resolved by electrophoresis in 1–2% agarose gels alongside GeneRuler 1 kb Plus DNA Ladder (Thermo Fisher Scientific, cat# SM1331). Deletion of a cleavage/polyadenylation site-containing fragment in the *Ammecr1* gene was confirmed using Ammecr1_genotype_F/Ammecr1_genotype_R primers (Supplementary Data 7) and Sanger sequencing of the PCR product.

### RNA purification and RT-qPCR analyses

Total RNAs for gene expression analyses were extracted using an EZ-10 DNAaway RNA Miniprep Kit (BioBasic, cat# BS88136). Reverse transcription (RT) was performed at 50 °C for 30 min using SuperScript IV reagents (Thermo Fisher Scientific, cat# 18090200) supplemented with 5 µM of random decamer (N10) primers and 2 units/μl of murine RNase inhibitor (New England Biolabs, M0314L). cDNA samples were analyzed by qPCR using a Light Cycler^®^96 Real-Time PCR System (Roche) and qPCR BIO SyGreen Master Mix (PCR Biosystems; cat# PB20.16). In minigene experiments, total RNAs were isolated from cells using TRIzol (Thermo Fisher Scientific, cat# 15596026), as recommended, with an additional acidic phenol–chloroform (1:1) extraction step. The aqueous phase was precipitated with an equal volume of isopropanol, washed with 70% ethanol, and rehydrated in 80 µl of nuclease-free water (Thermo Fisher Scientific, cat# AM9939). RNA samples were then treated with 4–6 units of Turbo DNase (Thermo Fisher Scientific, cat# AM2238) at 37 °C for 30 min to remove the bulk of DNA contaminants, extracted with equal volume of acidic phenol–chloroform (1:1), precipitated with three volumes of 100% ethanol and 0.1 volume of 3 M sodium acetate (pH 5.2), washed with 70% ethanol and rehydrated in nuclease-free water. Remaining traces of DNA were removed by pre-treating RNA samples with 2 units of RQ1-DNAse (Promega, cat# M6101) per 1 µg of RNA at 37 °C for 30 min. RQ1-DNAse was inactivated by adding the stop solution as recommended and the RNAs were immediately reverse-transcribed using SuperScript IV and random decamer (N10) primers at 50 °C for 30 min. All RT-qPCR primers are listed in Supplementary Data 7. Unless mentioned otherwise, RT-qPCR signals were normalized to expression levels of the Cnot4 housekeeping mRNA. In RAP and RIP RT-qPCR assays, signals in pull-down fractions were normalized to input signals obtained using the same primer pair. In minigene experiments, the RT-qPCR signals detected using primers annealing downstream of the Ammecr1 iCS were normalized to those obtained using upstream primers (see Supplementary Data 7 and Fig. 6b).

### 3′RACE

3′RACE was performed in principle as described^73^. Briefly, total RNAs were extracted from siSrrt-transfected ESCs using an EZ-10 DNAaway RNA miniprep kit. The RT step was done at 50 °C for 60 min using SuperScript IV reagents, 5 µM of the 3′RACE_RT primer (Supplementary Data 7), and 2 units/μl of murine RNase inhibitor. This was followed by two rounds of nested PCR using PCRBIO Ultra Mix Red (PCR Biosystems, PB10.33-05): (1) with the 3′RACE_Q0 primer and a gene-specific primer GS1 and (2) with 3′RACE_Q1 primer and a gene-specific primer GS2 (Supplementary Data 7). The PCR products were then agarose gel-purified using a NucleoSpin gel and PCR clean-up kit (Macherey Nagel cat# 740609.250) and analyzed by Sanger sequencing.

### Northern blotting

Northern blotting was performed using a DIG Northern starter kit (Merck, cat# 12039672910), as recommended. To prepare a U1-specific antisense digoxigenin-labeled probe, pML475 plasmid (Supplementary Data 6) was linearized with PvuII (New England Biolabs), purified using a NucleoSpin gel and PCR clean-up kit, and used as a template for SP6 RNA polymerase. 2.0 × 10^6^ A2lox ESCs were plated in 10 cm gelatinized cell culture dishes in 10 ml of 2i medium and immediately transfected with pmol of either siCtrl or siSrrt premixed with 27 µl of Lipofectamine 2000 and 1.5 ml of Opti-MEM I. Total RNAs were extracted 48 h post-transfection using TRIzol as described above. Purified RNA samples were dissolved in nuclease-free water at ~1 μg/μl and 2-μg aliquots were mixed with 8 μl of the gel loading buffer containing 98% Formamide (Thermo Fisher Scientific, cat# 15515026), 10 mM EDTA, 200 μg/ml bromophenol blue (Thermo Fisher Scientific, cat# 10243420), and 200 μg/ml xylene cyanol (Severn Biotech Ltd, cat# 30-60-01). The samples were then denatured at 70 °C for 3 min, chilled on ice, and resolved by electrophoresis in 8% polyacrylamide gels (acrylamide:bis 29:1; Severn Biotech Ltd, cat# 20-3500-05) containing 8 M urea (Thermo Fischer Scientific, cat# 15505-027) and 1× TBE (Sigma, cat# T4415). RNAs were transferred from the gels to Hybond-N+ membranes (Merck, cat# GERPN1210B) using a Trans-Blot SD semi-dry transfer cell (Bio-Rad) in 0.5× TBE at 3 mA/cm^2^. Membrane were stained with 0.02% methylene blue (Fisher Scientific, cat# 11443697) in 0.3 M sodium acetate pH 5.2 (Sigma, cat# S7899) and photographed. After destaining in 0.2× SSC (Sigma, cat# S6639) and 1% SDS (Promega, cat# H5114) membranes were blocked with DIG Easy Hyb solution at 68 °C for 30 min and hybridized with 100 ng/ml probe in DIG Easy Hyb solution at 68 °C overnight. Membranes were then washed twice in 2× SSC with 0.1% SDS at room temperature and twice in 0.1× SSC with 0.1% SDS at 68 °C, 5 min each wash. The subsequent steps were done at room temperature. Membranes were washed in the Washing buffer containing 0.1 M maleic acid-NaOH, pH 7.5 (Sigma, cat# M0375), 0.15 M NaCl (Sigma, cat# 71376-1KG) and 0.3% (v/v) Tween 20 (Sigma, cat# P9416) for 5 min and blocked in 1× DIG Northern starter kit blocking solution for 30 min. This was followed by incubation with anti-digoxigenin-AP (1:10,000 in blocking solution) for 30 min and two washes with the Washing buffer, 15 min each. Membranes were finally rinsed in the Detection buffer [0.1 M Tris-HCl, pH 9.5 (Thermo Fisher Scientific, cat# BP152-1) and 0.1 M NaCl] for 5 min and chemiluminescence was detected using the CDP-Star reagent and an Odyssey imaging system (LI-COR Biosciences).

### Immunoblotting

Cells grown in six-well plates were washed three times with ice-cold 1× PBS and proteins were extracted using 100–200 µl/well of RIPA lysis buffer (Santa Cruz Biotechnology; cat# sc-364162) supplemented with 1 mM PMSF (New England Biolabs, cat# 8553 S) and the recommended amount of cOmplete EDTA-free protease inhibitor cocktail (Roche, cat# 4693132001). Protein concentrations were determined using a Pierce BCA Protein Assay Kit. Protein samples (10–25 µg) were then incubated at 95 °C for 5 min in 1× Laemmli sample buffer (Bio-Rad; cat# 1610747), chilled on ice, and separated by 4–20% gradient SDS-PAGE (Bio-Rad; cat# 4561096). The proteins were transferred from the gels to nitrocellulose membranes using a Trans-Blot Turbo Transfer System and analyzed using appropriate primary and secondary antibodies (see Supplementary Data 5). Protein bands were detected using an Odyssey imaging system and quantified using the LI-COR Image Studio software (LI-COR Biosciences).

### Co-immunoprecipitation and RNA immunoprecipitation

2.0 × 10^6^ A2lox ESCs were plated in 10 cm gelatinized dishes in 10 ml of 2i medium and immediately transfected with 500 pmol of an appropriate siRNA premixed with 27 µl of Lipofectamine 2000 and 1.5 ml of Opti-MEM I. Forty-eight hours post-transfection cells were washed three times with ice-cold 1× PBS and lysed in 600–700 μl of co-IP/RIP lysis buffer containing 10 mM Tris-HCl, pH 7.5, 150 mM NaCl, 0.5% NP-40/IGEPAL CA-630 (Sigma, I8896) and the recommended amount of cOmplete EDTA-free protease inhibitor cocktail at 4 °C for 30 min. In RIP experiments, co-IP/RIP lysis buffer was additionally supplemented with 100 units/ml of murine RNase inhibitor. The lysates were centrifuged at 16,000 × *g* for 10 min at 4 °C and we used 200–250 μl aliquots of the clarified lysate per individual co-IP/RIP experiment and stored 50 μl aliquots as input controls. The co-IP/RIP aliquots were mixed with 50 μl of Dynabeads protein G beads (Thermo Fisher Scientific, cat# 10003D) preloaded with 5 μg of protein-specific antibodies (Supplementary Data 5) or a non-immune rabbit IgG control (Thermo Fisher Scientific, cat# 10500 C). Lysates were incubated with rotation at 4 °C overnight. In some experiments, lysates were supplemented with 25 units/ml of benzonase (Merck, cat# 70664-3) before mixing them with the beads. Beads were washed three times with 200 μl PBS and 0.5% Tween 20 and bead-associated proteins and RNAs were eluted using 1× Laemmli sample buffer or TRIzol and analyzed by immunoblotting or RT-qPCR, respectively.

### RNA-Seq

For RNA-Seq, A2lox cells were transfected with appropriate siRNAs as described above. Total RNAs were extracted 48 h post-transfection using TRIzol Plus RNA Purification Kit (Thermo Fisher Scientific cat# 12183555). RNAs were eluted in nuclease-free water, quality-controlled using a Bioanalyzer (Agilent) and hybridized with oligo(dT) magnetic beads to isolate the poly(A) RNA fraction used for subsequent library preparation steps. Stranded mRNA sequencing libraries were prepared using the TruSeq Stranded mRNA Library Preparation Kit (Illumina cat## RS-122-2101 and RS-122-2102). Purified libraries were qualified on an Agilent Technologies 2200 TapeStation using a D1000 ScreenTape assay (cat## 5067-5582 and 5067-5583). The molarity of adapter-modified molecules was defined by quantitative PCR using the Kapa Library Quant Kit (Kapa Biosystems; cat# KK4824). Individual libraries were normalized to 10 nM and equal volumes were pooled in preparation for Illumina sequence analysis. Sequencing libraries (25 pM) were chemically denatured and applied to an Illumina HiSeq v4 single-read flow cell using an Illumina cBot. Hybridized molecules were clonally amplified and annealed to sequencing primers with reagents from a HiSeq SR Cluster Kit v4-cBot (Illumina; cat# GD-401-4001). Following transfer of the flow cell to a HiSeq2500 instrument (Illumina; cat## HCSv2.2.38 and RTA v1.18.61), a 50-cycle single-read sequence run was performed using HiSeq SBS Kit v4 sequencing reagents (Illumina; cat# FC-401-4002). All library preparation and sequencing steps were carried out by the Huntsman Cancer Institute High-Throughput Genomics facility, University of Utah, USA.

### 3′RNA-Seq

To characterize global changes in cleavage/polyadenylation patterns, aliquots of total RNA samples prepared as described in the RNA-Seq section were additionally analyzed using 3′-proximal RNA-Seq (3′RNA-Seq). In this case, sequencing-ready libraries were produced using a QuantSeq 3′ mRNA-Seq Library Prep Kit REV (Lexogen, cat# 016.24) following standard procedures, as outlined in the corresponding user guide (Lexogen; https://www.lexogen.com/wp-content/uploads/2018/08/015UG009V0241_QuantSeq_Illumina.pdf) using 200 ng of total RNA as input and using indexed primers for multiplexing. Finished libraries were quality-controlled using a Bioanalyzer (Agilent), using the High Sensitivity DNA assay. Library concentrations were determined using a Qubit dsDNA HS assay (Thermo Fisher scientific, cat# Q32851) and pooled for sequencing based on these quantifications. Sequencing was performed using an Illumina HiSeq2500 (v4) with SR75 High Output at the Vienna Biocenter Core Facilities. A custom sequencing primer (CSP) was used to sequence QuantSeq REV libraries. All library preparation and sequencing steps were carried out by the Lexogen GmbH service team, Austria.

### RAP-Seq

RNA antisense purification (RAP) of formaldehyde-crosslinked samples was performed in principle as described^50^. 3.5 × 10^6^ A2lox ESCs were plated in 10 cm gelatinized dishes in 10 ml of 2i medium and immediately transfected with 500 pmol of siRNAs premixed with 27 µl of Lipofectamine 2000 and 1.5 ml of Opti-MEM I. Medium was replaced once 24 h post-transfection and the culture was incubated for another 24 h.

The cells (~8 × 10^6^) were then washed once with 10 ml PBS and crosslinked with 7 ml of prewarmed 2% formaldehyde freshly diluted in PBS from 16% stock (Thermo Fischer Scientific, cat# 28908) for 10 min at 37 °C with gentle rocking. Formaldehyde was quenched by adding 2.5 M glycine (Sigma, cat# G8898-500G) to a final concentration of 500 mM and incubating the plate at 37 °C for 5 min. Cells were then washed three times with cold PBS and scrapped off the plate in 2 ml of ice-cold Scraping Buffer [1 × PBS and 0.5% DNase/RNase-free BSA (Thermo Fischer Scientific, cat# BP8805)], centrifuged at 1000 × *g* at 4 °C for 5 min, resuspended in hypotonic cell lysis buffer [10 mM HEPES pH 7.5 (Thermo Fischer Scientific, cat# 15630056), 20 mM KCl (Sigma, cat# P9541-1KG), 1.5 mM MgCl_2_ (Sigma, cat# M8266-1KG), 0.5 mM EDTA (Thermo Fischer Scientific, cat# R1021), 1 mM tris(2-carboxyethyl)phosphine (TCEP) (Sigma, cat# 75259-1 G), and 0.5 mM PMSF] and homogenized by douncing ~20 times with microtube pestles (STARLAB, cat# I1415-5390).

The lysates were centrifuged at 3300 × *g* for 7 min at 4 °C and the pellets containing nuclei were resuspended in 1 ml of GuSCN Hybridization Buffer (20 mM Tris-HCl pH 7.5, 7 mM EDTA, 3 mM EGTA (Sigma, cat# E3889-10G), 150 mM LiCl (Sigma, cat# 62476-100G-F), 1% NP-40 (Sigma, cat# I8896-100ML), 0.2% *N*-lauroylsarcosine (Sigma, cat# L7414-10ML), 0.1% sodium deoxycholate (Sigma, cat# D6750-25G), 3 M guanidine thiocyanate (Sigma, cat# G9277-100G), and 2.5 mM TCEP). We solubilized chromatin and fragmented RNA by sonicating the samples for 8 min using a Sonics Vibra-Cell VC130 Ultrasonic Processor equipped with a microtip, with pulser set to 10 s and the amplitude to 20. Lysates were centrifuged at 16,000 × *g* for 10 min at 4 °C and the supernatants were pre-cleared by incubating them for 30 min with MyONE Streptavidin C1 magnetic beads (100 μl original volume, compacted to 25 μl in GuSCN Hybridization Buffer; Thermo Fischer Scientific, cat# 65001) followed by magnetic separation in a DynaMag-2 rack (Thermo Fischer Scientific, cat# 12321D). Small aliquots (~10 μl) of pre-cleared lysates were saved and used later as RNA input controls.

For RAP, pre-cleared lysates from 5 × 10^6^ cells were hybridized with 50 pmol of biotinylated DNA oligonucleotide probe against U1 snRNA (Supplementary Data 7) at 37 °C for 2.5 h with shaking at 1200 r.p.m. in a Thermomixer Compact (Eppendorf). The mixtures were then combined with MyONE Streptavidin C1 magnetic beads (500 μl original volume, compacted to 125 μl in GuSCN Hybridization Buffer) and incubated at 37 °C for 30 min with shaking. The beads were washed at 45 °C with six changes of 500 μl GuSCN Wash Buffer (20 mM Tris-HCl pH 7.5, 10 mM EDTA, 1% NP-40, 0.2% *N*-lauroylsarcosine, 0.1% sodium deoxycyolate, 3 M guanidine thiocyanate, and 2.5 mM TCEP). We then washed the beads once in 500 μl of RNase H Elution Buffer (50 mM Tris-HCl pH 7.5, 75 mM NaCl, 3 mM MgCl_2_, 0.125% *N*-lauroylsarcosine, 0.025% sodium deoxycholate, 2.5 mM TCEP) and once in 100 μl of RNase H Elution Buffer. The beads were subsequently resuspended in 55 μl RNase H Elution Buffer mixed with 7.5 μl RNase H (5 units/μl; New England Biolabs, cat# M0297S) and incubated at 37 °C for 30 min with shaking to digest ssDNA-RNA hybrids and release U1-associated RNAs. The resultant eluates were stored on ice. Second elution step was performed by resuspending the beads in 62.5 μl GuSCN Hybridization Buffer and shaking for 5 min at 37 °C. The first and second eluates were then combined.

To reverse crosslinks, the combined eluates and RNA inputs were mixed with 312.5 μl NLS Elution Buffer (20 mM Tris-HCl pH 7.5, 10 mM EDTA, 2% *N*-lauroylsarcosine, 2.5 mM TCEP), 50 μl 5 M NaCl, and 12.5 μl Proteinase K (Thermo Fischer Scientific, cat# EO0491) and incubated at 60 °C for 2 h. RNAs were then purified by mixing them with 40 μl of Dynabeads MyOne Silane beads (Thermo Fischer Scientific, cat# 37002D) pre-rinsed in RLT buffer (QIAGEN, cat# 79216) and resuspended in 50 μl 5 M NaCl. The suspensions were supplemented with 550 μl of 100% isopropanol, incubated for 2 min at room temperature, and magnetically separated. The beads were washed twice with 600 μl 70% ethanol and dried for 10 min. RNAs were eluted from the beads in 25 μl of nuclease-free water and treated with 2 units of TURBO DNAse in 1× TURBO DNAse buffer for 10 min at 37 °C, without removing the beads from the tubes. The RNAs were then bound to the beads once again by adding 87.5 μl RLT and 112.5 μl isopropanol. The beads were washed twice in 70% ethanol, air-dried and RNAs were eluted from the beads in 25 μl of nuclease-free water.

RNAs were then processed using a NEBNext® rRNA Depletion Kit (New England Biolabs, cat# E6350S) as recommended. RNA-Seq libraries were generated using NEBNext® Ultra8482 II Directional RNA Library Preparation kit (New England Biolabs, cat# E7765S; following the protocol for rRNA Depleted FFPE/Strongly fragmented RNA). Individual libraries were normalized using Qubit, and their size profile was analyzed using TapeStation 4200. Individual libraries were normalized and pooled together accordingly. The pooled library was diluted to ~10 nM for storage. The 10 nM library was denatured and further diluted prior to loading on the sequencer. Paired-end sequencing was performed using a HiSeq4000 75 bp platform (Illumina, HiSeq 3000/4000 PE Cluster Kit and 150 cycle SBS Kit). All library sequencing steps were carried out by the Oxford Genomics Centre, University of Oxford, UK.

### Bioinformatics

All analyses were carried out using mm10 UCSC mouse genome and transcriptome files from Illumina (https://support.illumina.com/sequencing/sequencing_software/igenome.html) and UCSC Genome Browser (http://genome.ucsc.edu/). Canonical UCSC transcripts were used for most of the analyses (knownCanonical UCSC transcripts). Genomic intervals were analyzed using Bedtools or custom R-scripts. Duplicated features with identical genome positions and gene names were removed from the analyses.

For differential gene expression analyses, RNA-Seq reads were aligned with HISAT2 (ref. ^74^) using an mm10 UCSC-based genome index and a list of known splice junctions derived from the UCSC-based mm10 genes.gtf file (ftp://igenome:G3nom3s4u@ussd-ftp.illumina.com/Mus_musculus/UCSC/mm10/Mus_musculus_UCSC_mm10.tar.gz). The alignment was done as follows:

hisat2 -p <n_threads> --rna-strandness F --known-splicesite-infile <hisat2_known_splice_sites.txt> -x <hisat2_genome_index> -U file1.fastq -S file1.sam

HISAT2-mapped reads were converted to BAM format using SAMtools^75^ and assigned to annotated exons from the genes.gtf file using the featureCounts function of the Rsubread R/Bioconductor package^76^ in a strand-specific manner. Differentially expressed genes were then identified using the edgeR package with the estimateGLMRobustDisp function^77,78^. GO-term enrichment was calculated using the goseq package^79^ with gene lengths taken into account. Venn diagrams and gene expression heat maps were generated using VennDiagram (https://cran.r-project.org/web/packages/VennDiagram/) and pheatmap packages (https://cran.r-project.org/web/packages/pheatmap/), respectively. RNA-Seq coverage metaplots were prepared using ngs.plot^80^.

Relative intron coverage (RIC) statistic was calculated as

1 $${\mathrm{RIC}} = I/E,$$

where *I* is the total number of intronic reads and reads spanning junctions between the intron and the adjacent exons by ≥10 nt and *E* is the number of reads matching the adjacent exons and their splice junction. Reads were assigned to the *I* and *E* intervals using Bedtools^81^. Statistical significance of RIC changes was assessed by two-tailed Fisher’s exact test comparison of *I* and *E* values between two experimental conditions. Entries with *I* < 5 and *E* < 10 in both conditions were excluded from the analysis. FDR was calculated by adjusting the resultant *p* values using the Benjamini–Hochberg method.

To analyze changes in cleavage/polyadenylation patterns, 3′-proximal RNA-Seq data were aligned to mm10 genome using Bowtie2 (ref. ^82^) with trimming the first 12 nt to remove poly(A) tail-derived sequences:

bowtie2 --fast --trim5 12 -N 1 -p <n_threads> -x <Bowtie2_genome_index> -U file1.fastq -S file1.sam

Reads with high probability of being primed internally rather than at bona fide poly(A) tails were identified by inspecting corresponding genomic sequences. If 10 consecutive adenosines (with one mismatch allowed) were found within a 20-nt genomic window preceding the read, the read was discarded. The first 5′-terminal nucleotide of the remaining reads mapping to the genome was considered to match a CSs. Individual CSs were then clustered by merging positions spaced by ≤10 nt across all experimental samples. Clusters containing ≥3 reads in at least one sample were kept for further analyses. Clusters were allocated to known intronic and exonic features from the mm10 UCSC annotation using Bedtools.

Incidence of PAS hexamers in a 50 nt window bounded by 40 nt upstream and 10 nt downstream of the middle of CS clusters was calculated using a custom Python script. Cleavage/polyadenylation clusters were considered novel if their middle was >50 nt away from annotated cleavage/polyadenylation sites from the polyA_DB3 database^48^ converted from mm9 to mm10 coordinates using USCS Genome Browser liftOver tool (https://genome.ucsc.edu/cgi-bin/hgLiftOver).

Relative cleavage/polyadenylation site efficiency (RCE) was calculated as

2 $${\mathrm{{RCE}}} = \frac{{N_k}}{{\mathop {\sum }\nolimits_{i = 0}^n N_i}},$$

where *N*_*k*_ is the number of reads matching the cleavage/polyadenylation cluster *k* and *n* is the total number of reads mapping to cleavage/polyadenylation clusters in the same gene. Statistical significance of changes in cleavage/polyadenylation cluster usage was assessed using two-tailed Fisher's exact test by comparing *N*_*k*_ and $(\mathop {\sum }\limits_{i = 0}^n N_i) - N_k$ values between experimental conditions. FDR was calculated using the Benjamini–Hochberg method. We used RCE fold change and FDR values to shortlist significantly regulated CSs. In many cases, we aggregated RCE values for specific genomic ranges (e.g. first introns or 3′UTRs; Figs. 2c and 4b, c and Supplementary Figs. 4d and 10c) and plotted a normalized difference in this statistic between experimental (e) and control (c) samples:

3 $${\mathrm{\Delta }}{\mathrm{RCE}_{\mathrm{norm}}} = \frac{{\mathrm{{RCE}}_{\mathrm{e}}} - {\mathrm{{RCE}}_{\mathrm{c}}}}{{{\mathrm{{RCE}}_{\mathrm{e}}} + {\mathrm{{RCE}}_{\mathrm{c}}}}}.$$

To generate metaplots for 3′RNA-Seq data (Supplementary Fig. 8a, b), genomic regions of interest were split into equally sized bins and a normalized change in 3′-proximal read coverage was calculated for each bin as follows:

4 $$3'{\mathrm{{RC}}_{\mathrm{norm}}} = \frac{{{\mathrm{{RPM}}_{\mathrm{e}} - \mathrm{RPM}_{\mathrm{c}}}}}{{{\mathrm{{RPM}}_{\mathrm{e}} + \mathrm{RPM}_{\mathrm{c}}}}},$$

where RPM_e_ and RPM_c_ are bin-specific coverage data for experimental and control conditions. The bin-specific $3^{\prime}{\mathrm{{RC}}_{\mathrm{norm}}}$ values were then averaged across different genes and plotted after smoothing with Loess function in *R* (span = 0.15). A similar approach was used to prepare Supplementary Fig. 8c where we compared untransformed $3^{\prime}{\mathrm{{RC}}_{\mathrm{norm}}}$ values for 3′UTRs of individual genes. In cases where metaplots for sense and antisense strands had to be shown on the same graph, the antisense strand data were multiplied by −1.

For RAP-Seq data analysis reads were aligned with Bowtie2 using an mm10 UCSC-based bowtie2 genome index as follows:

bowtie2 --fast -N 1 -p <n_threads> -x <Bowtie2_genome_index> -1 file1_1.fastq -2 file1_2.fastq -S file1.sam

Aligned fragments were sorted and converted to genomic intervals using pairedBamToBed12 tool (https://github.com/Population-Transcriptomics/pairedBamToBed12). Fragments with mapping quality <30 were discarded. Piranha peak caller^51^ was used to identify RAP-Seq clusters interacting with U1 snRNA using corresponding input samples as a background:

Piranha -o <output_file> -p 0.01 -a 0.85 -s -l -b 100 -i 100 RAP_1.bed Input_1.bed

Only RAP-Seq clusters present in both replicates were considered for further analysis. Cluster density in specific genomic intervals was calculated using Bedtools. Alternatively, RAP-Seq signal was normalized to input using bamCompare function of the Deeptools package^83^ as follows:

bamCompare -b1 RAP1_merged.bam -b2 Input1_merged.bam --normalizeUsing RPKM --scaleFactorsMethod None --numberOfProcessors <n_threads> --binSize 25 --operation log2 --smoothLength 75 -o log2ratio25_RAP1.bw and visualized using IGV^84^.

To prepare metaplots for RAP-Seq data, genomic regions of interest were divided into 100 bins and the bamCompare-processed values were averaged for each bin using Bedtools and plotted as mean ± SEM.

PhastCons data for placental mammals^52^ were downloaded from UCSC Genome Browser (http://hgdownload.cse.ucsc.edu/goldenpath/mm10/phastCons60way/mm10.60way.phastCons60wayPlacental.bw) and average PhastCons scores were calculated for 50 nt windows bounded by 40 nt upstream and 10 nt downstream of the middle of CS clusters.

RepeatMasker data for RTEs were retrieved from UCSC Genome Browser. RTE consensus sequences were obtained from https://www.girinst.org/repbase/. To generate RTE density metaplots, 2 kb windows centered on the middle of CS clusters were divided into 100 bins and SINE, LINE and LTR coverage for each bin was calculated using Bedtools and plotted as mean ± SEM. Divergence of individual RTEs from consensus sequence was assessed using RepeatMasker milliDiv statistic (base mismatches in parts per thousand; http://www.repeatmasker.org). Clustal Omega (https://www.ebi.ac.uk/Tools/msa/clustalo/) and EMBOSS Matcher (https://www.ebi.ac.uk/Tools/psa/emboss_matcher/nucleotide.html) were used to generate multiple and pairwise DNA sequence alignments, respectively. Strength of putative U1-binding motifs was estimated using MaxEntScan::score5ss^85^.

### Statistical analyses

Unless stated otherwise, all statistical procedures were performed in R, and experimental data were averaged from at least three experiments and shown with error bars representing SD. Data obtained from RT-qPCR and immunoblot quantifications, were typically analyzed using a two-tailed Student’s t-test assuming unequal variances. Correlation analyses were done using Pearson’s product–moment and Spearman and Kendall’s rank correlation methods, as specified in the text. Genome-wide data were typically compared using two-tailed Wilcoxon rank sum test (for non-paired count data), two-tailed Wilcoxon signed rank test (for paired count data), or two-tailed Fisher’s exact test (for categorical data). Where necessary, *p* values were adjusted for multiple testing using Benjamini–Hochberg correction (FDR). Numbers of experimental replicates, *p* values, and the tests used are indicated in the figures and/or figure legends.

### Reporting summary

Further information on research design is available in the Nature Research Reporting Summary linked to this article.

## Supplementary information

Supplementary Information

Peer Review

Reporting Summary

Description of Additional Supplementary Files

Supplementary Data 1

Supplementary Data 2

Supplementary Data 3

Supplementary Data 4

Supplementary Data 5

Supplementary Data 6

Supplementary Data 7

## Data Availability

A reporting summary for this article is available as a Supplementary Information file. The RNA-Seq, 3′RNA-Seq, and RAP-Seq data generated in this study are available from ArrayExpress (E-MTAB-7626, E-MTAB-7635). Publicly available sequencing data used in our study are summarized in Supplementary Data 5. The source data underlying Figs. 1b, c, e–g, 2d, 3b–e, 4d–f, 5e and 6a, c and Supplementary Figs. 1a, b, 2c, 5c, 6a–e, 7e, f, 8d, e, 10d–f, 11a and 12a–d are provided as a Source Data file. All data are available from the corresponding author upon reasonable request.

## Source data

Source Data

## Code availability

Computer code used in this study is described in the Methods and Supplementary Data 5.
