# Supplementary material for: A transcriptome-wide antitermination mechanism sustaining identity of embryonic stem cells
Source: Nat Commun. 2020 Jan 17;11:361. doi: 10.1038/s41467-019-14204-z (PMC6969169; doi:10.1038/s41467-019-14204-z)
Supplement: Supplementary file 1 — Supplementary Information [file 41467_2019_14204_MOESM1_ESM.pdf]

Supplementary Information:

A transcriptome-wide antitermination mechanism sustaining identity of embryonic stem cells

by Kainov and Makeyev

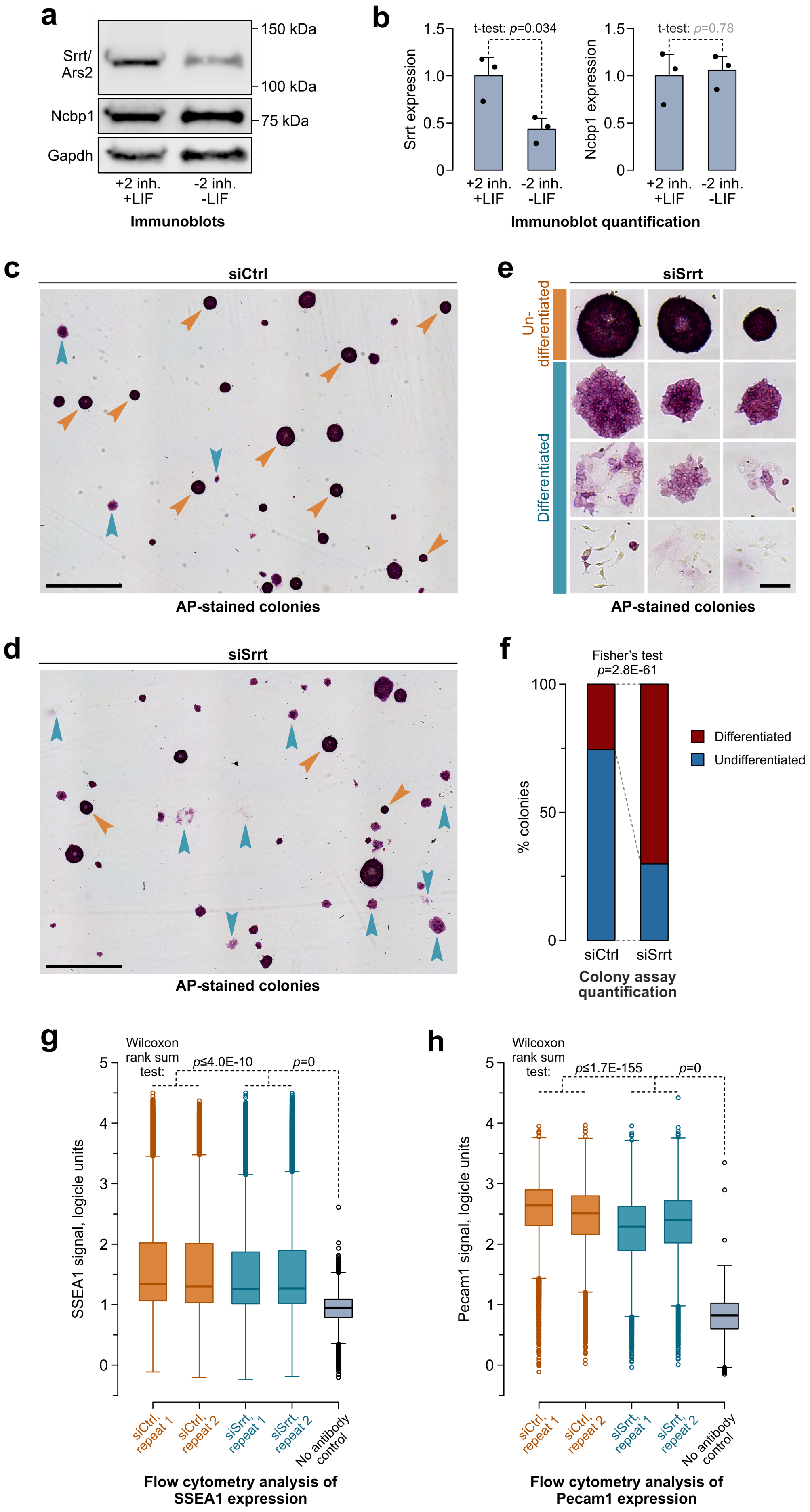

(Supplementary Figure 1. See next page for legend)

Supplementary Figure 1. High expression of Srrt in mouse ESCs is required for maintenance of their undifferentiated status

(**a**) Immunoblot showing that Srrt protein is downregulated in mouse ESCs following a 96-hour incubation in a medium lacking 2i inhibitors and LIF (i.e. compounds required to maintain ESC in an undifferentiated state [1]), as compared to the full medium containing 2i and LIF. Expression levels of an Srrt-interacting partner, nuclear cap-binding protein 1 (Ncpb1/Cbc80), and a housekeeping protein, Gapdh, remain unchanged under these conditions.

(**b**) Quantifications of immunoblot data in (a) for Srrt (left) and Ncbp1 (right). Data were normalized to Gapdh expression levels, averaged from 3 experiments ±SD, and compared by a two-tailed t-test.

(**c-e**) ESCs treated with siSrrt or siCtrl for 24 hours were dissociated and re-plated at 1000 cells per well of a 6-well plate to examine the long-term effect of Srrt knockdown. Colonies formed 7 days post plating were stained for alkaline phosphatase (AP) and imaged. (**c**) Most of the colonies in the siCtrl-treated cultures were dome-shaped and strongly AP-positive, characteristic for undifferentiated ESCs (orange arrowheads). (**d**) In contrast, the siSrrt cultures were dominated by flat differentiated colonies expressing relatively little AP (cyan arrowheads). (**e**) Close-ups of the colonies marked by arrowheads in (d). Scale bars: (c, d) 1 mm; (e) 100 μm.

(**f**) Fisher's exact test confirming that the difference in the undifferentiated/differentiated colony ratio between the siCtrl and siSrrt samples in (c, d) is significant.

(**g, h**) Flow cytometry analyses showing that a 48-hour treatment with siSrrt results in a detectable decrease in the expression of the ESC-enriched surface markers SSEA1 and Pecam1 in comparison with the siCtrl-treated samples. Two biological replicates were analyzed in each case and the distributions were compared by a two-tailed Wilcoxon rank sum test. Box bounds, the first and the third quartiles; thick lines inside the boxes, the medians. Whiskers extend from the first and the third quartile to the lowest and highest data points or, if there are outliers, 1.5× of the interquartile range. Open circles, outliers.

Source data are provided as a Source Data file.

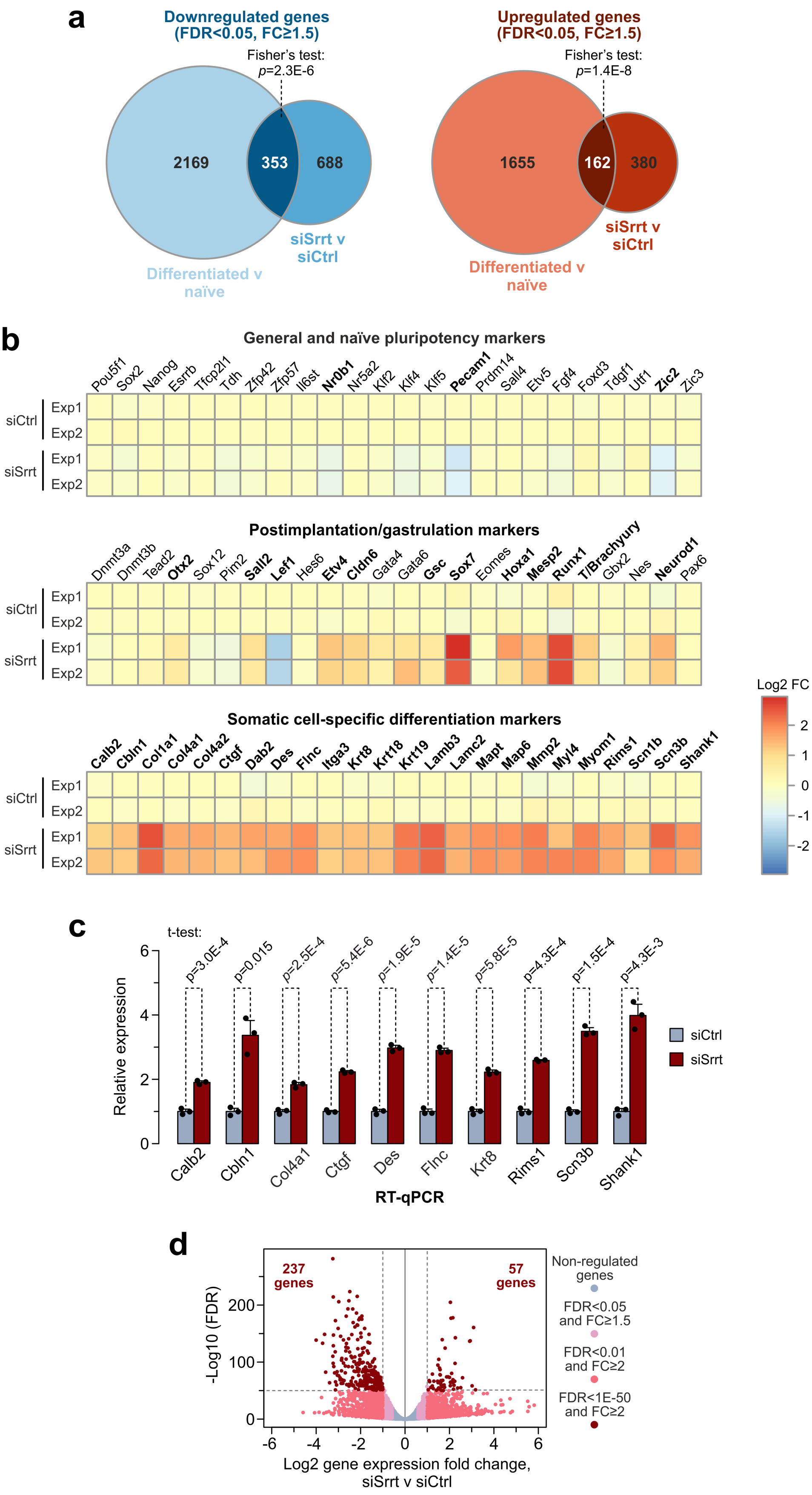

(Supplementary Figure 2. See next page for legend)

Supplementary Figure 2. Srrt knockdown in ESCs induces large-scale changes in the ESC transcriptome

(**a**) Venn diagrams showing significant overlaps between genes regulated by siSrrt and those changing their expression in mouse ESCs undergoing spontaneous differentiation [2] with FC≥1.5 and FDR<0.05. Only genes passing minimum expression cutoffs in both RNA-seq datasets are shown in this comparison.

(**b**) Heat maps showing that Srrt knockdown does not change the expression of many general and naïve pluripotency factors [3-5]. However, it clearly shifts the gene expression pattern towards a more differentiated state by downregulating some ESC-enriched markers (Nr0b1, Pecam1 and Zic2) and upregulating a subset of postimplantation and gastrulation markers and a number transcripts characteristic for terminally differentiated cells. Data shown are from replicated RNA-seq experiments with significantly regulated genes typeset in bold.

(**c**) RT-qPCR validation of siSrrt-upregulated somatic cell-specific markers selected from the corresponding heatmap in (b). These examples include Calb2 (calbindin 2/calretinin expressed in a subset of cortical interneurons and retinal neurons), Cbln1 (cerebellin 1, a protein enriched in postsynaptic structures of Purkinje cells and associated with depression), Col4a1 (a type IV collagen found in epithelial cell basement membranes), Ctgf (a growth factor secreted by vascular endothelial cells), Des (muscle-specific intermediate filament desmin), Flnc (filamin C, a muscle-specific actin cross-linker), Krt8 (a type II keratin expressed in epithelial cells), Rims1 (a regulator of synaptic vesicle exocytosis associated with cone-rod dystrophy 7 and retinitis pigmentosa), Scn3b (sodium voltage-gated channel beta subunit 3 expressed in neurons and muscle cells), and Shank1 (a scaffold protein required for synapse development and function, and associated with diabetic encephalopathy and autism spectrum disorders) (https://www.genecards.org/). The data are averaged from 3 experiments ±SD and compared by a two-tailed t-test. Expression levels in siCtrl-treated samples are set to 1.

(**d**) Volcano plot showing that many genes become either down- or upregulated in response to Srrt knockdown, but the number of downregulated genes noticeably exceeds the number of upregulated ones when using the most stringent cutoffs (FC≥2 and FDR<1E-50).

Source data are provided as a Source Data file.

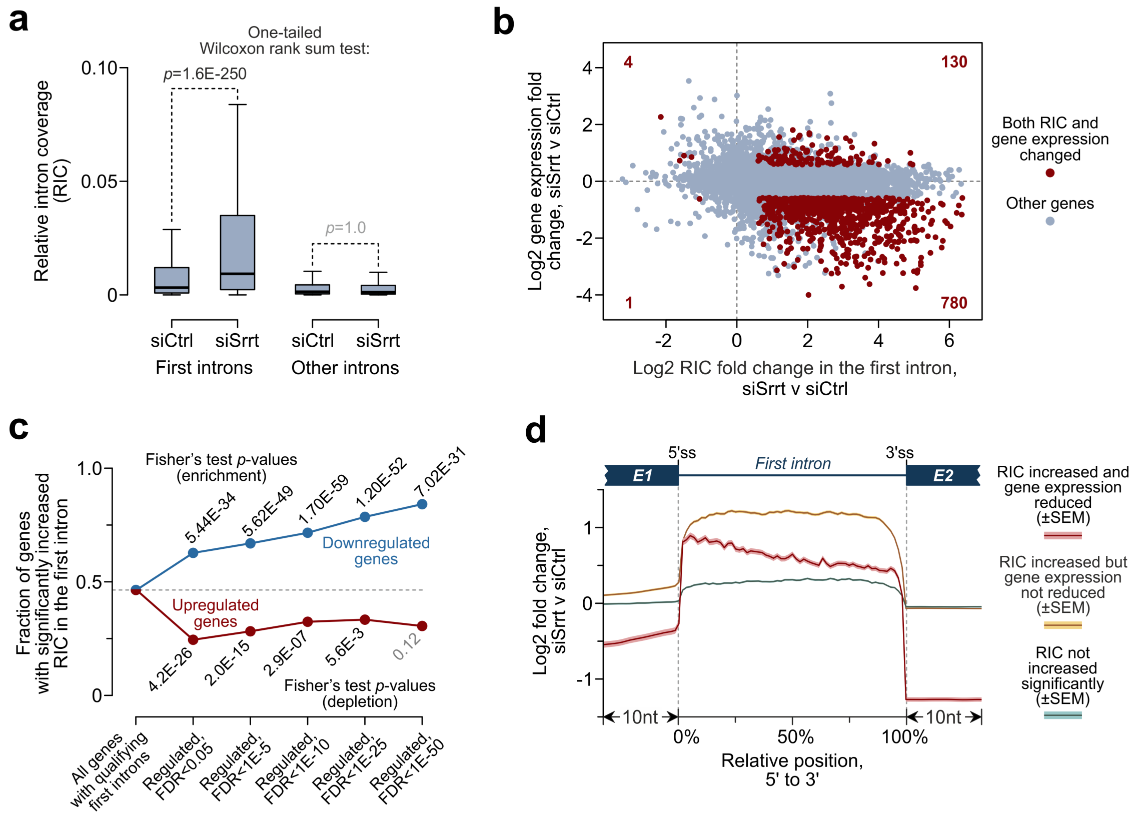

Supplementary Figure 3. Gene expression effects of siSrrt often involve RNA processing changes in first introns

(**a**) Srrt knockdown leads to a significant increase in relative RNA-Seq coverage (RIC statistic; see Methods for details) of first introns. Note that other introns do not show this effect. Box bounds, the first and the third quartiles; thick black lines, the medians. Whiskers extend from the first and the third quartile to the lowest and highest data points or, if there are outliers, 1.5× of the interquartile range.

(**b**) Scatter plot showing that the siSrrt-induced increase in relative RNA-Seq coverage in first introns tends to coincide with downregulation of the corresponding genes. Red dots, genes with significant changes in both RIC (FC≥1.5 and FDR<0.01) and expression levels (FC≥1.5 and FDR<0.05). Gray dots, the rest of the genes. Numbers of significant data points in each of the four quadrants are shown in red.

(**c**) Fisher's exact tests confirming the relationship between increased RIC in the first intron and gene downregulation. The plots show incidence of first introns with significantly increased RIC (FC≥1.5 and FDR<0.01) among all genes qualifying for the analysis and genes regulated ≥1.5-fold with FDR cutoffs ranging from 0.05 to 1E-50. Note that first introns with increased RIC are significantly enriched among downregulated genes and this effect becomes more prevalent as the cutoff stringency is increased. On the other hand, introns with increased RIC are depleted among upregulated genes. The lack of statistical significance for genes upregulated with FDR<1E-50 is due to the fact that relatively few upregulated genes pass this stringent cutoff (see Supplementary Fig. 2d).

(**d**) Metaplots showing that the increase in RNA-Seq coverage is visibly skewed towards the 5' end of first introns for genes characterized by increased RIC (FC≥1.5 and FDR<0.01) and reduced expression (FC≥1.5 and FDR<0.05) (red line), but not for other gene categories (yellow and green lines). Also note a prominent drop-off of the red line in the second exon consistent with possible termination of transcription in the first intron.

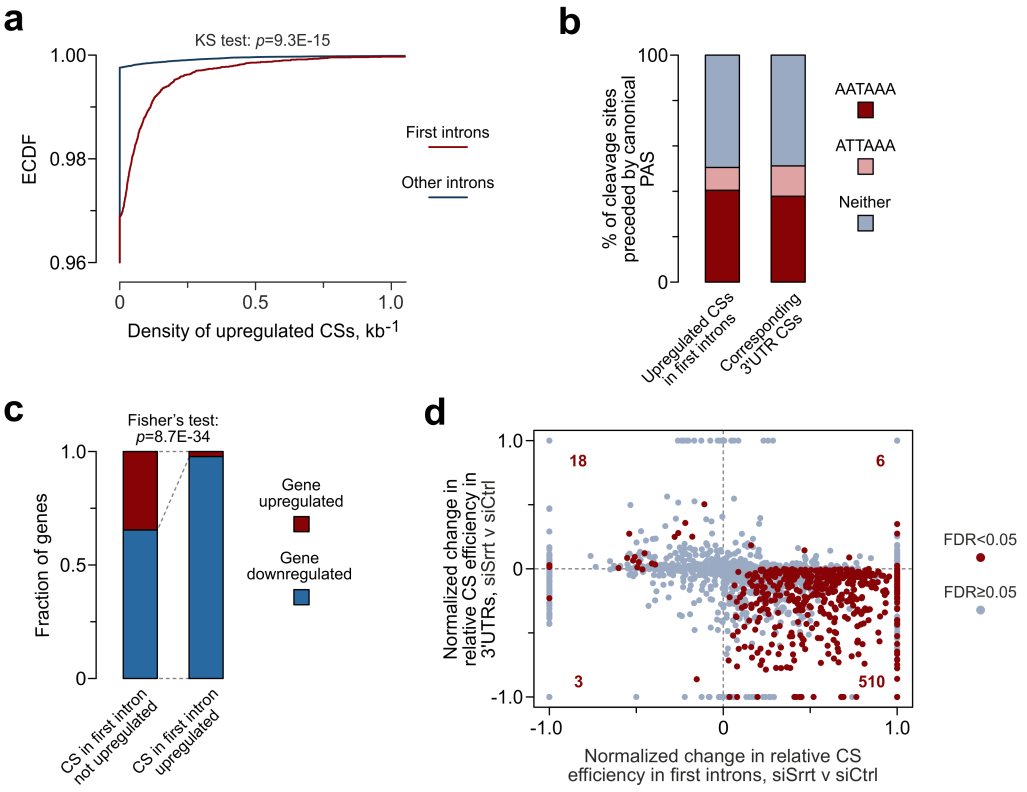

Supplementary Figure 4. Srrt has a transcriptome-wide effect on cleavage/polyadenylation in first introns

(**a**) Cumulative density plots showing that siSrrt-stimulated CSs (fold upregulation ≥2, FDR<0.05) occur more frequently in first introns than in other introns [Kolmogorov-Smirnov (KS) test p=9.3E-15].

(**b**) PAS hexamers AATAAA and ATTAAA precede siSrrt-activated CSs in first introns (fold upregulation ≥2, FDR<0.05) with frequency comparable to that observed for CSs in 3'UTRs of the same genes.

(**c**) Fisher's exact test for Fig. 2c showing that siSrrt-upregulated CSs are significantly over-represented in first introns of downregulated genes.

(**d**) Scatter plot showing that siSrrt-induced activation of CSs in first introns strongly correlates with reduced activity of CSs in the corresponding 3'UTRs (lower right quadrant). Red dots, genes were relative CS efficiency changes in both first introns and 3'UTRs (FDR<0.05); gray dots, other genes. Numbers of significant data points in each quadrant are shown in red.

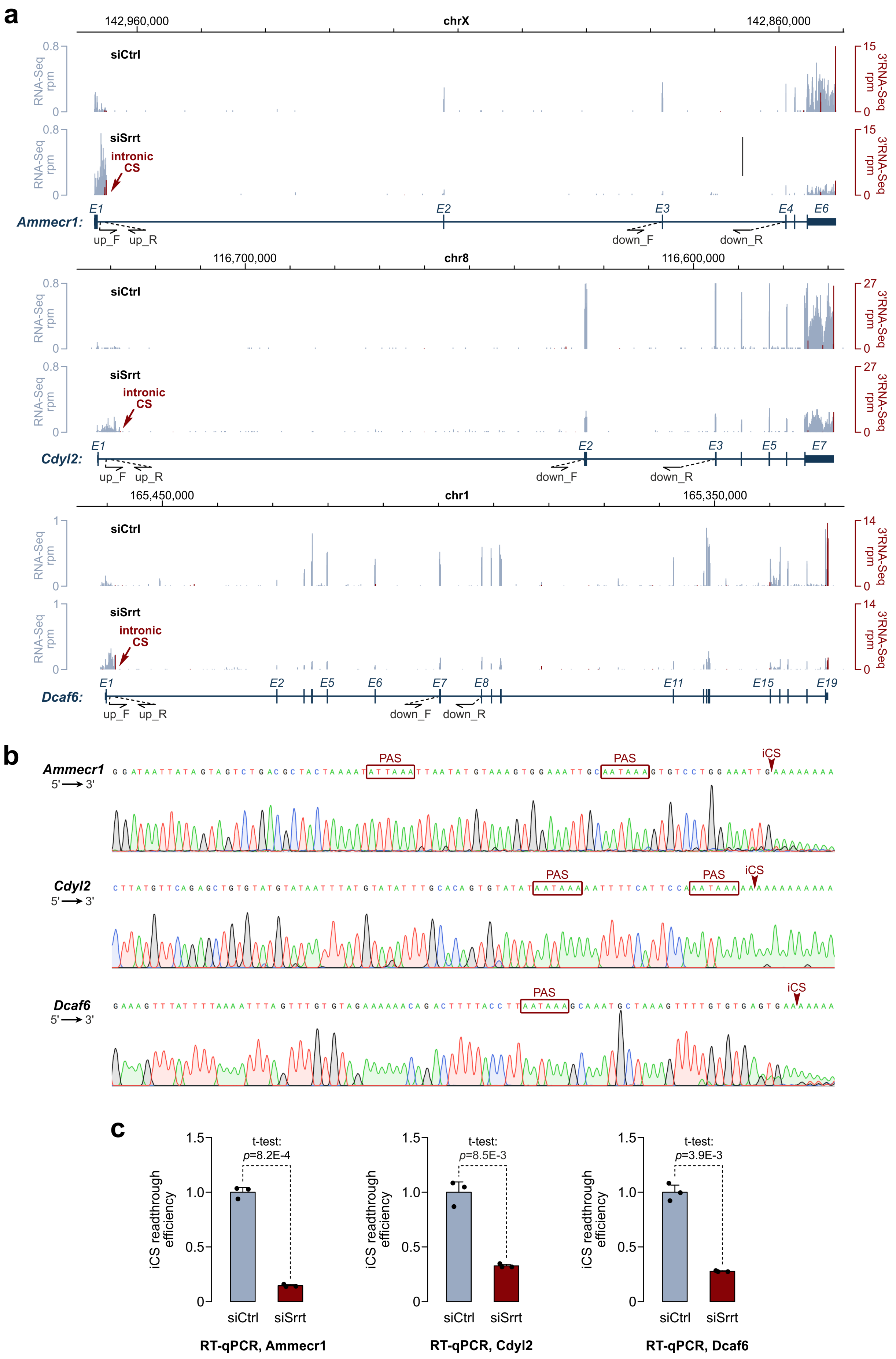

(Supplementary Figure 5. See next page for legend)

Supplementary Figure 5. Examples of genes downregulated by siSrrt through activation of CSs in first introns

(**a**) Complete plots for the 5'- and 3'-proximal close-ups in Fig. 2d showing read-per-million (rpm)-normalized RNA-Seq coverage in gray and rpm-normalized 3'RNA-Seq data in red. Note that activation of CSs in first introns coincides with a reduction in downstream RNA-Seq coverage and 3'RNA-Seq signal intensity in the 3'UTRs. RT-qPCR primers used to analyze intronic CS readthrough efficiency in Fig. 2e and the panel (c) below are shown at the bottom of each gene diagram.

(**b**) Sanger sequencing of 3'RACE PCR products for regulated iCSs in the *Ammecr1*, *Cdyl2* and *Dcaf6* genes. The PAS hexamers are boxed and the predicted iCS positions where the poly(A) tails begin to diverge from the genomic sequences are marked by arrowheads.

(**c**) Intronic CS readthrough efficiencies calculated for the *Ammecr1*, *Cdyl2* and *Dcaf6* genes as ratios between RT-qPCR signals and obtained using the downstream and upstream primer pairs introduced in (a). Data are averaged from 3 experiments ±SD and compared by a two-tailed t-test.

Source data are provided as a Source Data file.

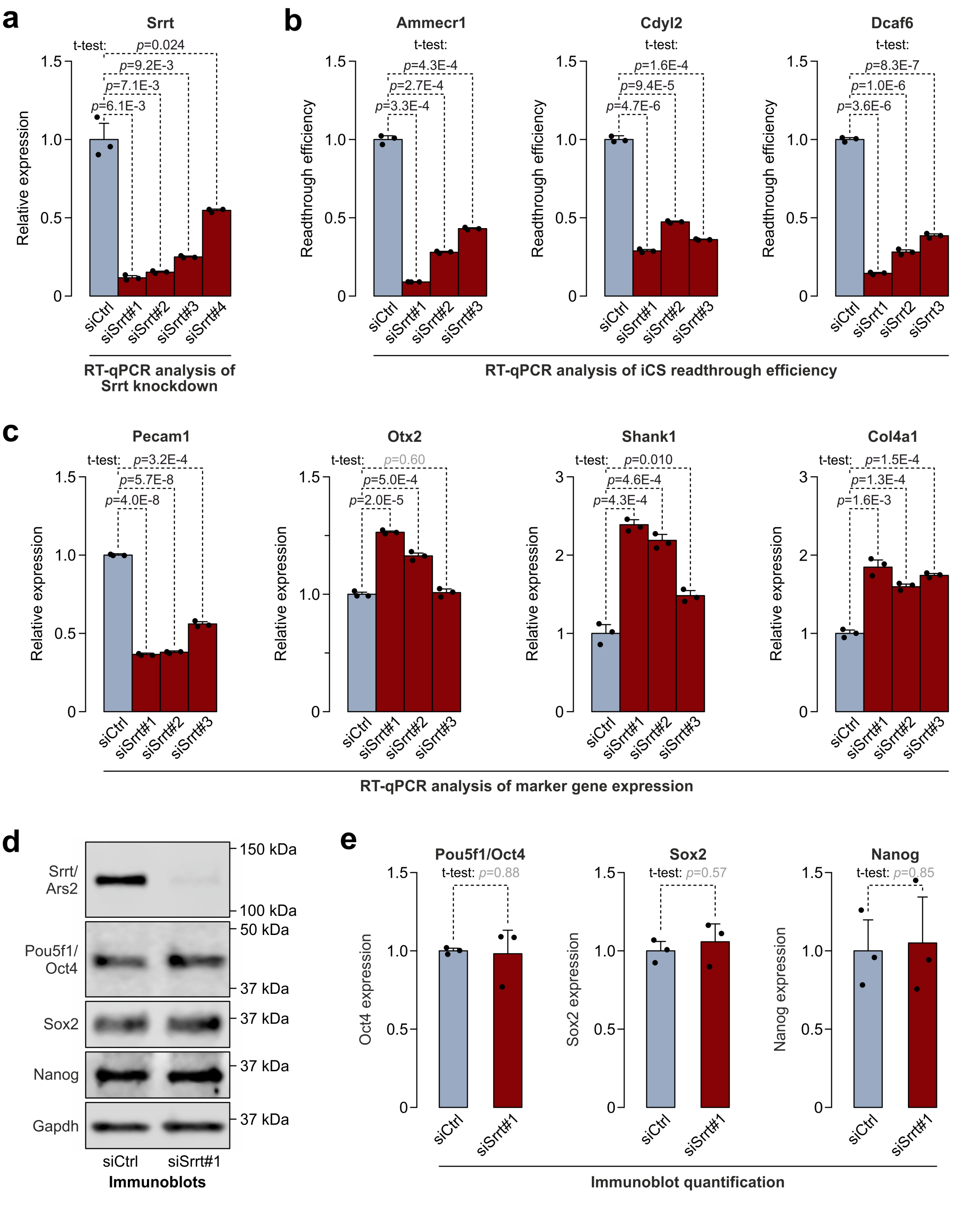

Supplementary Figure 6. Control experiments ruling out siSrrt off-target effects

(**a**) All four individual siRNAs (siSrrt#1, siSrrt#2, siSrrt#3 and siSrrt#4) in the siSrrt mixture used in this study dampen Srrt expression, albeit with different efficiencies.

(**b**) The three most potent siRNAs (siSrrt#1, siSrrt#2 and siSrrt#3) were assayed for their ability to antagonize iCS readthrough in the *Ammecr1*, *Cdyl2* and *Dcaf6* genes as explained in Supplementary Fig. 5c.

(**c**) Gene expression changes brought about by individual Srrt-specific siRNAs are similar to those induced by the siSrrt mixture, with siSrrt#1 and siSrrt#2 generally showing the strongest performance.

(**d**) Immunoblots showing that even the best Srrt-specific siRNA, siSrrt#1, does not alter the expression levels of the pluripotency factors Pou5f1/Oct4, Sox2 and Nanog.

(**e**) Quantitation of the band intensities in (d) normalized to Gapdh and siCtrl.
The RT-qPCR (a-c) and the immunoblot quantification data (e) were averaged from 3 experiments ±SD and compared by a two-tailed t-test.

Source data are provided as a Source Data file.

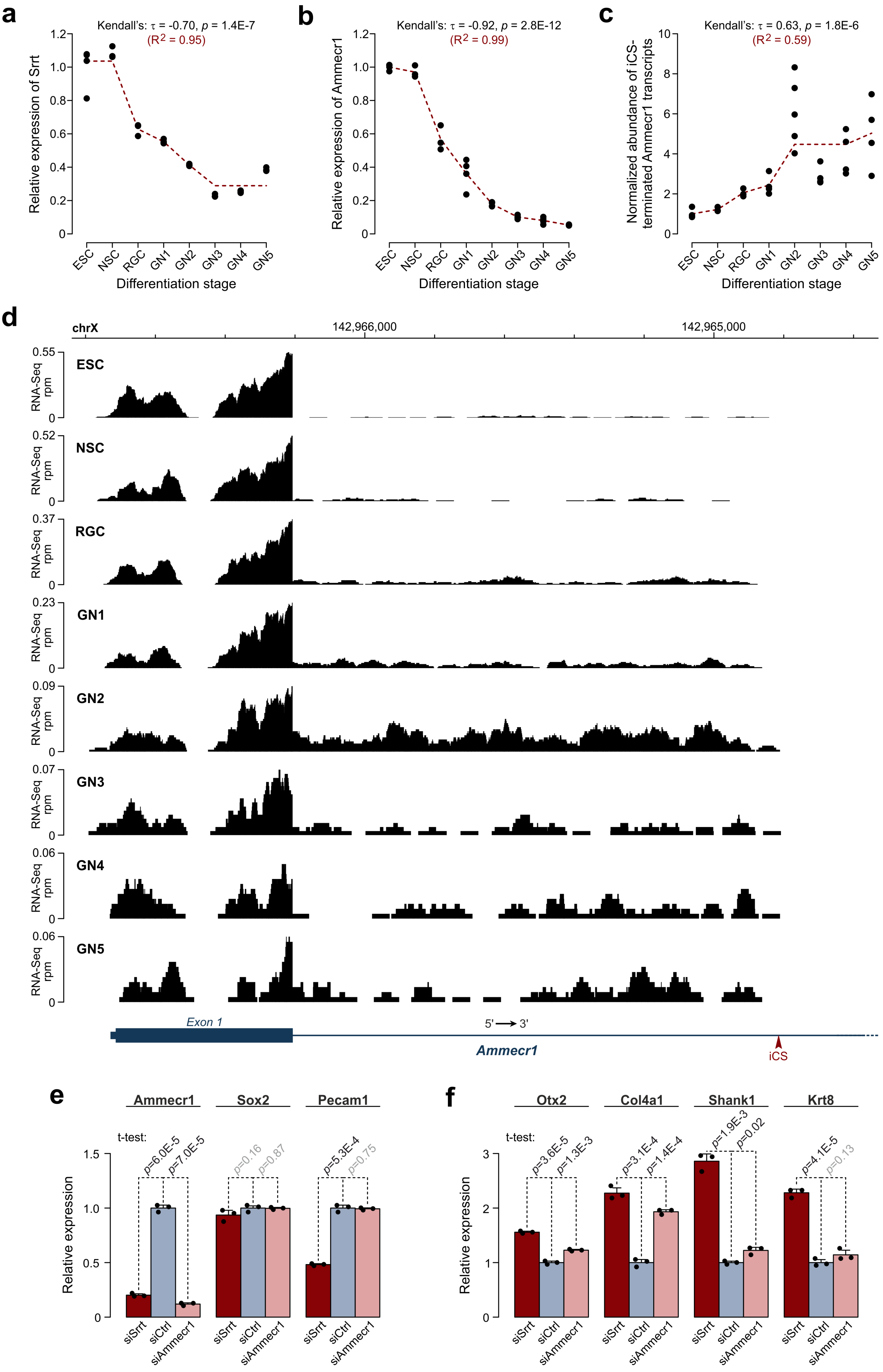

(Supplementary Figure 7. See next page for legend)

Supplementary Figure 7. *Ammecr1* is an important Srrt target showing expected expression dynamics during neuronal differentiation of mouse ESCs

(**a-c**) Longitudinal changes in *Srrt* and *Ammecr1* expression in previously published RNA-seq analysis mouse ESC differentiation time course [6] resolving the ESC, NSC and radial glial cell (RGC) stages, as well as 5 progressive stages of glutamatergic neuronal maturation (GN1-GN5). The overall expression of (a) *Srrt* and (b) *Ammecr1* decrease during the course of differentiation. (c) Conversely, exon 1-normalized RNA-seq coverage for the region of the *Ammecr1* first intron between the 5'ss and the Srrt-regulated iCS shows an increasing trend. Black circles, experimental data. Red dashed lines, trend curves fitted using (a, b) the decr or (c) the incr routines of the cgam R package [7]. The routines were selected based on the sign of the Kendall's rank correlation statistics (τ) indicated at the top of each graph along with the corresponding p-values. The R^2 goodness-of-fit statistics are shown in red.

(**d**) RNA-seq coverage plots for the 5'-proximal region of *Ammecr1* showing that the relative abundance of iCS-terminated transcripts tends to increase during neuronal differentiation [6].

(**e, f**) RT-qPCR data averaged from 3 experiments ±SD and compared by a two-tailed t-test suggest that siRNA knockdown of Ammecr1 (siAmmecr1) partially recapitulates gene expression effects induced by siSrrt.

Source data are provided as a Source Data file.

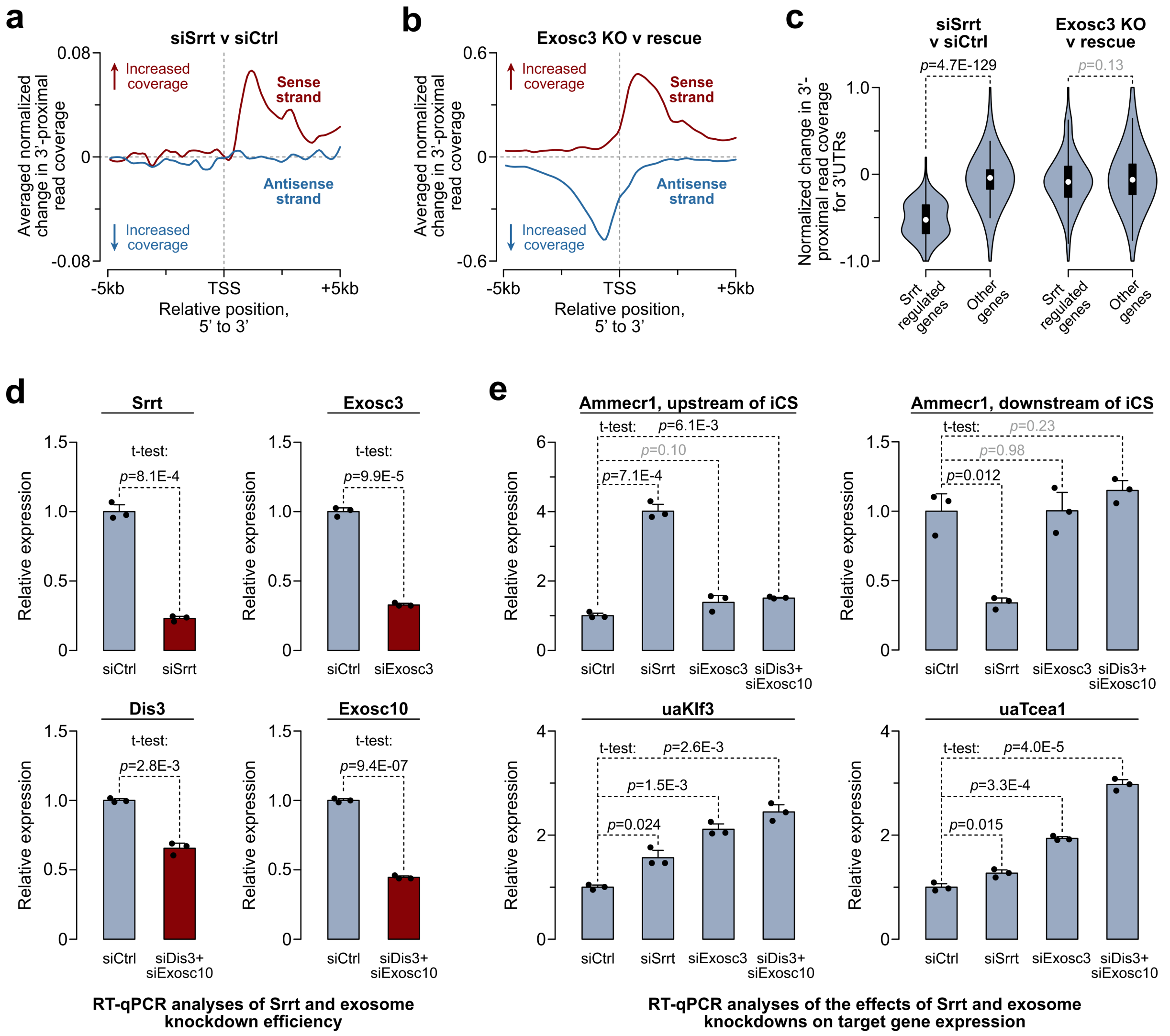

Supplementary Figure 8. The exosome complex does not play a major part in Srrt-mediated readthrough of iCSs in mouse ESCs

(**a, b**) Metaplots illustrating distinct responses of Srrt-dependent genes (first intron CS upregulation ≥2-fold, FDR<0.05 and gene upregulation ≥1.5-fold, FDR<0.05) to siSrrt and exosome knockout. (a) Srrt knockdown leads to noticeable accumulation of prematurely terminated sense transcripts without changing the expression of transcription start site (TSS) proximal antisense transcripts. (b) Analysis of the same group of genes for published 3'-proximal RNA-Seq data (2P-Seq[8]) shows that mouse ESCs lacking a core exosome subunit (Exosc3 KO) accumulate both sense and antisense TSS-proximal transcripts in comparison with an Exosc3-rescue control.

(**c**) The usage of CSs in 3'UTRs of Srrt-dependent genes [same cutoffs as s in (a, b)] is significantly reduced in mouse ESCs in response to Srrt knockdown but not Exosc3 KO. Normalized change in 3'-proximal read coverage was calculated as explained in Methods and the samples were compared using a two-tailed Wilcoxon rank sum test. Violin plot outlines show kernel density estimates of probability densities; open circles, the medians; bounds of the black boxes, the first and the third quartiles. Whiskers extend from the first and the third

quartile to the lowest and highest data points or, if there are outliers, 1.5× of the interquartile range.

(**d**) Mouse ESCs were transfected with siCtrl, siSrrt, an siRNA against Exosc3 (siExosc3) or a mixture of siRNAs against catalytic subunits of the exosome, Dis3 and Exosc10 (siDis3+siExosc10). Knockdown efficiencies of the corresponding targets were analyzed 48 hours later by RT-qPCR.

(**e**) RT-qPCR analyses of gene expression effects of siSrrt, siExosc3 and siDis3+siExosc10. Note robust accumulation of 5'-proximal Ammecr1 transcripts and downregulation of the full-length Ammecr1 mRNA in response to siSrrt but not exosome-specific siRNAs. Conversely, exosome-specific knockdowns lead to stronger upregulation of TSS-proximal upstream antisense transcripts (uaKlf3 and uaTcea1) compared to siSrrt. Data in (d, e) were averaged from 3 experiments ±SD and compared by a two-tailed t-test.

Source data are provided as a Source Data file.

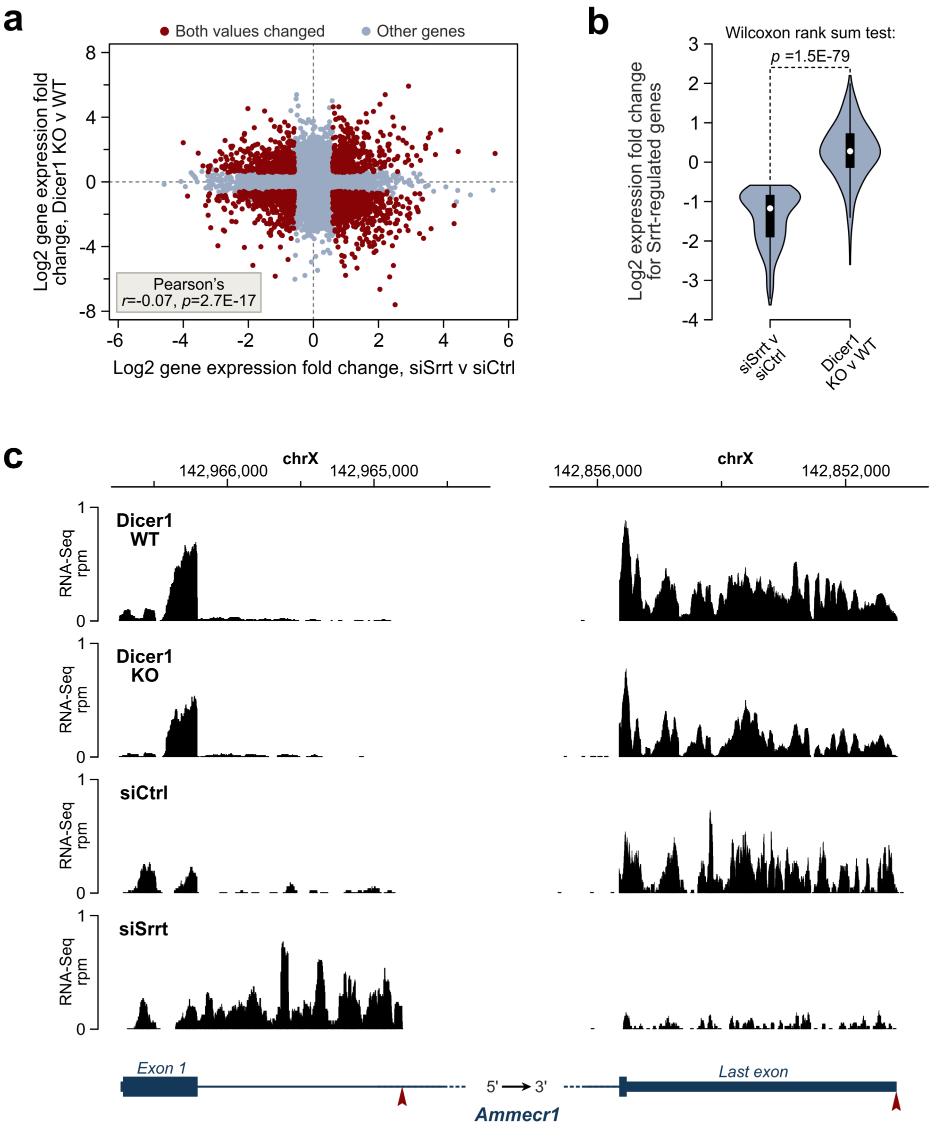

Supplementary Figure 9. Dicer-dependent small RNAs do not play a major part in Srrt-mediated readthrough of iCSs in mouse ESCs

(**a**) Gene expression changes induced by Srrt knockdown (siSrrt v siCtrl) do not correlate with those triggered by knockout of a key microRNA biogenesis factor, Dicer, in mouse ESCs[9] (Dicer1 KO v WT).

(**b**) Dicer1 KO does not generally alter the expression of the Srrt-dependent genes (first intron CS upregulation ≥2-fold, FDR<0.05 and gene upregulation ≥1.5-fold, FDR<0.05). Violin plot outlines, kernel density estimates of probability densities; open circles, the medians; bounds of the black boxes, the first and the third quartiles. Whiskers extend from the first and the third quartile to the lowest and highest data points or, if there are outliers, 1.5× of the interquartile range.

(**c**) RNA-seq coverage plots for *Ammecr1* showing that Dicer1 KO fails to recapitulate the premature termination effect induced by siSrrt.

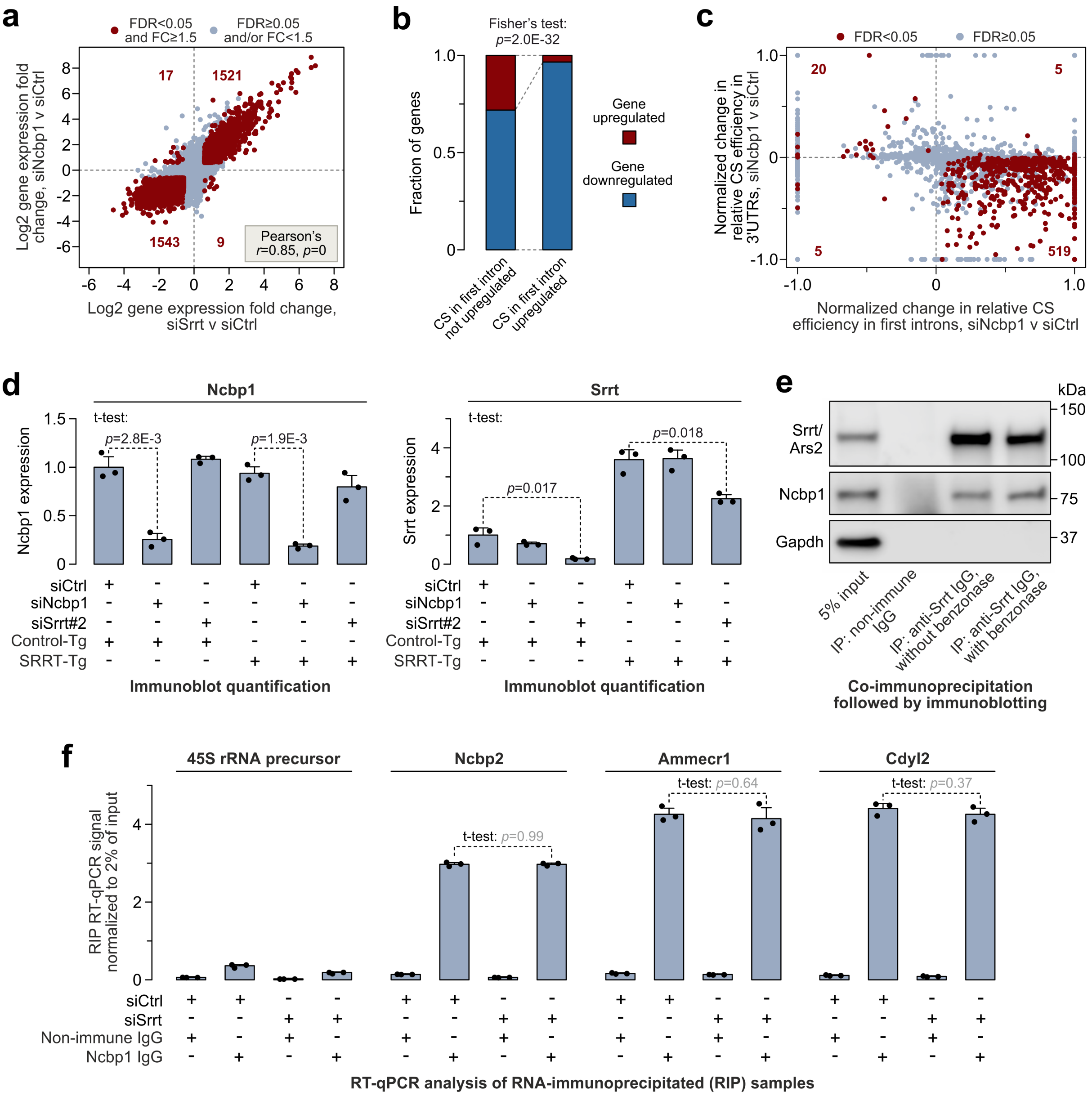

Supplementary Figure 10. Repression of iCSs by Srrt depends on its interaction with CBC

(**a**) Changes in gene expression induced by Ncbp1 knockdown in mouse ESCs correlate (Pearson's *r*=0.85, *p*=0) with gene expression effects of siSrrt. Red dots, genes regulated in response to both siSrrt and siNcbp1 (FC≥1.5 and FDR<0.05). Gray dots, the rest of the genes.

(**b**) Fisher's exact test for Fig. 4c showing that siNcbp1-upregulated CSs are significantly over-represented in first introns of downregulated genes.

(**c**) Scatter plot showing that siNcbp1-induced upregulation of CSs in first introns strongly correlates with downregulation of CSs in the corresponding 3'UTRs (lower right quadrant). Red dots, genes were relative CS efficiency changes in both first introns and 3'UTRs (FDR<0.05); gray dots, other genes. Numbers of significant data points in each quadrant in (a, c) are shown in red.

(**d**) Quantification of the Srrt and Ncbp1 immunoblots in Fig. 4d averaged from 3 experiments ±SD and compared by a two-tailed t-test.

(**e**) Co-immunoprecipitation experiment indicating that Srrt interacts with Ncbp1 in mouse ESCs in a nucleic acid-independent manner. Proteins were pulled down with or without benzonase using either Srrt-specific or non-immune antibodies and analyzed by immunoblotting with Srrt, Ncbp1 or Gapdh-specific antibodies.

(**f**) RNA immunoprecipitation (RIP) with Ncbp1-specific antibodies showing that Srrt knockdown does not detectably change interaction of Ncbp1 with Srrt-regulated (Ammecr1 and Cdyl2) or control (Ncbp2) mRNAs. Data are averaged from 3 experiments ±SD and compared by a two-tailed t-test.

Source data are provided as a Source Data file.

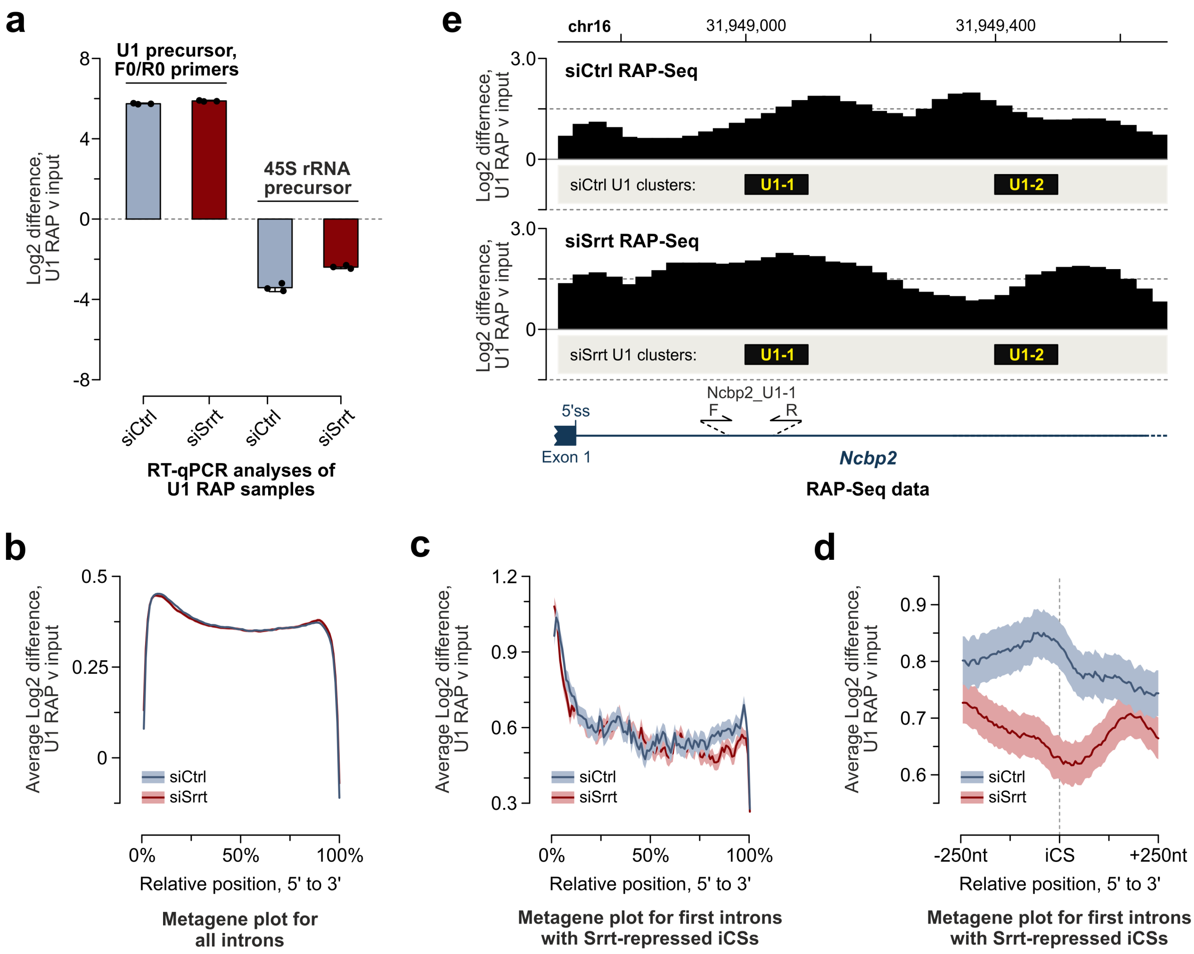

Supplementary Figure 11. RAP-seq analysis of the role of Srrt in U1 recruitment

(**a**) RT-qPCR confirming that the U1 RAP procedure enriches U1 snRNA precursor and depletes ribosomal RNA precursor, as expected. Data are averaged from 3 experiments ±SD.

(**b-d**) U1 RAP-Seq metaplots showing expected bias of U1-binding sequences towards the 5' end of (b) all and (c) first introns containing Srrt-regulated iCSs in both the siCtrl and the siSrrt datasets. (d) Inspection of the iCS-adjacent region suggests that the U1 occupancy immediately upstream of the iCSs is somewhat higher than downstream of these sites in the siCtrl samples and that the siSrrt treatment reduces U1 binding in the iCS-proximal region.

(**e**) Input-normalized RAP-Seq coverage profile and Piranha clusters (U1-1 and U1-2) for the first intron of *Ncbp2*, a control gene not regulated by siSrrt. Primers used in the RT-qPCR validation experiment in (Fig. 5e) are shown at the bottom.

Source data are provided as a Source Data file.

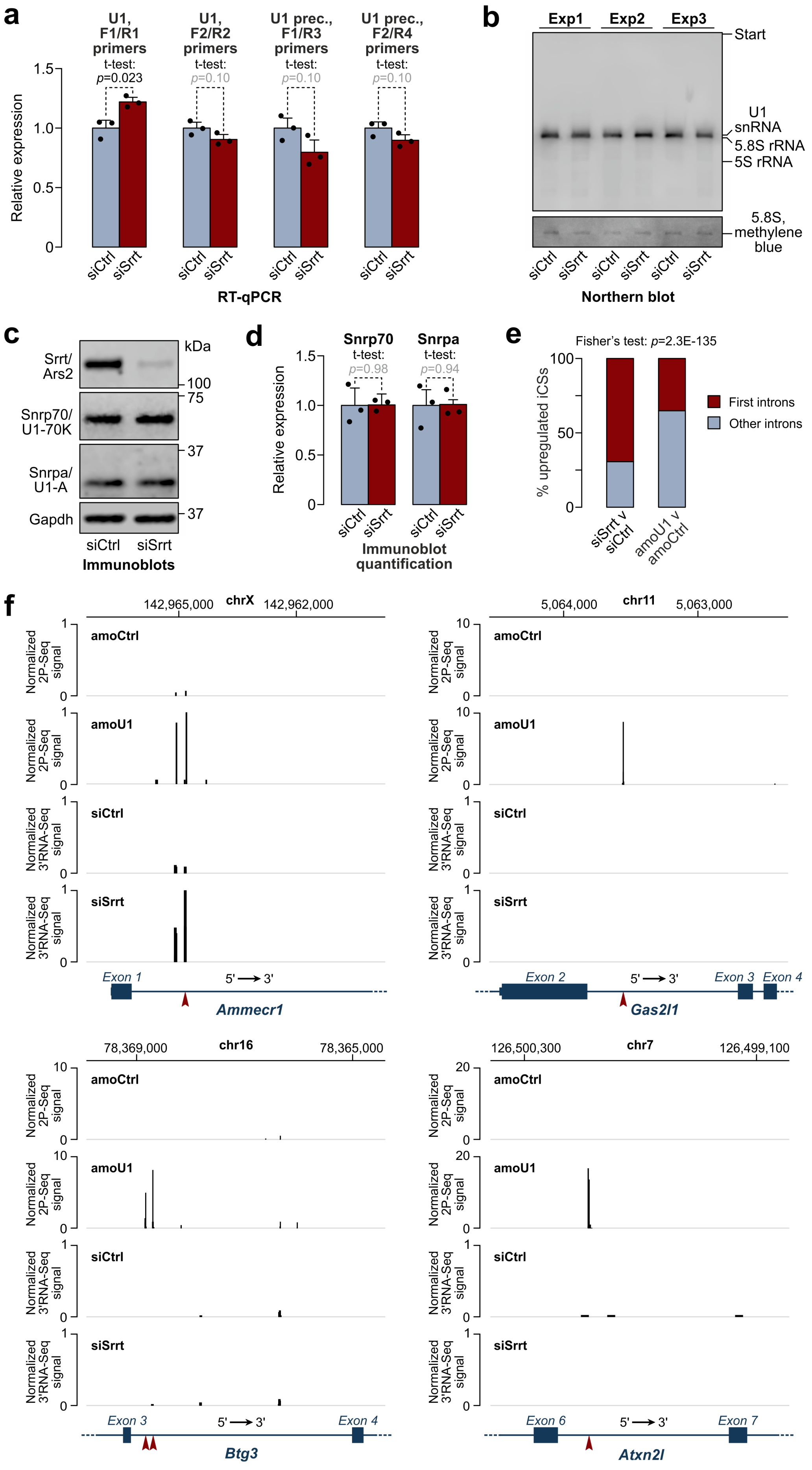

(Supplementary Figure 12. See next page for legend)

Supplementary Figure 12. Srrt knockdown in ESCs has no detectable effect on U1 snRNP biogenesis

(**a**) RT-qPCR assays indicating that siSrrt has no major effect on the abundance of mature U1 snRNA or its 3'-extended precursors. We used primer pairs targeting two different genomic variants of U1.

(**b**) Northern blot confirming that Srrt knockdown has no effect on U1 snRNA expression. Three biological replicates (Exp1, Exp2 and Exp3) were analysed side by side. The U1 snRNA Northern signal and the positions of the 5.8S and 5S rRNAs deduced from the methylene blue-stained membrane are marked on the right. Methylene blue-stained 5.8S rRNA bands are also shown at the bottom to provide a lane loading control.

(**c**) Immunoblot analysis and (**d**) its quantification showing that siSrrt does not alter the abundance of the U1 snRNP-specific proteins Snrpa/U1-A and Snrnp70/U1-70K. Data in (a, d) are averaged from 3 experiments ±SD and compared by a two-tailed t-test.

(**e**) Fisher's exact test showing that iCSs activated by treating ESCs with a U1-specific antisense morpholino oligonucleotide (amoU1) v scrambled control (amoCtrl)[8] lack the strong bias towards the first introns observed for Srrt knockdown (siSrrt v siCtrl).

(**f**) 2P-Seq/3'RNA-seq data showing that amoU1 upregulates the iCS in the first intron of *Ammecr1* but differs from siSrrt by additionally activating iCSs in non-first introns. To facilitate comparison, the morpholino and the siRNA data were normalized to the height of the Ammecr1 iCS peak in the amoU1 and the siSrrt samples, respectively. Positions of regulated iCSs are marked by arrowheads.

Source data are provided as a Source Data file.

Ammecr1

```
Mouse:     5'..AAATATTAAATTAATATGTAAAGTGGAAATTGCAATAAAGTGTCCTGGAAATTGTGCGTT..3'
Rat:           AAACATTAAATTAATGTGTAAAGTGGAAATTACAATAAAGTGTCCTGGAAATTGTGTGTT
Human:         AAATATTAAATTAATATGTAAAGTGGAAATTACAATAAAGTGTCCTGGAAATTGTGCATT
Bushbaby:      AAATATTCAATTAATATGTAAAGTGGAAATTATAATAAAGTGTCCTGGAAATTGTGTATT
Cow:           AAATATTCATTTAATATGTAAAGTGGAAATTGCAATAAAGTATCCTGGAAATTGTAAATT
Dolphin:       AAATATTCAATTAATATGTAAAGTGGAAATTACAATAAAGTGTCCTGGAAATTATAAATT
Armadillo:     AAATATTAGACTAATATGTAAAGTAGAAGCTGTAATAAAGTGTCCTGGAAATTGTAAACT
               *** ***    **** ******** ***  *  ******** *********** *    *
```

Cdyl2

```
Mouse:  5'..TATATAATAAAA-----ATTTTCATTCCAAATAAAAACA----------ACAGAATACTTTTTCTTTGTTGAAAGA..3'
Rat:        TATATAATAAAC-----ATTTTCATTCCAAATAAAAAGC---------AACAGAACATTTTCCCTTTGTTGAAAGA
Human:      ------------------------------------------------------------------------------
Bushbaby:   TATGAAAATACTGCTTTCA-TTCATTCCAAATAAAATAAGTAAT---AATAGGATAATTTTAGTTTTATTCAAAGA
Cow:        TATATAAATACTGCTTTTGTTTCATTCCAAATAAAATAAACGA------TAATAGGATTTTCGTTTTGTTGAAAGA
Dolphin:    TAAAAAAATACTGCTTTCATTTCATTCCAAATAAAATAAATAACAATAATAATAGGATTTTAGTTTTGTTGAAAGA
Armadillo:  TAAGGAAATGTTACTTTTATTTCATTACAAATAAAACATAGA-----------ATAATTTTAGTTTTGGTGAAAGA
            **   **           ****** *********                 *  * ***   ***  * *****
```

Dcaf6

```
Mouse:      5'..TTTGTGTAGAAAAAACAGACTTT-TACCTTAATAAAGCAAATGCTAAAGTT..3'
Rat:            TTTGTCTAGAAAGAACAGGTTTT-TACCTTAATAAAGCAAATGCTAAAGTT
Human:          TTTGCGGAGAAAGAACTTAATTT-TAGCTTAATAAAACAAACGTTAACATT
Bushbaby:       TTTGCTGGGAAAGAACTTAAGTTTTAGCTTAATAAAAAAACAAATGTTAGC
Cow:            CTTGCTGAGAAATAATTTTA------GTTTAATAAAAAGAATACATGTTCT
Dolphin:        CTTGCTGAGAAAGAACTTAACTT-TAGCTTAATAAAAAAAACTTGTGTTCT
Armadillo:      ---------------------------------------------------
                 ***    **** **            ********   *
```

Supplementary Figure 13. Examples of Srrt-regulated iCSs conserved in evolution

Multiple sequence alignments showing considerable interspecies conservation of the Srrt-regulated iCS region in *Ammecr1*, *Cdyl2* and *Dcaf6* genes. Sequences were downloaded from the Multiz tract of UCSC Genome Browser (https://genome.ucsc.edu) and the iCS-proximal regions were realigned using Clustal Omega (https://www.ebi.ac.uk/Tools/msa/clustalo). Invariant positions are marked by asterisks.

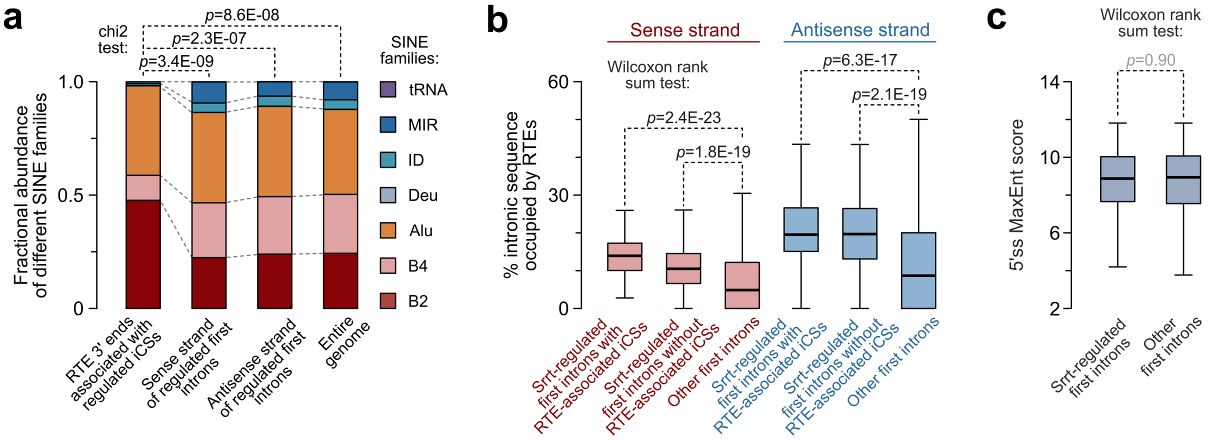

Supplementary Figure 14. Relationship between Srrt regulation and retrotransposition

(**a**) Members of the B2 family are enriched amongst iCS-associated SINE repeats.

(**b**) The overall RTE density is significantly higher in Srrt-regulated first introns than in non-regulated first or non-first introns.

(**c**) Predicted strengths of splice donor sites (5'ss) in Srrt-regulated and non-regulated first introns are statistically indistinguishable. In (b, c), box bounds, the first and the third quartiles; thick black lines, the medians. Whiskers extend from the first and the third quartile to the lowest and highest data points or, if there are outliers, 1.5× of the interquartile range. Outliers are not shown.

Supplementary References

1 Martello, G. & Smith, A. The nature of embryonic stem cells. *Annu Rev Cell Dev Biol* **30**, 647-675, doi:10.1146/annurev-cellbio-100913-013116 (2014).

2 Guo, G. *et al.* Serum-Based Culture Conditions Provoke Gene Expression Variability in Mouse Embryonic Stem Cells as Revealed by Single-Cell Analysis. *Cell Rep* **14**, 956-965, doi:10.1016/j.celrep.2015.12.089 (2016).

3 Dunn, S. J., Li, M. A., Carbognin, E., Smith, A. & Martello, G. A common molecular logic determines embryonic stem cell self-renewal and reprogramming. *EMBO J* **38**, doi:10.15252/embj.2018100003 (2019).

4 Kalkan, T. *et al.* Complementary Activity of ETV5, RBPJ, and TCF3 Drives Formative Transition from Naive Pluripotency. *Cell Stem Cell* **24**, 785-801 e787, doi:10.1016/j.stem.2019.03.017 (2019).

5 Kalkan, T. *et al.* Tracking the embryonic stem cell transition from ground state pluripotency. *Development* **144**, 1221-1234, doi:10.1242/dev.142711 (2017).

6 Hubbard, K. S., Gut, I. M., Lyman, M. E. & McNutt, P. M. Longitudinal RNA sequencing of the deep transcriptome during neurogenesis of cortical glutamatergic neurons from murine ESCs. *F1000Res* **2**, 35, doi:10.12688/f1000research.2-35.v1 (2013).

7 Liao, X. & Meyer, M. C. cgam: An R Package for the Constrained Generalized Additive Model. *Journal of Statistical Software* **89**, 1-24, doi:10.18637/jss.v089.i05 (2019).

8 Chiu, A. C. *et al.* Transcriptional Pause Sites Delineate Stable Nucleosome-Associated Premature Polyadenylation Suppressed by U1 snRNP. *Mol Cell* **69**, 648-663 e647, doi:10.1016/j.molcel.2018.01.006 (2018).

9 Zheng, G. X., Do, B. T., Webster, D. E., Khavari, P. A. & Chang, H. Y. Dicer-microRNA-Myc circuit promotes transcription of hundreds of long noncoding RNAs. *Nat Struct Mol Biol* **21**, 585-590, doi:10.1038/nsmb.2842 (2014).

10 Iacovino, M. *et al.* Inducible cassette exchange: a rapid and efficient system enabling conditional gene expression in embryonic stem and primary cells. *Stem Cells* **29**, 1580-1588 (2011).

11 Cong, L. *et al.* Multiplex genome engineering using CRISPR/Cas systems. *Science* **339**, 819-823, doi:10.1126/science.1231143 (2013).

12 Langmead, B. & Salzberg, S. L. Fast gapped-read alignment with Bowtie 2. *Nat Methods* **9**, 357-359, doi:10.1038/nmeth.1923 (2012).

13 Kim, D., Langmead, B. & Salzberg, S. L. HISAT: a fast spliced aligner with low memory requirements. *Nat Methods* **12**, 357-360, doi:10.1038/nmeth.3317 (2015).

14 Quinlan, A. R. & Hall, I. M. BEDTools: a flexible suite of utilities for comparing genomic features. *Bioinformatics* **26**, 841-842, doi:10.1093/bioinformatics/btq033 (2010).

15 Li, H. *et al.* The Sequence Alignment/Map format and SAMtools. *Bioinformatics* **25**, 2078-2079, doi:10.1093/bioinformatics/btp352 (2009).

16 Ramirez, F. *et al.* deepTools2: a next generation web server for deep-sequencing data analysis. *Nucleic Acids Res* **44**, W160-165, doi:10.1093/nar/gkw257 (2016).

17 Uren, P. J. *et al.* Site identification in high-throughput RNA-protein interaction data. *Bioinformatics* **28**, 3013-3020, doi:10.1093/bioinformatics/bts569 (2012).

18 Yeo, G. & Burge, C. B. Maximum entropy modeling of short sequence motifs with applications to RNA splicing signals. *J Comput Biol* **11**, 377-394, doi:10.1089/1066527041410418 (2004).

19 RCoreTeam. R: A language and environment for statistical computing. R Foundation for Statistical Computing, Vienna, Austria. https://www.R-project.org/ (2018).

20 Young, M. D., Wakefield, M. J., Smyth, G. K. & Oshlack, A. Gene ontology analysis for RNA-seq: accounting for selection bias. *Genome Biol* **11**, R14, doi:10.1186/gb-2010-11-2-r14 (2010).

21 Liao, Y., Smyth, G. K. & Shi, W. The Subread aligner: fast, accurate and scalable read mapping by seed-and-vote. *Nucleic Acids Res* **41**, e108, doi:10.1093/nar/gkt214 (2013).

22 Robinson, M. D., McCarthy, D. J. & Smyth, G. K. edgeR: a Bioconductor package for differential expression analysis of digital gene expression data. *Bioinformatics* **26**, 139-140, doi:10.1093/bioinformatics/btp616 (2010).

23 Shen, L., Shao, N., Liu, X. & Nestler, E. ngs.plot: Quick mining and visualization of next-generation sequencing data by integrating genomic databases. *BMC Genomics* **15**, 284, doi:10.1186/1471-2164-15-284 (2014).

24 Robinson, J. T. *et al.* Integrative genomics viewer. *Nat Biotechnol* **29**, 24-26, doi:10.1038/nbt.1754 (2011).
