# Supplementary material for: A transcriptome-wide antitermination mechanism sustaining identity of embryonic stem cells
Source: Nat Commun. 2020 Jan 17;11:361. doi: 10.1038/s41467-019-14204-z (PMC6969169; doi:10.1038/s41467-019-14204-z)
Supplement: Supplementary file 3 — Reporting Summary [file 41467_2019_14204_MOESM3_ESM.pdf]

nature research

Corresponding author(s): Eugene Makeyev

Last updated by author(s): November 28, 2019

Reporting Summary

Nature Research wishes to improve the reproducibility of the work that we publish. This form provides structure for consistency and transparency in reporting. For further information on Nature Research policies, see Authors & Referees and the Editorial Policy Checklist.

Statistics

For all statistical analyses, confirm that the following items are present in the figure legend, table legend, main text, or Methods section.

n/a	Confirmed	
☐	☒	The exact sample size (*n*) for each experimental group/condition, given as a discrete number and unit of measurement
☐	☒	A statement on whether measurements were taken from distinct samples or whether the same sample was measured repeatedly
☐	☒	The statistical test(s) used AND whether they are one- or two-sided<br>*Only common tests should be described solely by name; describe more complex techniques in the Methods section.*
☒	☐	A description of all covariates tested
☐	☒	A description of any assumptions or corrections, such as tests of normality and adjustment for multiple comparisons
☐	☒	A full description of the statistical parameters including central tendency (e.g. means) or other basic estimates (e.g. regression coefficient) AND variation (e.g. standard deviation) or associated estimates of uncertainty (e.g. confidence intervals)
☐	☒	For null hypothesis testing, the test statistic (e.g. *F*, *t*, *r*) with confidence intervals, effect sizes, degrees of freedom and *P* value noted<br>*Give P values as exact values whenever suitable.*
☒	☐	For Bayesian analysis, information on the choice of priors and Markov chain Monte Carlo settings
☒	☐	For hierarchical and complex designs, identification of the appropriate level for tests and full reporting of outcomes
☒	☐	Estimates of effect sizes (e.g. Cohen's *d*, Pearson's *r*), indicating how they were calculated

Our web collection on statistics for biologists contains articles on many of the points above.

Software and code

Policy information about availability of computer code

Data collection	Computer code used in this study is described in the Methods and Supplemental Data 5.<br><br>The main programs that we used were:<br>- LightCycler 96 software (Version 1.1.0.1320); Roche<br>- Image Studio Lite (Version 5.2); LI-COR Biosciences; https://www.licor.com/bio/image-studio-lite/
Data analysis	Computer code used in this study is described in the Methods and Table S5.<br><br>The main programs that we used were:<br>- Bowtie2 (Version 2.2.6); (Langmead and Salzberg, 2012); http://bowtie-bio.sourceforge.net/bowtie2/index.shtml<br>- HISAT2 (Version 2.1.0); (Kim et al., 2015); http://ccb.jhu.edu/software/hisat2/index.shtml<br>- Bedtools (Version 2.25.0); (Quinlan and Hall, 2010); http://bedtools.readthedocs.io/en/latest/<br>- SAMtools (Version 1.6); (Li et al., 2009); http://www.htslib.org/<br>- Deeptools (Version 3.1.3); (Ramirez et al., 2016); https://deeptools.readthedocs.io/en/develop/<br>- Piranha (Version 1.2.1); (Uren et al., 2012); http://smithlabresearch.org/software/piranha/<br>- MaxEntScan::score5ss ; (Yeo and Burge, 2004); http://hollywood.mit.edu/burgelab/maxent/Xmaxentscan_scoreseq.html<br>- R (Version 3.2.3); (RCoreTeam, 2018); https://www.r-project.org/<br>- goseq (Version 1.22.0); (Young et al., 2010); https://bioconductor.org/packages/release/bioc/html/goseq.html<br>- Rsubread (Version 1.20.6); (Liao et al., 2013); https://bioconductor.org/packages/release/bioc/html/Rsubread.html<br>- edgeR (Version 3.12.1); (Robinson et al., 2010); https://www.bioconductor.org/packages/release/bioc/html/edgeR.html<br>- ngs.plot (Version 2.61); (Shen et al., 2014); https://github.com/shenlab-sinai/ngsplot<br>- IGV (Version 2.4); (Robinson et al., 2011); https://software.broadinstitute.org/software/igv/download

For manuscripts utilizing custom algorithms or software that are central to the research but not yet described in published literature, software must be made available to editors/reviewers. We strongly encourage code deposition in a community repository (e.g. GitHub). See the Nature Research guidelines for submitting code & software for further information.

Data

Policy information about availability of data

All manuscripts must include a data availability statement. This statement should provide the following information, where applicable:

- Accession codes, unique identifiers, or web links for publicly available datasets
- A list of figures that have associated raw data
- A description of any restrictions on data availability

The RNA-Seq, 3'RNA-Seq and RAP-Seq data generated in this study are available from ArrayExpress (E-MTAB-7626, E-MTAB-7635). Publicly available sequencing data used in our study are summarized in Supplemental Data 5. Raw data are provided as the Source Data file

Field-specific reporting

Please select the one below that is the best fit for your research. If you are not sure, read the appropriate sections before making your selection.

☒ Life sciences ☐ Behavioural & social sciences ☐ Ecological, evolutionary & environmental sciences

For a reference copy of the document with all sections, see nature.com/documents/nr-reporting-summary-flat.pdf

Life sciences study design

All studies must disclose on these points even when the disclosure is negative.

Sample size	No sample size calculation was performed
Data exclusions	No data exclusions
Replication	Experiments were replicated independently at least twice (samples for high-throughput sequencing) and typically 3 or a larger number of times as indicated in the text
Randomization	We did not randomize samples
Blinding	Non-blinded design was used

Reporting for specific materials, systems and methods

We require information from authors about some types of materials, experimental systems and methods used in many studies. Here, indicate whether each material, system or method listed is relevant to your study. If you are not sure if a list item applies to your research, read the appropriate section before selecting a response.

Materials & experimental systems

n/a	Involved in the study	
☐	☒	Antibodies
☐	☒	Eukaryotic cell lines
☒	☐	Palaeontology
☒	☐	Animals and other organisms
☒	☐	Human research participants
☒	☐	Clinical data

Methods

n/a	Involved in the study	
☒	☐	ChIP-seq
☐	☒	Flow cytometry
☒	☐	MRI-based neuroimaging

Antibodies

Antibodies used

-anti-Srrt/Ars2; Abcam; Cat# ab192999
-anti-Ncbp1; Abcam; Cat# ab42389
-anti-Pou5f1/Oct4; Abcam; Cat# ab19857
-anti-Sox2; Abcam; Cat# ab97959
-anti-Nanog; Thermo Fisher Scientific; Cat# 14-5761-80
-anti-Snrpa/U1-A; Proteintech; Cat# 10212-1-AP
-anti-Snrp70/U1-70K; Gift from T. Maniatis (mouse mAb); NA
-anti-Erk1/2; Cell Signalling Technology; Cat# 9102
-anti-Gapdh; Thermo Fisher Scientific; Cat# AM4300
-anti-Pecam1/CD31, APC conjugated; Thermo Fisher Scientific; Cat# 17-0311-80
-anti-SSEA1, Alexa Fluor 488-conjugated; Thermo Fisher Scientific; Cat# 53-8813-41
-Peroxidase-AffiniPure Goat Anti-Rabbit IgG ; Jackson Immunoresearch ; Cat# 111-035-045-JIR-1.5ml
-Peroxidase-AffiniPure Goat Anti-Mouse IgG; Jackson Immunoresearch; Cat# 115-035-062-JIR-1.5ml

	-IRDye 800CW goat anti-mouse IgG (H+L); Li-COR Biosciences; Cat# 925-32210<br>-IRDye 680RD goat anti-rabbit IgG (H+L); Li-COR Biosciences; Cat# 926-68071
Validation	All antibodies were used for species and applications recommended by the manufacturers. Specificity of the Srrt- and the Ncbp1-specific antibodies was additionally confirmed by RNAi (Fig. 4d).

Eukaryotic cell lines

Policy information about cell lines

Cell line source(s)	Mouse embryonic stem cell line A2Lox (ES-E14TG2a derivative) was a gift from Michael Kyba [Iacovino, M., Bosnakovski, D., Fey, H., Rux, D., Bajwa, G., Mahen, E., Mitanoska, A., Xu, Z., and Kyba, M. Inducible cassette exchange: a rapid and efficient system enabling conditional gene expression in embryonic stem and primary cells. Stem Cells 29, 1580-1588 (2011)].
Authentication	A2Lox cells were authenticated based on the colony morphology in 2i medium, resistance to puromycin, expression of pluripotency factors Oct4, Sox2 and Nanog and the ability of cells to undergo recombination-mediated cassette exchange reaction in a Dox-inducible manner forming G418-resistant transgenic colonies.
Mycoplasma contamination	A2Lox cells were mycoplasma-negative
Commonly misidentified lines<br>(See ICLAC register)	N/A

Flow Cytometry

Plots

Confirm that:

☒ The axis labels state the marker and fluorochrome used (e.g. CD4-FITC).

☒ The axis scales are clearly visible. Include numbers along axes only for bottom left plot of group (a 'group' is an analysis of identical markers).

☒ All plots are contour plots with outliers or pseudocolor plots.

☒ A numerical value for number of cells or percentage (with statistics) is provided.

Methodology

Sample preparation	A2Lox mouse ESCs transfected with siRNAs in a 12-well plate format were incubated in 2i medium for 48 hours, dissociated using Accutase (Thermo Fisher Scientific, cat# A1110501), washed with 1×PBS, pH 7.4 (Thermo Fischer Scientific, cat# 10010023), and resuspended in the 100 µl of FACS buffer containing 1×PBS, 2 mM EDTA and 3% FBS. Cells were then stained for ESC surface markers using an APC-conjugated anti-Pecam1/CD31 antibody (Thermo Fisher Scientific, cat# 17-0311-80, 0.5µg per test) and an Alexa Fluor 488-conjugated anti-SSEA1 antibody (Thermo Fisher Scientific, cat# 53-8813-41, 0.125µg per test) for 1 hour on ice, washed twice with 300µl of the FACS buffer and passed through Falcon 40 µm cell strainers to obtain a single-cell suspensions. Samples were supplemented with 0.2 µg/ml DAPI ~10 min prior to flow cytometry to label membrane-compromised cells.
Instrument	BD FACSCanto™ II flow cytometer equipped with 405 nm, 488nm and 633 nm lasers
Software	The FCS files were analyzed using the flowCore and the flowViz packages (https://www.bioconductor.org/packages/release/bioc/html/flowCore.html; https://www.bioconductor.org/packages/release/bioc/html/flowViz.html)
Cell population abundance	No cell sorting was performed. Forward and side scatter amplitude and width, as well as DAPI staining were used to exclude dead cells. >28,000 cell passed these gates in each sample. Positive populations were defined using mock-stained cells as a negative control.
Gating strategy	Forward and side scatter amplitude and width, as well as DAPI staining were used to exclude dead cells. The following rectangular boundaries were used to include single living cells:<br>FSC.A: 60000, 140000;<br>SSC.A: 20000, 130000;<br>SSC.W: 80000, 160000;<br>DAPI.A: -100, 5000.<br>>28,000 cell passed these gates in each sample.

☒ Tick this box to confirm that a figure exemplifying the gating strategy is provided in the Supplementary Information.
