# Supplementary material for: A transcriptome-wide antitermination mechanism sustaining identity of embryonic stem cells
Source: Nat Commun. 2020 Jan 17;11:361. doi: 10.1038/s41467-019-14204-z (PMC6969169; doi:10.1038/s41467-019-14204-z)
Supplement: Supplementary file 4 — Description of Additional Supplementary Files [file 41467_2019_14204_MOESM4_ESM.docx]

**Description of Additional Supplementary Files**

File name: Supplementary Data 1
Description: List of genes monotonically downregulated during both spontaneous and neuronal differentiation of mouse ESCs

File name: Supplementary Data 2
Description: Genes significantly regulated in response to siSrrt

File name: Supplementary Data 3
Description: Biological process GO terms significantly over-represented among siSrrt-upregulated genes (FC≥1.5, FDR<0.05)

File name: Supplementary Data 4
Description: siSrrt-induced upregulation of CSs in first introns is associated with gene downregulation

File name: Supplementary Data 5
Description: Key resources used in this study

File name: Supplementary Data 6
Description: Plasmids generated in this study

File name: Supplementary Data 7
Description: DNA oligonucleotides used in this study
