# Supplementary material for: A transcriptome-wide antitermination mechanism sustaining identity of embryonic stem cells
Source: Nat Commun. 2020 Jan 17;11:361. doi: 10.1038/s41467-019-14204-z (PMC6969169; doi:10.1038/s41467-019-14204-z)
Supplement: Supplementary file 9 — Supplementary Data 5 [file 41467_2019_14204_MOESM9_ESM.docx]

**Supplementary Data 5**. Key resources used in this study

| **Reagent or Resource** | **Source** | **Identifier** |
| --- | --- | --- |
| **Antibodies (working dilutions)** | | |
| anti-Srrt/Ars2 (Immunoblotting, 1:3000; co-IP, 5 μg per reaction) | Abcam | Cat# ab192999 |
| anti-Ncbp1 (Immunoblotting, 1:3000; RIP, 5 μg per reaction) | Abcam | Cat# ab42389 |
| Non-immune rabbit IgG control (co-IP, 5 μg per reaction; RIP, 5 μg per reaction) | Thermo Fisher Scientific | Cat# 10500C |
| anti-Pou5f1/Oct4 (Immunoblotting, 1:1000) | Abcam | Cat# ab19857 |
| anti-Sox2 (Immunoblotting, 1:1000) | Abcam | Cat# ab97959 |
| anti-Nanog (Immunoblotting, 1:500) | Thermo Fisher Scientific | Cat# 14-5761-80 |
| anti-Snrpa/U1-A (Immunoblotting, 1:1000) | Proteintech | Cat# 10212-1-AP |
| anti-Snrp70/U1-70K (Immunoblotting, 1:1000) | Gift from T. Maniatis (mouse mAb) | NA |
| anti-Erk1/2 (Immunoblotting, 1:1000) | Cell Signalling Technology | Cat# 9102 |
| anti-Gapdh (Immunoblotting, 1:5000) | Thermo Fisher Scientific | Cat# AM4300 |
| anti-Pecam1/CD31, APC conjugated (Flow cytometry, 0.5 μg per 100 μl cell suspension) | Thermo Fisher Scientific | Cat# 17-0311-80 |
| anti-SSEA1, Alexa Fluor 488 conjugated (Flow cytometry, 0.125 μg per 100 μl cell suspension) | Thermo Fisher Scientific | Cat# 53-8813-41 |
| Peroxidase-AffiniPure Goat Anti-Rabbit IgG  (Immunoblotting, 1:10000 to 1:20000) | Jackson Immunoresearch | Cat# 111-035-045-JIR-1.5ml |
| Peroxidase-AffiniPure Goat Anti-Mouse IgG  (Immunoblotting, 1:10000 to 1:20000) | Jackson Immunoresearch | Cat# 115-035-062-JIR-1.5ml |
| IRDye 800CW goat anti-mouse IgG (H+L) (Immunoblotting, 1:10000) | Li-COR Biosciences | Cat# 925-32210 |
| IRDye 680RD goat anti-rabbit IgG (H+L) (Immunoblotting, 1:10000) | Li-COR Biosciences | Cat# 926-68071 |
| **Oligonucleotides** | | |
| ON-TARGET plus non-targeting siRNA (siControl) | Horizon Discovery | Cat# D-001810-01-20 |
| Mouse Srrt-specific ON-TARGETplus, siRNA SMARTpool | Horizon Discovery | Cat# LQ-045428-02-0002 |
| Mouse Srrt-specific ON-TARGETplus, individual siRNA (#1) | Horizon Discovery | Cat# J-045428-17-0010 |
| Mouse Srrt-specific ON-TARGETplus, individual siRNA (#2) | Horizon Discovery | Cat# J-045428-18-0010 |
| Mouse Srrt-specific ON-TARGETplus, individual siRNA (#3) | Horizon Discovery | Cat# J-045428-19-0010 |
| Mouse Srrt-specific ON-TARGETplus, individual siRNA (#4) | Horizon Discovery | Cat# J-045428-20-0010 |
| Mouse Ammecr1-specific ON-TARGETplus, siRNA SMARTpool | Horizon Discovery | Cat# L-059312-01-0005 |
| Mouse Ncbp1-specific ON-TARGETplus, siRNA SMARTpool | Horizon Discovery | Cat# LQ-160309-00-0002 |
| Mouse Exosc3-specific ON-TARGETplus, siRNA SMARTpool | Horizon Discovery | Cat# L-064537-01-0005 |
| Mouse Exosc10-specific ON-TARGETplus, siRNA SMARTpool | Horizon Discovery | Cat# L-049286-00-0005 |
| Mouse Dis3-specific ON-TARGETplus, siRNA SMARTpool | Horizon Discovery | Cat# L-048884-01-0005 |
| Control antisense morpholino oligonucleotide: 5’-CCTCTTACCTCAGTTACAATTTATA-3’ | Gene Tools, LLC | N/A |
| U1 antisense morpholino oligonucleotide: 5’-GGTATCTCCCCTGCCAGGTAAGTAT-3’ | Gene Tools, LLC | N/A |
| U2 antisense morpholino oligonucleotide: 5’-TGATAAGAACAGATACTACACTTGA-3’ | Gene Tools, LLC | N/A |
| Edit-R CRISPR-Cas9 Synthetic tracrRNA | Horizon Discovery | Cat# U-002005-05 |
| Edit-R crRNA Non-targeting Control #1 | Horizon Discovery | Cat# U-007501-01-05 |
| Edit-R crRNA Non-targeting Control #2 | Horizon Discovery | Cat# U-007502-01-05 |
| Edit-R modified synthetic crRNA targeting the first intron of mouse *Ammecr1* gene:  mU.*.mG.*.U.C.G.G.A.C.U.C.C.G.C.U.G.U.G.C.U.C.G.U.U.U.U.A.G.A.G.C.U.A.U.G.C.U.G.U.U.U.U.G | Horizon Discovery | Cat# crRNA-343745 GEHCU-004465 |
| Edit-R modified synthetic crRNA targeting the first intron of mouse *Ammecr1* gene:  mA.*.mG.*.C.U.C.U.U.G.G.G.C.A.G.A.A.G.G.G.A.U.G.U.U.U.U.A.G.A.G.C.U.A.U.G.C.U.G.U.U.U.U.G | Horizon Discovery | Cat# crRNA-343745 GEHCU-004466 |
| Assorted DNA oligonucleotides | This study/IDT | See Supplementary Data 7 |
| **Recombinant DNA** | | |
| Plasmid p2Lox | Supplementary Ref^10^; Addgene | RRID:Addgene_34635 |
| Plasmid pEGFP-N3 | Clontech | N/A |
| Plasmid pX330-U6-Chimeric_BB-CBh-hSpCas9 | Supplementary Ref^11^; Addgene | RRID:Addgene_42230 |
| Plasmid pGEM-T easy | Promega | A1360 |
| Plasmid pCR-bluntII-topo-hSRRT-ORF | Horizon Discovery | Clone ID: 40035609 cat# MHS6278-211690300 |
| Plasmid pML407 (p2Lox derivative for inducible overexpression of human SRRT) | This study | See Supplementary Data 6 |
| Plasmid pML412 (p2Lox derivative for inducible overexpression of spCas9) | This study | See Supplementary Data 6 |
| Plasmid pML171 (Intermediate cloning construct) | This study | See Supplementary Data S6 |
| Plasmid pML408 (pML171 derivative with Ammecr1 minigene containing 5’ss of the first intron and intronic cleavage site) | This study | See Supplementary Data S6 |
| Plasmid pML409 (pML408 with deleted PAS sequences) | This study | See Supplementary Data 6 |
| Plasmid pML410 (pML408 with mutated 5’ss sequence) | This study | See Supplementary Data 6 |
| Plasmid pML411 (pML410 with mutated U1-like binding intronic sequences) | This study | See Supplementary Data 6 |
| Plasmid pML475 (plasmid used to generate a mouse U1 snRNA-specific Northern blot probe) | This study | See Supplementary Data 6 |
| **Deposited Data** | | |
| Single cell RNA-Seq analysis of mouse ES cells cultured with serum | Supplementary Ref^2^ | GEO accession: GSE75804 |
| RNA-Seq analysis of longitudinal changes during neurogenesis and neuronal maturation | Supplementary Ref^6^ | SRA Bioproject accession: PRJNA185305 |
| RNA-Seq comparisons of A2lox treated with control or siRNA against Srrt or Ncbp1 | This study | ArrayExpress accession:  E-MTAB-7626 |
| 3’-End sequencing (QuantSeq 3’ mRNA-Seq) comparisons of A2lox treated with control or siRNA against Srrt or Ncbp1 | This study | ArrayExpress accession:  E-MTAB-7626 |
| RAP-Seq comparison of A2lox treated with control or siRNA against Srrt | This study | ArrayExpress accession:  E-MTAB-7635 |
| 3’-End sequencing (2P-seq) in Exosc3 conditional KO mESCs | Supplementary Ref^8^ | GEO accession: GSE100536 |
| RNA-Seq analysis of Dicer1 KO mESCs | Supplementary Ref^9^ | GEO accession: GSE55338 |
| **Software** | | |
| Bowtie2 (Version 2.2.6) | Supplementary Ref^12^;  http://bowtie-bio.sourceforge.net/bowtie2/index.shtml | RRID:SCR_016368 |
| HISAT2 (Version 2.1.0) | Supplementary Ref^13^; http://ccb.jhu.edu/software/hisat2/index.shtml | RRID:SCR_015530 |
| Bedtools (Version 2.25.0) | Supplementary Ref^14^; http://bedtools.readthedocs.io/en/latest/ | RRID:SCR_006646 |
| SAMtools (Version 1.6) | Supplementary Ref^15^; http://www.htslib.org/ | RRID:SCR_002105 |
| Deeptools (Version 3.1.3) | Supplementary Ref^16^; https://deeptools.readthedocs.io/en/develop/ | RRID:SCR_016366 |
| Piranha (Version 1.2.1) | Supplementary Ref^17^; http://smithlabresearch.org/software/piranha/ | RRID:SCR_010903 |
| MaxEntScan::score5ss | Supplementary Ref^18^; http://hollywood.mit.edu/burgelab/maxent/Xmaxentscan_scoreseq.html | RRID:SCR_016707 |
| R (Version 3.2.3) | Supplementary Ref^19^; https://www.r-project.org/ | RRID:SCR_001905 |
| goseq (Version 1.22.0) | Supplementary Ref^20^; https://bioconductor.org/packages/release/bioc/html/goseq.html | RRID:SCR_017052 |
| Rsubread (Version 1.20.6) | Supplementary Ref^21^; https://bioconductor.org/packages/release/bioc/html/Rsubread.html | RRID:SCR_009803 |
| edgeR (Version 3.12.1) | Supplementary Ref^22^; https://www.bioconductor.org/packages/release/bioc/html/edgeR.html | RRID:SCR_012802 |
| pheatmap (Version 1.0.12) | https://cran.r-project.org/web/packages/pheatmap/ | RRID:SCR_016418 |
| VennDiagram (Version 1.6.18) | http://cran.r-project.org/web/packages/VennDiagram/ | RRID:SCR_002414 |
| flowCore (Version 1.50.0) | https://www.bioconductor.org/packages/release/bioc/html/flowCore.html | RRID:SCR_002205 |
| flowViz (Version 1.48.0) | https://www.bioconductor.org/packages/release/bioc/html/flowViz.html | RRID:SCR_002075 |
| ngs.plot (Version 2.61) | Supplementary Ref^23^; https://github.com/shenlab-sinai/ngsplot | RRID:SCR_011795 |
| IGV (Version 2.4) | Supplementary Ref^24^; https://software.broadinstitute.org/software/igv/download | RRID:SCR_011793 |
| ImageJ (Version 1.52o) | https://imagej.nih.gov/ij/ | RRID:SCR_003070 |
| Image Studio Lite (Version 5.2) | LI-COR Biosciences; https://www.licor.com/bio/image-studio-lite/ | RRID:SCR_013715 |
| LightCycler 96 software (Version 1.1.0.1320) | Roche | N/A |
