# Supplementary material for: A transcriptome-wide antitermination mechanism sustaining identity of embryonic stem cells
Source: Nat Commun. 2020 Jan 17;11:361. doi: 10.1038/s41467-019-14204-z (PMC6969169; doi:10.1038/s41467-019-14204-z)
Supplement: Supplementary file 2 — Peer Review [file 41467_2019_14204_MOESM2_ESM.pdf]

Reviewers' comments:

Reviewer #1 (Remarks to the Author):

Kainov et al studied the role of Srrt/Ars2 in mouse embryonic stem cells (mESCs). Using RNA-seq and 3'end poly(A) RNA-seq, the authors showed that Ars2 knockdown in mESCs caused an increase of premature mRNAs utilizing poly(A) sites within the first intron, and a decrease of the corresponding mature full-length mRNA. Ars2 knockdown also decreases U1 snRNP occupancy upstream of those intronic poly(A) sites, measured using U1 snRNA pulldown and sequencing. The authors concluded that Ars2 help maintain ESC identity by recruiting U1 snRNP to suppress intronic polyadenylation-mediated truncation of genes important for ESC identity.

This is a very comprehensive study. However, as the authors have briefly discussed, the main observation that Ars2 suppresses intronic polyadenylation directly contradicts a previous study (ref 11) that showed Ars2 promotes intronic polyadenylation. Although the authors speculated possible explanations, no data is presented. In an effort to reconcile the two studies, I found that further analysis will be needed to exclude an alternativ hypothesis, that the observation here is simply a secondary effect Ars2's role in U1 snRNA processing/maturation, as reported in the previous study (ref 11). In that study, Hallais et al reported that in HeLa cells ARS2 (in a complex with the Cap-binding complex CBC) stimulates both the 3' end maturation of snRNAs (including U1) and premature polyadenylation of mRNAs, thus promoting both directions in telescripting (i.e. U1 and premature polyadenylation). The outcome of Ars2 knockdown thus depends on which activity is more limiting. It's possible in mESCs U1 is limiting, thus Ars2 knockdown resembles U1 inhibition phenotype.

Other comments:
1. siRNA off-targets. It appears Ars2 knockdown is based on a single siRNA. The authors need to show the effect is specific by using another siRNA. Or a Sylamer type analysis showing that downregulated genes do not enrich in their 3' UTR seed-match of the siRNA.
2. The Key Resources Table is missing, which contains important information such as siRNA/antisense oligo sequences
3. Another puzzling observation is that given the low abundance of Ars2 in neurons, these cells should have more intronic polyadenylation events and shorter 3' UTRs (if Ars2 suppresses intornic polyadenylation), which is the opposite of what we know (neurons have long 3' UTRs). Again this may suggest in neurons U1 is no longer limiting.

Reviewer #2 (Remarks to the Author):

In the manuscript, Kainov and Makeyev investigated the role of Srrt/Ars2, a component of the nuclear cap-binding complex (CBC), in maintenance maintaining the pluripotent state of mouse embryonic stem cells (ESCs). They show that ESC cells have relatively high levels of Srrt protein and its knockdown promotes their differentiation. Transcriptome sequencing revealed that this is associated with early transcription termination in numerous genes from cleavage and polyadenylation (CPA) at cryptic polyadenylation signals (PAS) in the first intron proximal to the 5'end, where the CBC binds. Previous studies have described similar role for U1 snRNP in PAS suppression, called telescripting, which is necessary for full-length transcription in most mammalian genes. Crosslinking experiments demonstrate an interaction between Srrt and U1 snRNP, further linking Srrt to telescripting. The authors conclude that telescripting plays an important role in pluropotency of ESCs and thereby the transition to differentiated state.
The observations relating Srrt levels and telescripting to key decisions of ESCs are interesting. However, of the other main points have been previously reported, including cap-proximal loss of RNAs due to Srrt knockdown, and the interaction between the CBC and U1 snRNP, which is crucial for binding to the first 5' splice site. Thus, the level of novelty represented by this manuscript for Nature

Communications is questionable. Addressing the points below should help the authors further their work.

Significant issues:
1. The claim that the anti-termination activity of Srrt is the cause of loss of identity of ESCs is problematic because Srrt knockdown causes many types of transcriptome changes.
2. In Figure 2C and 4C, there seem to be a number of genes that show intronic CPA but are also up-regulated with siSrrt and siNcbp1. Are these not contradictory outcomes? This is not mentioned in the text and raises a major concern about how expression level is calculated and the conclusions that are based on it. It appears that the up-regulation applies only to the first exon, despite down-regulation throughout the rest of the gene.
3. In Figure 2D, is there any information about already reported PASs in the introns of these example genes? Are these genes ESC-specific or proliferation markers? Is the early termination not a general effect beyond these genes?
4. In Figure 2D and throughout, it is necessary to show the RNA-seq reads for the entire gene, or at least a much larger region than just the cap-proximal map
5. In Figure S6G, siSrrt does not show any change in precursor U1 snRNA level, however this is not a direct evidence of U1 snRNP level changes. Are U1 snRNP protein amounts changed by siSrrt?
6. The relevance of the GO terms of genes that are up-regulated by siSrrt and those that are down-regulated to the biological phenomenon must be discussed and explained. How do the functions of the affected genes explain proliferation vs differentiation – it doesn't look like it to me, which reinforces the concern that there is something fundamentally wrong about the data analysis.

Minor comments:
1. In Figure 1E and 2E, the location of the primers used in these experiments within the gene should be indicated.
2. In Figure 2D, the 3'RACE results should be shown first to validate these products as being from CPA.
3. In Figure 2E and 3D-E, using "downstream" and "upstream" instead of "before" and "after" is more correct, and easier to understand, nomenclature.
4. An effort should be made to explain, in the text, how gene expression was calculated (ex. from which gene regions).

Reviewer #3 (Remarks to the Author):

In this study by Kainov and Makeyev, the authors explore the role of the BCB co-factor protein Srrt in ESCs. They find that knockdown of Srrt leads to differentiation of ESCs, and the premature introduction of new 3' ends and poly-adenylation sites and premature transcription termination sites for a subset of transcripts. These new 3' ends are mainly localised inside retrotransposons, and particularly SINEs. I find the study to be mostly well supported, interesting, novel, and suitable for publication in Nature communications. I have a few major comments that the authors should address before the manuscript is ready.

Major points:
1. The claims that Srrt1 knockdown causes differentiation are not convincing. However, in my opinion it is not essential for the paper that this be true, indeed, the lack of robust differentiation and deregulation of Sox2 is even helpful for the authors as it means that the mechanism is different from NSCs.

The authors may prefer to redevelop this part of the manuscript, in which case I have given some

advice below on how they can make this claim more robust. Alternatively, they can delete this part of the manuscript or substantially tone down their claims about differentiation. Dispensing with the claim that Srrt is required for pluripotency, would, in my opinion, still make the manuscript interesting to a wide readership.

The overall number of differentially regulated genes is quite small for a differentiation process (Fig. S1C). I suspect that techniques like PCA would indicate that the SrrtKD cells are still close to ESCs. It would be useful to reanalyze the data from Refs 39 and 40 and integrate them with the siCtrl and siSrrt data sets to observe the differentiation of the cells. A pair-wise co-correlation, and/or PCA of the RNA-seq samples in this study versus ref 39 and 40 would help understand the nature of the cells differentiation.

Figure 1E: The AP staining changes is not convincing for differentiation, particularly in light of Fig 1G. The authors might be better off with SSEA1 staining which is more sensitive for pluripotency. However, as Nanog is not downregulated (Fig. 1G), it seems more likely the cells are not strongly differentiating.

Fig 1F: The expression of a wider panel of pluripotency genes should be analysed, taken from the RNA-seq data. Also include 2i and serum+LIF genes. A few heatmaps would be helpful here to support this claim.

Fig. S1E: Should be a Venn diagram, also showing the number of genes DE in the spontaneously differentiating cells.

A wider panel of somatic-specific genes should be analysed. For example the collagens, other genes involved in somatic state, Jun, Ets-family TFs, also the naive and ground state TFs, EpiSC and early gastrulation genes etc.

Cell surface genes, and particularly the epithelial/mesenchymal state of the cells should be shown. From the AP staining (Fig 1E) the cells appear to be mesenchymal like, and simultaneously pluripotent (a few examples of this phenomena have been published). Expression of the cadherins should be explored. I notice that Pecam1 is down-regulated.

Fig 1F: The presence of Lefty1 and Lefty2 is strange here, as they should be extremely low or even absent as the cells were cultured in 2i medium, and so being down-regulated further should be very difficult. Similarly, these two genes are early markers for differentiation and would be expected to go up if the cells were differentiating. For example, both genes are robustly expressed in EpiSCs and should be unregulated in the authors spontaneously differentiated cells.

Apart from this, I only have a few other comments on the rest of the manuscript, which is convincing, well prepared and analysed.

2. Fig 4B: The manuscript says that "…analyses revealed a strong correlation between the siNcbp1- and the siSrrt-treated samples…". But the figure shows a Pearson r of 0.74, which is actually a pretty low correlation when comparing all genes in two samples. It suggests that the Ncbp1 and Srrt KDs are different. The authors could address this by showing that the Srrt is a subset of the Ncbp KD data using a Venn (this seems quite likely), or explain more clearly why they think these samples are similar.

I would also be interested in seeing the PCA for Srrt, Ncbp1 KDs versus the controls and the differentiated ESC samples used in this study.

3. Page 16: "The number of genes that require Srrt for their normal expression in ESCs approaches the size of gene regulation networks controlled by Pou5f1, Nanog, and Sox2 [Tables S2 and S3; 38].

This may explain, at least in part, why Srrt is indispensable for preimplantation development of mouse embryos". I find this argument a bit odd. Down-regulation of any of Pou5f1, Sox2 or Nanog causes very rapid changes in gene expression, as thousands of genes are deregulated, and measuring the size of gene regulation networks is a very arbitrary procedure, prone to difficulty. Ref 38 is an odd choice here, as it barely mentions gene expression networks. Whilst the best characterized network study in pluripotency (Dunn SJ, EMBO J 2018) is not cited. I suggest delete this comment, which seems to be trying to indirectly link Srrt to pluripotency regulation.

Minor comments:
In addition, I have a few minor comments which appear to be errors, or are unclear:

Fig 3C: The figure panel and figure legend says 'RT-qPCR Srrt', but the text says it is RT-qPCR for Ammerc1? Appears to be a figure labelling mistake.

Fig 2E and 3C: The magnitude of effect in this experiment is quite different, even though the WT part is the same experiment as in Figure 2E.

Fig S6E, F: Surely these should include the matching plots for the siSrrt?

Reviewer #4 (Remarks to the Author):

In the manuscript titled "A transcriptome-wide antitermination mechanism sustaining identity of embryonic stem cell", Kainov and Makeyev et al dissected the function of an RNA binding protein Srrt (also known as Ars2) in embryonic stem cells. They found that knocking down Srrt led to premature termination of hundreds of transcripts at cryptic cleavage/polyadenylation sites. Mechanistically, SRRT protein may promote the recruitment of U1 snRNP to prevent premature termination at normal ESCs through CBCs. Finally, they found that SRRT regulated cleavage/polyadenylation sites in first intron enriched for retrotransposable elements. Based on their experiments, they made conclusion that Srrt is a molecular guardian of pluripotent state that likely played a profound role in evolution of mammalian genome. The manuscript is well written. The finding of Srrt inhibiting premature termination is novel. I support the publication of the manuscript after a few major concerns are addressed:

1. The claim that Srrt sustains the identity of embryonic stem cells is only based on not so clear decrease of alkaline phosphatase staining. More experiments have to be performed to support this claim. SSEA1 staining, immunofluorescence of pluripotency markers and colony formation assay should be performed. If possible, a reporter cell line (e.g. Rex1-GFP or Oct4-GFP) should be used for siSrrt experiments. Western analysis of OCT4, SOX2 and NANOG instead of RT-qPCR of these genes should be performed.

2. During differentiation, Srrt RNA and protein level are significantly downregulated. If the hypothesis of this study is correct, one would expect that at least some of transcripts altered in siSrrt embryonic stem cells are also prematurely terminated during differentiation or in differentiated cells. The authors need check this. These experiments will also help to clarify whether there is a link between Srrt and the identity of embryonic stem cells.

3. Only one siSrrt is used throughout the manuscript. To exclude possible off-target effects, at least some of experiments need to be repeated with different siRNAs or different knocking down approaches (e.g. CRISPRi).

4. CBC was reported to recruit Srrt. In this study, the authors suggest that Srrt promote U1SnRNP binding through CBC. Does Srrt promote the binding of CBC to related transcripts? RIP using antibodies against CBC components followed by qPCR of related transcripts should be performed in

siCtrl and siSrrt cells. In addition, is there any evidence that SRRT directly interacts with CBC in embryonic stem cells? Is this interaction dependent on RNA?

5. The claim on the role of Srrt in evolution of mammalian genome is rather broad and very significant. Is there any evidence that Srrt plays similar functions in inhibiting RTEs in other mammals, particularly human?

Minor concerns:
1. Gene, RNA and protein names should be standardized. For example, for western, SRRT instead of Srrt should be used.

2. All western should be performed for at least 3 times and quantified. In addition, protein markers should be shown in all panels.

3. "There were 284 genes with intronic CS (iCS) upregulated ≥2-fold, FDR<0.05 and expression level reduced ≥1.5-fold, FDR<0.05, and an even larger number of genes showing this trend was detected when using less stringent cutoffs (Table S3)." in the main text, however, in Figure 2C, 307 genes are labelled.

4. Figure 3D and E, data should be normalized to WT, siCtrl to demonstrate the impact of delta PAS on the expression of Ammecr1.

5. Figure 4B legends, top left should be "top right".

6. Figure 4D, quantification of western is required. In addition, for Srrt overexpression, lane 4 and 5 should be significantly higher than lane 1 and 2. Lane 6 should be lower than lane 4 and 5 due to knocking down of endogenous SRRT. However, by just looking at these western images, the data presented seem not as expected, especially that lane 6 seem to have similar level of SRRT as in lane 4 and 5.

7. Figure 6. Why amoU2 also significantly decreased read through efficiency? In addition, the knockdown efficiency of amoU1 and amoU2 should be checked.

8. "Importantly, these effects were 7 not due to altered expression of the key pluripotency factors Nanog, Pou5f1, and Sox2 (Fig. 1F)." Fig. 1F should be Fig.1G.

9. For Dicer experiment, the expression of key miRNAs should be checked to make sure that miRNA biogenesis is blocked.

10. Titles for supplementary figures should be added.

We thank all four reviewers for their constructive comments that helped us improve the manuscript as specified below.

Reviewer #1

"Kainov et al studied the role of Srrt/Ars2 in mouse embryonic stem cells (mESCs). Using RNA-seq and 3'end poly(A) RNA-seq, the authors showed that Ars2 knockdown in mESCs caused an increase of premature mRNAs utilizing poly(A) sites within the first intron, and a decrease of the corresponding mature full-length mRNA. Ars2 knockdown also decreases U1 snRNP occupancy upstream of those intronic poly(A) sites, measured using U1 snRNA pulldown and sequencing. The authors concluded that Ars2 help maintain ESC identity by recruiting U1 snRNP to suppress intronic polyadenylation-mediated truncation of genes important for ESC identity.

This is a very comprehensive study. However, as the authors have briefly discussed, the main observation that Ars2 suppresses intronic polyadenylation directly contradicts a previous study (ref 11) that showed Ars2 promotes intronic polyadenylation. Although the authors speculated possible explanations, no data is presented. In an effort to reconcile the two studies, I found that further analysis will be needed to exclude an alternativ hypothesis, that the observation here is simply a secondary effect Ars2's role in U1 snRNA processing/maturation, as reported in the previous study (ref 11). In that study, Hallais et al reported that in HeLa cells ARS2 (in a complex with the Cap-binding complex CBC) stimulates both the 3' end maturation of snRNAs (including U1) and premature polyadenylation of mRNAs, thus promoting both directions in telescripting (i.e. U1 and premature polyadenylation). The outcome of Ars2 knockdown thus depends on which activity is more limiting. It's possible in mESCs U1 is limiting, thus Ars2 knockdown resembles U1 inhibition phenotype."

We thank the reviewer for considering our study "very comprehensive". Regarding the comment that "further analysis will be needed to exclude an alternativ hypothesis, that the observation here is simply a secondary effect Ars2's role in U1 snRNA processing/maturation, as reported in the previous study (ref 11)", the original version of the manuscript contained RT-qPCR experiments (currently Fig. S12A) suggesting that Srrt/Ars2 knockdown does not detectably change U1 biogenesis in mouse embryonic stem cells (ESCs).

The following new analyses added to the revised manuscript support our original conclusion.

(i) The newly added Northern blot analyses (Fig. 12B) shows no difference in the amount of mature U1 snRNA between siCtrl- and siSrrt-treated ESCs. There is also no detectable accumulation of longer RNA species in siSrrt samples, an effect that would be expected for defective 3'-terminal processing of U1 precursors.

(ii) As recommended by Reviewer #2, we also analyzed expression levels of U1 snRNP proteins U1-70K and U1-A and found no difference between the siCtrl and the siSrrt samples (new Fig 12C-D).

(iii) Finally, we compared our 3'-terminal RNA-seq data for siSrrt-treated samples with a similar analysis published for mouse ESCs where U1 was inactivated by an antisense morpholino oligonucleotide (amoU1) (new Fig. 12E-F). Although both treatments promoted premature cleavage/polyadenylation in first introns, amoU1 clearly differed from siSrrt by

additionally inducing this effect in other introns on a transcriptome-wide scale. This makes it highly unlikely that siSrrt promotes cleavage/polyadenylation by functionally inactivating U1.

Of note, we do not think that our data directly contradicts Hallais et al. 2013 (Ref 11). The two studies analyzed vastly different cell types potentially expressing distinct Srrt modulators (HeLa cells v mouse ESCs). Another difference is the presence of functional U1 binding sites between the 5'-terminal cap and the iCS in the targets identified in our work but not in the examples investigated by Hallais et al. Moreover, Srrt is naturally "over-expressed" in ESCs and our RNAi-based knockdowns bring its expression down to levels that should be considered physiological in other cell types (Fig. 1B-C and Fig. S1A-B). It is possible that the residual levels of Srrt in siSrrt-treated ESCs are sufficient for U1 snRNP processing but not for the antitermination activity. We discuss some of these possibilities on page 19 of the revised manuscript.

"Other comments:
1. siRNA off-targets. It appears Ars2 knockdown is based on a single siRNA. The authors need to show the effect is specific by using another siRNA. Or a Sylamer type analysis showing that downregulated genes do not enrich in their 3' UTR seed-match of the siRNA."

The original manuscript contained a rescue experiment (Fig. 4D-F) suggesting that premature cleavage/polyadenylation induced by siSrrt, a mixture of 4 Srrt-specific ON-TARGET plus siRNAs, was not an off-target effect. Nonetheless we followed reviewer's suggestion and repeated the key experiments using 3 individual siRNAs, siSrrt#1, siSrrt#2 and siSrrt#3 (new Fig. S6). Gratifyingly, changes in intronic cleavage/polyadenylation and expression of pluripotency/differentiation markers in response to these treatments were very similar to those triggered by siSrrt.

"2. The Key Resources Table is missing, which contains important information such as siRNA/antisense oligo sequences."

As suggested, we added a new table (Table S5) describing key reagents, datasets and software used in our work.

"3. Another puzzling observation is that given the low abundance of Ars2 in neurons, these cells should have more intronic polyadenylation events and shorter 3' UTRs (if Ars2 suppresses intornic polyadenylation), which is the opposite of what we know (neurons have long 3' UTRs). Again this may suggest in neurons U1 is no longer limiting."

Many mRNAs in neurons indeed acquire long 3'UTRs as a result of developmentally regulated transition to cleavage/polyadenylation at more distal sites in the last exon and an increased stability of 3'-extended mRNA species. However, our current study describes a distinct set of targets where regulation occurs in the first intron. This unusual location of iCSs may override the regulation mechanisms controlling the choice between alternative CSs in the last exon.

To check if differentiation of ESCs into neurons can, at least in some cases, stimulate cleavage/polyadenylation in first introns, we inspected published RNA-seq data (Hubbard et al. 2013; Ref 40) for the *Ammecr1* gene (new Fig. S7A-D). Consistent with our model, the overall expression level of *Ammecr1* decreased, whereas the relative abundance of the iCS-terminated transcripts increased in neurons compared to ESCs.

Reviewer #2

"In the manuscript, Kainov and Makeyev investigated the role of Srrt/Ars2, a component of the nuclear cap-binding complex (CBC), in maintenance maintaining the pluripotent state of mouse embryonic stem cells (ESCs). They show that ESC cells have relatively high levels of Srrt protein and its knockdown promotes their differentiation. Transcriptome sequencing revealed that this is associated with early transcription termination in numerous genes from cleavage and polyadenylation (CPA) at cryptic polyadenylation signals (PAS) in the first intron proximal to the 5'end, where the CBC binds. Previous studies have described similar role for U1 snRNP in PAS suppression, called telescripting, which is necessary for full-length transcription in most mammalian genes. Crosslinking experiments demonstrate an interaction between Srrt and U1 snRNP, further linking Srrt to telescripting. The authors conclude that telescripting plays an important role in pluropotency of ESCs and thereby the transition to differentiated state.

The observations relating Srrt levels and telescripting to key decisions of ESCs are interesting. However, of the other main points have been previously reported, including cap-proximal loss of RNAs due to Srrt knockdown, and the interaction between the CBC and U1 snRNP, which is crucial for binding to the first 5' splice site. Thus, the level of novelty represented by this manuscript for Nature Communications is questionable. Addressing the points below should help the authors further their work."

We thank the reviewer for pointing out that the "observations relating Srrt levels and telescripting to key decisions of ESCs are interesting".

However, the reviewer also believes that other "points have been previously reported, including cap-proximal loss of RNAs due to Srrt knockdown, and the interaction between the CBC and U1 snRNP, which is crucial for binding to the first 5' splice site".

The first part of this criticism may refer to the Hallais et al. 2013 study already mentioned in our response to Reviewer #1. Hallais et al. demonstrated that Srrt promotes processing of relatively short RNAs in human cancer cells. What we show in our present work is that Srrt can also inhibit cleavage/polyadenylation at a specific set of cap-proximal CSs in mouse ESCs. This is clearly a distinct molecular activity that constitutes a novel finding. Moreover, to the best of our knowledge, the exact mechanism allowing CBC to recruit U1 (or/and other spliceosomal components) has not been described and its possible contribution to telescripting has been unknown. Our study implicates Srrt as a key player in this molecular circuitry and we are convinced that this finding will be of considerable interest to the field. We introduced several changes to the revised text that should make these points more evident.

"Significant issues:

1. The claim that the anti-termination activity of Srrt is the cause of loss of identity of ESCs is problematic because Srrt knockdown causes many types of transcriptome changes."

The revised manuscript provides three arguments for the importance of the Srrt-dependent antitermination activity for ESCs.

(i) Antitermination is essential for normal expression of hundreds of genes normally active in ESCs. Downregulation of these multiple targets constitutes a profound change in the ESC transcriptome.

(ii) Some of the antitermiation targets are known ESC- and proliferation-specific markers.

(iii) Finally, we added a new experiment where we asked if knockdown of Ammecr1, an Srrt target implicated in normal organismal development and cell proliferation (e.g. Moyses-Oliveira, M. et al. 2018; Ref 45), might phenocopy at least some of the effects triggered by siSrrt. Notably, siAmmecr led to statistically significant upregulation of some of the differentiation markers activated by siSrrt (Fig. S7E-F). How Ammecr1 promotes these changes will be an interesting question for future studies. Of note, another analysis that we added suggests that natural downregulation of the *Ammecr1* gene may be accompanied by an increase in the efficiency of premature termination and the Srrt-regulated iCS (Fig. S7A-D; also discussed in response to Reviewer #1).

"2. In Figure 2C and 4C, there seem to be a number of genes that show intronic CPA but are also up-regulated with siSrrt and siNcbp1. Are these not contradictory outcomes? This is not mentioned in the text and raises a major concern about how expression level is calculated and the conclusions that are based on it. It appears that the up-regulation applies only to the first exon, despite down-regulation throughout the rest of the gene."

We thank the reviewer for this comment, which prompted us to clarify the description of the routine used to calculate gene expression in the Methods. Essentially, we aggregated reads aligning to all annotated exons in a gene using the featureCounts function of the Rsubread R/Bioconductor package and used this an input for the edgeR package (pages 27-28 of the revised manuscript).

It should be noted that genes upregulated despite an increase in intronic cleavage/polyadenylation are clearly a minority compared to their downregulated counterparts: 7 upregulated v 310 downregulated genes in Fig. 2C and 17 upregulated v 475 downregulated genes in Fig. 4C. A typical example of this unusual category is the *Ttll11* gene that is expressed at a very low level in control-treated ESCs (see Fig. R1 below). Knockdown of Srrt promotes accumulation of a prematurely terminated isoform spanning the entire first exon and a part of the first intron. It is possible that this short transcript is more stable that the full-length Ttll11 mRNA in ESCs, since the coverage of the first exon is markedly increased in the siSrrt-treated samples. Alternatively, the *Ttll11* promoter may get activated as an indirect effect of siSrrt. In any case, our pipeline interprets the accumulation of RNA-seq reads in exon 1 with essentially no reads aligning to downstream exons as *Ttll11* upregulation (Fig. R1). This is why this gene ends up in the top right quadrant in Fig. 2C and is highlighted in pink in Table S4. We hope that this example explains the situation.

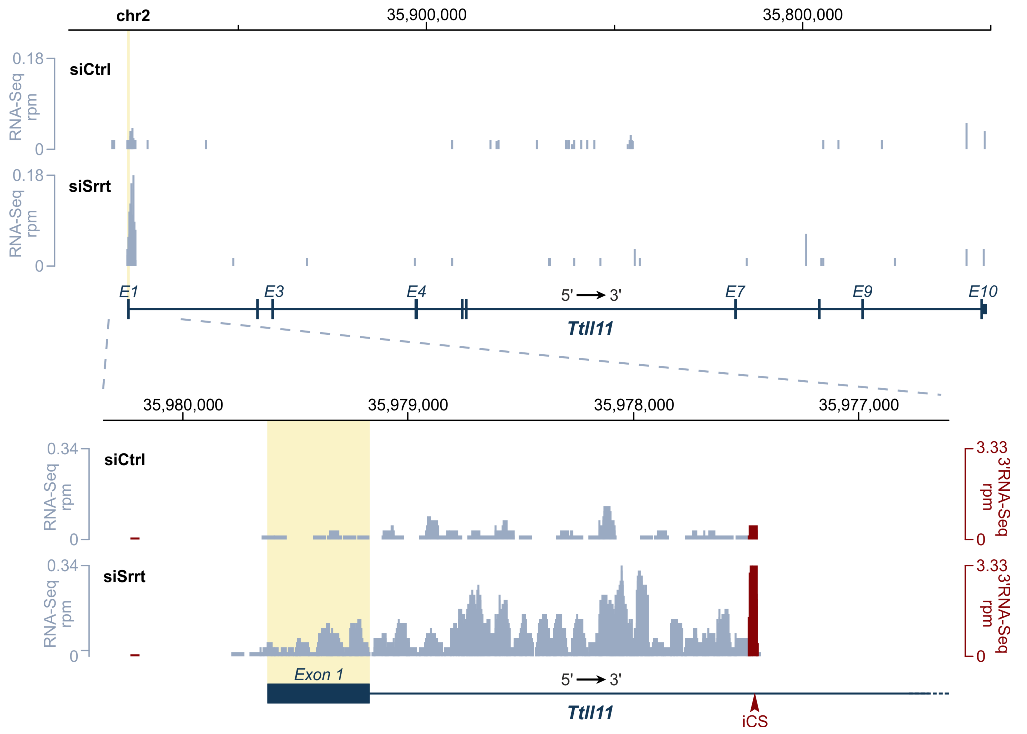

Figure R1. *Top*, RNA-seq coverage plot showing that the *Ttll11* gene is expressed at a low level in siCtrl-treated ESCs and accumulates 5'-proximal RNA-seq reads in response to siSrrt. *Bottom*, RNA-seq and 3'RNA-seq data for the 5'-proximal region of *Ttll11* confirming that siSrrt increases the abundance of iCS-terminated transcripts. The region spanning the first exon is highlighted in yellow.

"3. In Figure 2D, is there any information about already reported PASs in the introns of these example genes? Are these genes ESC-specific or proliferation markers? Is the early termination not a general effect beyond these genes?"

We mention on page 8 of the revised manuscript that only 30.1% of the iCSs activated by siSrrt have been previously reported in the up-to-date polyA_DB3 database (Wang et al. 2018; Ref 48). Thus, the iCSs represent a poorly studied category of cleavage/polyadenylation sites. We also explain on page 7 that the three examples selected for experimental validation in Fig. 2D-E are either known ESC markers (*Cdyl2*; see e.g. the "Epigenetic regulators" category in Fig. 6 of Kalkan et al. 2017; Ref 43) or genes previously suggested to control cell proliferation (*Ammecr1*; Moyses-Oliveira, M. et al. 2018; Ref 45; and *Dcaf6*; Tsai et al. 2005; Ref 47). However, it should be evident from the revised text and Table S4 that regulated iCSs are not limited to ESC- and proliferation-specific markers.

"4. In Figure 2D and throughout, it is necessary to show the RNA-seq reads for the entire gene, or at least a much larger region than just the cap-proximal map."

The revised Fig. S5A shows the entire RNA-seq and 3'RNA-seq profiles for *Ammecr1*, *Cdyl2* and *Dcaf6* genes along with the primers used to validate siSrrt-induced changes in premature termination in these genes.

"5. In Figure S6G, siSrrt does not show any change in precursor U1 snRNA level, however this is not a direct evidence of U1 snRNP level changes. Are U1 snRNP protein amounts changed by siSrrt?"

We have added this and other new U1-related analyses to the revised Fig. S12. The new data strongly support our original conclusions (see also our response to Reviewer #1).

"6. The relevance of the GO terms of genes that are up-regulated by siSrrt and those that are down-regulated to the biological phenomenon must be discussed and explained. How do the functions of the affected genes explain proliferation vs differentiation – it doesn't look like it to me, which reinforces the concern that there is something fundamentally wrong about the data analysis."

To address this concern, we updated relevant parts of the Results (pages 6-7) and Discussion (page 17) and added a new Table S3 with significantly enriched GO terms for upregulated genes. We also hope that the new validation experiments added to the revised version (Fig. 1G and Fig. S2C) will alleviate reviewer's concern about soundness of our data analyses.

"Minor comments:
1. In Figure 1E and 2E, the location of the primers used in these experiments within the gene should be indicated."

The primer annealing sites are now indicated in Fig. S5A.

"2. In Figure 2D, the 3'RACE results should be shown first to validate these products as being from CPA."

The new 3'RACE data shown in Fig. S5B support our original conclusions.

"3. In Figure 2E and 3D-E, using "downstream" and "upstream" instead of "before" and "after" is more correct, and easier to understand, nomenclature."

Done.

"4. An effort should be made to explain, in the text, how gene expression was calculated (ex. from which gene regions)."

The Methods section has been updated accordingly (pages 27-29).

Reviewer #3

"In this study by Kainov and Makeyev, the authors explore the role of the BCB co-factor protein Srrt in ESCs. They find that knockdown of Srrt leads to differentiation of ESCs, and the premature introduction of new 3' ends and poly-adenylation sites and premature transcription termination sites for a subset of transcripts. These new 3' ends are mainly localised inside retrotransposons, and particularly SINEs. I find the study to be mostly well supported, interesting, novel, and suitable for publication in Nature communications. I have a few major comments that the authors should address before the manuscript is ready.

Major points:
1. The claims that Srrt1 knockdown causes differentiation are not convincing. However, in my opinion it is not essential for the paper that this be true, indeed, the lack of robust

differentiation and deregulation of Sox2 is even helpful for the authors as it means that the mechanism is different from NSCs.

The authors may prefer to redevelop this part of the manuscript, in which case I have given some advice below on how they can make this claim more robust. Alternatively, they can delete this part of the manuscript or substantially tone down their claims about differentiation. Dispensing with the claim that Srrt is required for pluripotency, would, in my opinion, still make the manuscript interesting to a wide readership."

We thank the reviewer for these helpful suggestions. As described below, our new analyses confirm that Srrt knockdown triggers partial differentiation of ESCs accompanied by substantial morphological changes and upregulation of several differentiation markers. Although siSrrt fails to change expression of many pluripotency markers, a few of these markers are detectably downregulated. We also modified the text to provide a more nuanced discussion of the role of Srrt in pluripotent stem cells.

"The overall number of differentially regulated genes is quite small for a differentiation process (Fig. S1C). I suspect that techniques like PCA would indicate that the SrrtKD cells are still close to ESCs. It would be useful to reanalyze the data from Refs 39 and 40 and integrate them with the siCtrl and siSrrt data sets to observe the differentiation of the cells. A pair-wise co-correlation, and/or PCA of the RNA-seq samples in this study versus ref 39 and 40 would help understand the nature of the cells differentiation.

Figure 1E: The AP staining changes is not convincing for differentiation, particularly in light of Fig 1G. The authors might be better off with SSEA1 staining which is more sensitive for pluripotency. However, as Nanog is not downregulated (Fig. 1G), it seems more likely the cells are not strongly differentiating."

The following new analyses should address these related concerns about gene expression and morphological changes triggered by Srrt knockdown.

(i) We compared genes changing their expression in response to siSrrt and during spontaneous differentiation using Venn diagrams and Fisher's exact test (new Fig. S2A). This reveals partial but statistically significant overlaps for both downregulated and upregulated genes. We also examined the siCtrl-, siSrrt- and siNcbp1-treated ESCs along with naïve, primed and differentiated samples from Guo et al. 2016 (current Ref 41) by PCA (see Fig. R2 below). This analysis shows that the siCtrl sample is the closest to naive cells and that the siSrrt and siNcbp samples are shifted towards primed and differentiated cells along the PC1 axis and towards primed cells along the PC2 axis. This is consistent with partial differentiation of siSrrt- and siNcbp1-treated ESCs. Since this conclusion can be also drawn from other analyses presented in the revised manuscript and because PCA-based comparisons of RNA-seq data from different studies tend to be confounded by strong batch effects, we did not to include Fig. R2 in the revised manuscript.

(ii) We added a new colony formation assay strengthening our original AP staining experiments (Fig. S1C-F and Fig. 1E-F). We also carried out flow cytometry analyses for the SSEA1 and Pecam1/CD31 markers (Fig. S1G-H). siSrrt-induced downregulation of SSEA1 is modest but statistically significant. Pecam1 is downregulated in a somewhat more robust manner, consistent with our RT-qPCR data in Fig. 1G.

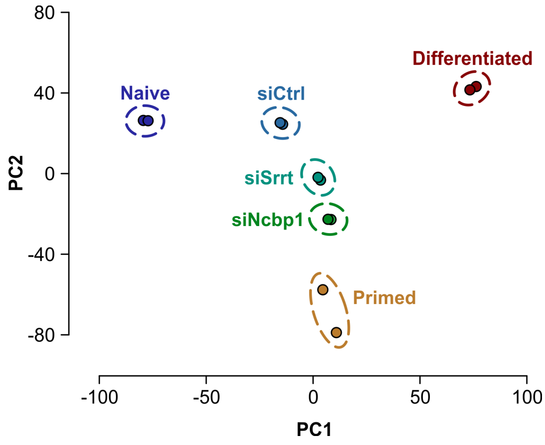

Figure R2. PCA comparison of the siCtrl, siSrrt and siNcbp1 treated samples in our study with published data for naïve, primed and spontaneously differentiated ESCs. Dashed lines encircle duplicated points for each condition.

"Fig 1F: The expression of a wider panel of pluripotency genes should be analysed, taken from the RNA-seq data. Also include 2i and serum+LIF genes. A few heatmaps would be helpful here to support this claim."

As suggested, we added relevant heatmaps (Fig. S2B) and new validation experiments (Fig. 1G and Fig. S2C).

"Fig. S1E: Should be a Venn diagram, also showing the number of genes DE in the spontaneously differentiating cells."

Done - see Fig. S2A.

"A wider panel of somatic-specific genes should be analysed. For example the collagens, other genes involved in somatic state, Jun, Ets-family TFs, also the naive and ground state TFs, EpiSC and early gastrulation genes etc."

Please see new Fig. 1G and Fig. S2B-C.

"Cell surface genes, and particularly the epithelial/mesenchymal state of the cells should be shown. From the AP staining (Fig 1E) the cells appear to be mesenchymal like, and simultaneously pluripotent (a few examples of this phenomena have been published). Expression of the cadherins should be explored. I notice that Pecam1 is down-regulated."

Our analyses include some cell surface markers including immunostaining for Pecam1/CD31 (Fig. 1G, Fig. S1G-H, Fig. S2B-C).

"Fig 1F: The presence of Lefty1 and Lefty2 is strange here, as they should be extremely low or even absent as the cells were cultured in 2i medium, and so being down-regulated further should be very difficult. Similarly, these two genes are early markers for differentiation and would be expected to go up if the cells were differentiating. For example, both genes are robustly expressed in EpiSCs and should be unregulated in the authors spontaneously differentiated cells."

We analyzed Lefty 1 and 2 in the original version since they have been reported by Kalkan et al. 2017 (the "Tgfb" category in Fig. 6 of Kalkan et al. 2017; Ref 43) to be expressed in ESCs and downregulated during differentiation. However, we agree with the reviewer that these genes are better known for their role later in development. To avoid possible confusion, we removed relevant RT-qPCR data from the revised version.

"Apart from this, I only have a few other comments on the rest of the manuscript, which is convincing, well prepared and analysed.

2. Fig 4B: The manuscript says that "…analyses revealed a strong correlation between the siNcbp1- and the siSrrt-treated samples…". But the figure shows a Pearson r of 0.74, which is actually a pretty low correlation when comparing all genes in two samples. It suggests that the Ncbp1 and Srrt KDs are different. The authors could address this by showing that the Srrt is a subset of the Ncbp KD data using a Venn (this seems quite likely), or explain more clearly why they think these samples are similar. I would also be interested in seeing the PCA for Srrt, Ncbp1 KDs versus the controls and the differentiated ESC samples used in this study."

Fig. 4B shows correlation between siSrrt- and siNcbp1- induced changes in the iCS usage. The correlation between gene expression changes triggered by these two treatments is actually higher, r=0.85 (Fig. S10A). Fig. R2 above also shows that the siSrrt and siNcbp1 samples cluster together. Nonetheless, we agree with the reviewer that "strong correlation" may be a misleading term and have modified Results (page 11) and Fig. 4B (page 39) and Fig. S10A legends (page 60) accordingly.

"3. Page 16: "The number of genes that require Srrt for their normal expression in ESCs approaches the size of gene regulation networks controlled by Pou5f1, Nanog, and Sox2 [Tables S2 and S3; 38]. This may explain, at least in part, why Srrt is indispensable for preimplantation development of mouse embryos". I find this argument a bit odd. Down-regulation of any of Pou5f1, Sox2 or Nanog causes very rapid changes in gene expression, as thousands of genes are deregulated, and measuring the size of gene regulation networks is a very arbitrary procedure, prone to difficulty. Ref 38 is an odd choice here, as it barely mentions gene expression networks. Whilst the best characterized network study in pluripotency (Dunn SJ, EMBO J 2018) is not cited. I suggest delete this comment, which seems to be trying to indirectly link Srrt to pluripotency regulation."

The problematic part has been modified (page 17) and the missing reference added.

"Minor comments:
In addition, I have a few minor comments which appear to be errors, or are unclear:

Fig 3C: The figure panel and figure legend says 'RT-qPCR Srrt', but the text says it is RT-qPCR for Ammerc1? Appears to be a figure labelling mistake."

The error has been corrected (page 9).

"Fig 2E and 3C: The magnitude of effect in this experiment is quite different, even though the WT part is the same experiment as in Figure 2E."

The difference must be due to the fact that we use parental A2Lox ESCs in Fig. 2E and a ΔPAS passage-matched WT clone in Fig. 3C. We modified the Fig. 3C legend to reflect this nuance.

"Fig S6E, F: Surely these should include the matching plots for the siSrrt?"

As requested, the siCtrl and siSrrt curves are now overlaid in the new Fig. S11B-D. To make this comparison more informative, we show input-normalized RAP-seq data for all introns and first introns regulated by siSrrt.

Reviewer #4

"In the manuscript titled "A transcriptome-wide antitermination mechanism sustaining identity of embryonic stem cell", Kainov and Makeyev et al dissected the function of an RNA binding protein Srrt (also known as Ars2) in embryonic stem cells. They found that knocking down Srrt led to premature termination of hundreds of transcripts at cryptic cleavage/polyadenylation sites. Mechanistically, SRRT protein may promote the recruitment of U1 snRNP to prevent premature termination at normal ESCs through CBCs. Finally, they found that SRRT regulated cleavage/polyadenylation sites in first intron enriched for retrotransposable elements. Based on their experiments, they made conclusion that Srrt is a molecular guardian of pluripotent state that likely played a profound role in evolution of mammalian genome. The manuscript is well written. The finding of Srrt inhibiting premature termination is novel. I support the publication of the manuscript after a few major concerns are addressed:

1. The claim that Srrt sustains the identity of embryonic stem cells is only based on not so clear decrease of alkaline phosphatase staining. More experiments have to be performed to support this claim. SSEA1 staining, immunofluorescence of pluripotency markers and colony formation assay should be performed. If possible, a reporter cell line (e.g. Rex1-GFP or Oct4-GFP) should be used for siSrrt experiments. Western analysis of OCT4, SOX2 and NANOG instead of RT-qPCR of these genes should be performed."

The following new experiments have been added to address these comments (also see our responses to other reviewers).

(i) Colony formation assays (Fig. S1C-F and Fig. 1E-F).

(ii) Flow cytometry analyses for SSEA1 and Pecam1 (Fig. S1G-H).

(iii) Immunoblot analyses of Oct4, Sox2 and Nanog in ESCs treated with the strongest Srrt-specific siRNA (Fig. S6D-E).

(iv) New RNA-seq data analyses and RT-qPCR data (Fig. 1G and Fig SA-C)

We did not use Rex-GFP and Oct4-GFP lines because neither of these two genes appears to be affected by siSrrt (Fig. 1G).

"2. During differentiation, Srrt RNA and protein level are significantly downregulated. If the hypothesis of this study is correct, one would expect that at least some of transcripts altered in

siSrrt embryonic stem cells are also prematurely terminated during differentiation or in differentiated cells. The authors need check this. These experiments will also help to clarify whether there is a link between Srrt and the identity of embryonic stem cells."

Consistent with reviewer's prediction, we see accumulation of prematurely terminated Ammecr1 transcripts during neuronal differentiation (new Fig. S7A-D). We note that this effect may not be readily detectable for all iCS-terminated transcripts, especially those with relatively short half-lives.

"3. Only one siSrrt is used throughout the manuscript. To exclude possible off-target effects, at least some of experiments need to be repeated with different siRNAs or different knocking down approaches (e.g. CRISPRi)."

The required controls are now shown in Fig. S6 (also see our responses to other reviewers).

"4. CBC was reported to recruit Srrt. In this study, the authors suggest that Srrt promote U1SnRNP binding through CBC. Does Srrt promote the binding of CBC to related transcripts? RIP using antibodies against CBC components followed by qPCR of related transcripts should be performed in siCtrl and siSrrt cells. In addition, is there any evidence that SRRT directly interacts with CBC in embryonic stem cells? Is this interaction dependent on RNA?"

We thank the reviewer for raising these important points. Our new co-immunoprecipitation assays confirm that Srrt can interact with the CBC in mouse ESCs and that this interaction does not depend on a nucleic acid adaptor (Fig. S10E). The newly added RIP data show that siSrrt does not alter the ability of Ncbp1 to interact with (pre-)mRNAs (Fig. S10F). This suggests that Ncbp1 may be required for recruiting Srrt to its targets but not the other way around.

"5. The claim on the role of Srrt in evolution of mammalian genome is rather broad and very significant. Is there any evidence that Srrt plays similar functions in inhibiting RTEs in other mammals, particularly human?"

Answering this question will require a separate line of experiments going beyond the scope of our current work. We have toned down relevant parts of the manuscript to avoid over-interpretation. For instance, we now say that Srrt "may have played a profound role in evolution of mammalian genomes" in the Summary instead of the original "likely played". The Introduction now ends with "important roles in ESC biology and genome evolution", not "fundamental roles", etc.

"Minor concerns:
1. Gene, RNA and protein names should be standardized. For example, for western, SRRT instead of Srrt should be used."

We had to reserve the all-capitals name "SRRT" for the human transgene that we use in Fig. 4D-F. In general, we use non-italicized versions of gene names to refer to their RNA and protein products and additionally provide commonly used synonyms whenever appropriate (Srrt/Ars2, Pou5f1/Oct4, etc).

"2. All western should be performed for at least 3 times and quantified. In addition, protein markers should be shown in all panels."

All westerns have now been replicated and quantified. We also provide primary data with marker lanes clearly labeled in the source data file.

"3. "There were 284 genes with intronic CS (iCS) upregulated ≥2-fold, FDR<0.05 and expression level reduced ≥1.5-fold, FDR<0.05, and an even larger number of genes showing this trend was detected when using less stringent cutoffs (Table S3)." in the main text, however, in Figure 2C, 307 genes are labelled."

The 284 genes were shortlisted based on both the iCS usage fold change (FC≥2) and the false discovery rate (FDR<0.05), whereas the 307 genes in the bottom right quadrant of Fig 2C are selected based on FDR<0.05 only (see Fig. 2C legend). We had to use the "normalized change in relative CS efficiency" statistic described in the Methods when plotting the data because of the large spread in the FC values including several instances of division by 0 (i.e. infinity). This streamlined the presentation but made it difficult to implement the FC filter in a graphically meaningful way.

"4. Figure 3D and E, data should be normalized to WT, siCtrl to demonstrate the impact of delta PAS on the expression of Ammecr1."

Done as requested (see Fig. 3D-E). The upstream and downstream signals are somewhat different between control-treated WT and ΔPAS cells, consistent with a weak basal activity of the PAS even in the presence of normal amounts of Srrt. However, this does not change our original conclusion about the importance of this intronic element for the Srrt-dependent regulation of the *Ammecr1* gene.

"5. Figure 4B legends, top left should be "top right"."

Corrected.

"6. Figure 4D, quantification of western is required. In addition, for Srrt overexpression, lane 4 and 5 should be significantly higher than lane 1 and 2. Lane 6 should be lower than lane 4 and 5 due to knocking down of endogenous SRRT. However, by just looking at these western images, the data presented seem not as expected, especially that lane 6 seem to have similar level of SRRT as in lane 4 and 5."

We have repeated the experiment 3 times and now show quantification of the data in Fig. S10D. The quantification shows that the Srrt signal in lane 6 is somewhat weaker compared to lanes 4 and 5. We now show a shorter-exposure image in Fig. 4D to make this effect more evident.

"7. Figure 6. Why amoU2 also significantly decreased read through efficiency? In addition, the knockdown efficiency of amoU1 and amo should be checked."

The effect of amoU2 on Ammecr1 iCS readthrough actually failed to reach statistical significance (p=0.056 in comparison with amoCtrl; Fig. 6A). Nonetheless, we discuss possibility that decreased splicing efficiency of first introns (i.e. the expected effect of amoU2) may provide a kinetic advantage for intronic cleavage/polyadenylation on page 19 of

the revised manuscript. Further work will be required to understand relative contributions of telescripting- and splicing to Srrt-dependent antitermination on a transcriptome-wide scale. Regarding the second part of this comment, we followed a previously optimized protocol (Kaida et al. 2010; Ref 31) where cells are treated with U1- and U2-specific antisense morpholino oligonucleotides (AMOs) for just 8 hours to avoid cytotoxicity and other possible secondary effects. Under these conditions, the AMOs prevent interactions of U1 and U2 with their pre-mRNA binding sites rather than knocking down their expression levels.

"8. "Importantly, these effects were 7 not due to altered expression of the key pluripotency factors Nanog, Pou5f1, and Sox2 (Fig. 1F)." Fig. 1F should be Fig.1G. "

This part of the revised manuscript has been substantially modified in response to reviewers' comments (page 7).

"9. For Dicer experiment, the expression of key miRNAs should be checked to make sure that miRNA biogenesis is blocked."

A very helpful comment. Using a Qiagen miRCURY LNA RT-qPCR kit we found out that knockdown of Dicer1 (a.k.a. Dicer) leads to significant but relatively modest downregulation of miR-19b-3p and miR-291-3p, two microRNAs expressed in ESCs at readily detectable levels (Fig. R3 below). This suggests that residual expression of Dicer1 in siDicer1-treated cells may be sufficient to produce a bulk of cellular microRNAs. Since this makes our original comparison inconclusive, we turned to published RNA-seq data for Dicer1 knockout in mouse ESCs with a validated effect on microRNA activity (Zheng et al. 2014; Ref 49). Importantly, the gene expression effects of Srrt knockdown and Dicer1 knockout showed no global correlation (new Fig. S9A) and the expression of Srrt-regulated genes did not generally change in response to Dicer knockout (new Fig. S9B-C). These new analyses shown in the revision instead of the original RT-qPCR panels strongly suggest that the activation of the iCSs in siSrrt-treated cells is not a result of deficient production of small noncoding RNAs.

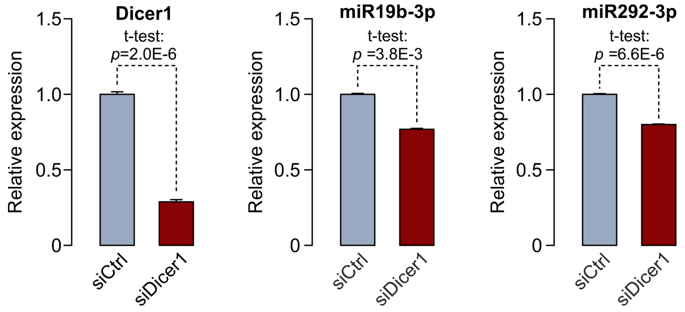

Figure R3. RT-qPCR analyses of the effect of Dicer1 knockdown in mouse ESCs on the expression of mature microRNAs miR-19b-3p and miR-292-3p. Data are averaged from 3 experiments ±SD and compared by a two-tailed t-test.

"10. Titles for supplementary figures should be added."

Done.

REVIEWERS' COMMENTS:

Reviewer #1 (Remarks to the Author):

The authors have addressed my comments.

Reviewer #2 (Remarks to the Author):

The authors have addressed many of the primary concerns raised during the first round of review with additional experiments and revisions to the text.
The explanation for the genes that show increased intronic CPA yet are up-regulated following siSrrt is adequate, but should be included either in the main text or the methods. There are likely to be other readers with the same qualms, and this explanation will help greatly.

Reviewer #3 (Remarks to the Author):

The authors have addressed all of my comments, and should be commended for the thorough way in which they have revised the paper.

Reviewer #4 (Remarks to the Author):

The authors have addressed all my concerns. I suggest to publish the study.

REVIEWERS' COMMENTS

Reviewer #1 (Remarks to the Author):
The authors have addressed my comments.

No response is required.

Reviewer #2 (Remarks to the Author):
The authors have addressed many of the primary concerns raised during the first round of review with additional experiments and revisions to the text. The explanation for the genes that show increased intronic CPA yet are up-regulated following siSrrt is adequate, but should be included either in the main text or the methods. There are likely to be other readers with the same qualms, and this explanation will help greatly

We thank the reviewer for constructive comments. We added the following sentence to the Results section to explain this observation: "Genes upregulated despite the activation of iCSs were clearly a minority, and the increase in the overall expression levels in this case tended to be due to accumulation of prematurely terminated isoforms (e.g. the *Ttll11* gene in Supplementary Data 4)."

Reviewer #3 (Remarks to the Author):
The authors have addressed all of my comments, and should be commended for the thorough way in which they have revised the paper.

No response is required. We are happy that the reviewer liked our approach to the revision process.

Reviewer #4 (Remarks to the Author):
The authors have addressed all my concerns. I suggest to publish the study.

No response is required.
